# Exercise against cocaine sensitization in mice: a [^18^F]fallypride micro-PET study

**DOI:** 10.1093/braincomms/fcab294

**Published:** 2021-12-15

**Authors:** Guillaume Becker, Louis-Ferdinand Lespine, Mohamed Ali Bahri, Maria Elisa Serrano, Christian Lemaire, André Luxen, Ezio Tirelli, Alain Plenevaux

**Affiliations:** GIGA—Cyclotron Research Center—In Vivo Imaging, University of Liège, 4000 Liege, Belgium; Department of Psychology, University of Liège, 4000 Liege, Belgium; Pôle MOPHA, Pôle Est, Centre Hospitalier Le Vinatier, Bron, France; GIGA—Cyclotron Research Center—In Vivo Imaging, University of Liège, 4000 Liege, Belgium; GIGA—Cyclotron Research Center—In Vivo Imaging, University of Liège, 4000 Liege, Belgium; GIGA—Cyclotron Research Center—In Vivo Imaging, University of Liège, 4000 Liege, Belgium; GIGA—Cyclotron Research Center—In Vivo Imaging, University of Liège, 4000 Liege, Belgium; Department of Psychology, University of Liège, 4000 Liege, Belgium; GIGA—Cyclotron Research Center—In Vivo Imaging, University of Liège, 4000 Liege, Belgium

**Keywords:** exercise, cocaine sensitization, mice, dopamine, micro-PET

## Abstract

Wheel-running exercise in laboratory rodents (animal model useful to study the neurobiology of aerobic exercise) decreases behavioural markers of vulnerability to addictive properties of various drugs of abuse including cocaine. However, neurobiological mechanisms underpinning this protective effect are far from fully characterized. Here, 28-day-old female C57BL/6J mice were housed with (*n* = 48) or without (*n* = 48) a running wheel for 6 weeks before being tested for acute locomotor responsiveness and initiation of locomotor sensitization to intraperitoneal injections of 8 mg/kg cocaine. The long-term expression of sensitization took place 3 weeks after the last session. On the day after, all mice underwent a micro-PET imaging session with [^18^F]fallypride radiotracer (dopamine 2/3 receptors antagonist). Exercised mice were less sensitive to acute and sensitized cocaine hyperlocomotor effects, such attenuation being particularly well marked for long-term expression of sensitization (*η*^2^*P* = 0.262). Chronic administration of cocaine was associated with a clear-cut increase of [^18^F]fallypride binding potential in mouse striatum (*η*^2^*P* = 0.170) while wheel-running exercise was associated with a moderate decrease in dopamine 2/3 receptors density in striatum (*η*^2^*P* = 0.075), a mechanism that might contribute to protective properties of exercise against drugs of abuse vulnerability.

## Teaser

Using micro-PET with [^18^F]fallypride radiotracer, Becker *et al*. report that cocaine (COC) sensitization is associated with an increase of [^18^F]fallypride binding potential in the mouse striatum, while exercise is associated with a decrease in D2/3R density, a mechanism that might contribute to protective properties of exercise against COC vulnerability.

## Graphical Abstract

**Graphical Abstract:**
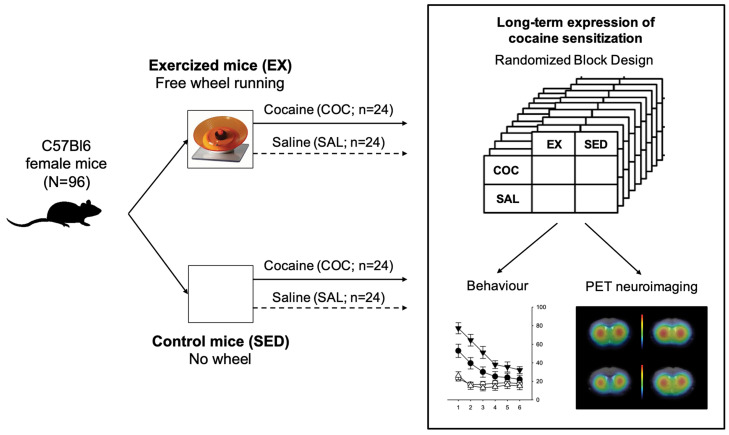

## Introduction

Epidemiological studies have reported a negative association between physical exercise or sports participation and the initiation of drugs of abuse.^1–3^ Research using animal models useful for the study of addiction has provided causal evidence for preventive effects of physical activity on drugs of abuse vulnerability. In rodent studies, physical exercise is often modelled using a freely available running wheel placed in the housing cages. With such a paradigm, exercised (Ex) rats exhibited reduced rates of acquisition, motivation or escalation of self-administration of cocaine (COC), heroin, methamphetamine or speedball, when compared with sedentary (SED) animals.^4–9^ Wheel-running exercise has also been shown to be effective at reducing the acute and chronic locomotor-stimulating effects of COC as well as the sensitization to those effects,^10,11^ a phenomenon that has been implied in the shift from recreational use to pathological abuse, i.e. in the pathogenesis of addiction^12,13^ and suggested to play a role in animal models of drug self-administration.^14–16^ However, the neurobiological mechanisms that underlie this relationship are far from fully understood.

*In vivo* neuroimaging has greatly impacted our understanding of the pathophysiology of the chronic brain state of addiction.^17–19^ Drugs of abuse are known to induce brain plasticity, notably, through changes in neurotransmitter release as well as in pre- and post-synaptic signal transduction. These neuroadaptations underlying the pharmacology of addiction disorders were described particularly for glutamate, through AMPA and metabotropic receptors, and GABA neurotransmissions.^20–27^ Monoaminergic systems are the main targets of psychostimulant drugs like amphetamine and COC. Notably, dopamine (DA) signalling has received considerable attention. Imaging studies have evidenced a decreased dopamine D2 receptors availability in the dorsal striatum of patients suffering from COC addiction.^28^ Preclinical imaging investigations on non-human primates have shown similar results following COC self-administration.^29,30^ In rodents, however, consequences of repeated exposure to COC on the D2 receptor availability remain unclear, with reports of increases,^31,32^ decreases^33^ or unchanged D2 receptor availability.^34,35^ Some of these discrepancies may be due to differences in experimental designs and protocols (e.g. doses and routes of injection, duration of drug withdrawal and timing of the expression of sensitization). It has been further hypothesized that chronic amphetamine or COC administration could result in an increase in D2 high-affinity state receptors density with the unchanged total amount of receptors (low and high-affinity states).^36,37^

Within this specific context of sensitization to COC in mice, the purpose of the present study was first to reproduce our previous behavioural results in C57BL/6J mice. In fact, we found that the effectiveness of wheel-running exercise at attenuating COC locomotor sensitization not only resisted to exercise cessation but was also unambiguously persistent.^38^ Further on, we reported observations suggesting that early-life periods such as early adolescence (versus early adulthood) may be particularly sensitive to protective properties of this form of exercise against vulnerability to COC-induced locomotor sensitization.^39^ The second purpose of the current study was to investigate the neuro-functional correlates of such protective properties by assessing dopamine D2/3 receptors (D2/3R) availability with [^18^F]fallypride micro-PET. We aimed to test whether the protective effect of exercise on COC locomotor sensitization was associated with a modified D2/3R availability.

## Materials and methods

### Subjects

Ninety-six 21-day-old C57BL/6J female mice were obtained from JANVIER, Le-Genest-Saint-Isle, France. The choice of C57BL/6J strain was based on its extensive use in addiction research and previous experiments performed in our laboratory. Given available resources, we did not investigate sex-related differences in the interaction between exercise and COC responsiveness in favour of statistical power (i.e. through higher sample size). Females were preferred over males as they may receive more benefits from exercise.^39–42^ Upon arrival, mice were housed in groups of eight in large transparent polycarbonate cages (38.2 × 22 cm surface × 15 cm height; TECHNIPLAST, Milano, Italy) for 1 week of acclimation. On the following day, they were housed individually according to the experimental housing conditions [exercise or SED receiving COC or saline (SAL) during testing] in smaller TECHNIPLAST transparent polycarbonate cages (32.5 × 17 cm surface × 14 cm height), with pine sawdust bedding, between-animal visual, olfactory and acoustic interactions remaining possible. Tap water and food (standard pellets, CARFIL QUALITY, Oud-Turnhout, Belgium) were continuously available. The animal room was maintained on a 12:12 h light–dark cycle (lights on at 07.00 a.m.) and at an ambient temperature of 20–23°C. All the experimental treatments and animal maintenance were reviewed by the University of Liège Animal Care and Experimentation Committee (animal subjects review board), which gave its approval according to the Belgian implementation of the animal welfare guidelines laid down by the European Union (‘Arrêté Royal relatif à la protection des animaux d’expérience’ released on 23 May 2013 and ‘Directive 2010/63/EU of the European Parliament and of the Council of 22 September 2010 on the protection of animals used for scientific purposes’). This study is reported according to the Animal Research Reporting *In Vivo* Experiments (ARRIVE) guidelines.^43^

### Exercise paradigm

**Figure 1:**
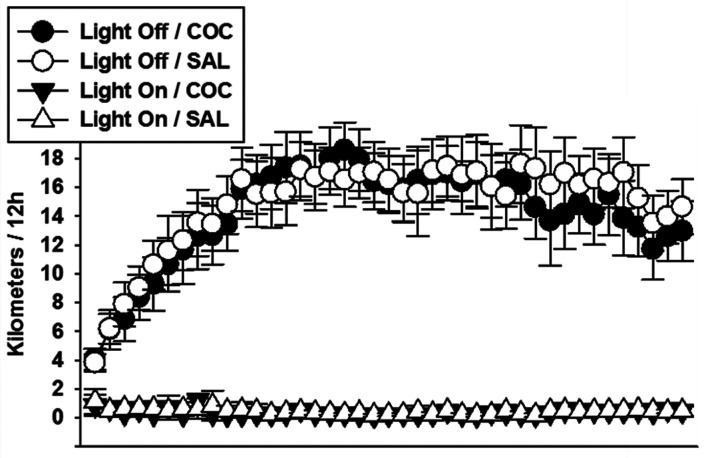
**Wheel-running activity recorded prior to the testing period**. Nocturnal (light off) and diurnal (light on) wheel-running activity of mice randomly assigned to exercise conditions. Since at this stage of the experiment, mice from the COC (*n* = 24) and SAL (*n* = 24) groups were still undistinguishable, no inferential statistics were conducted on these data. All mice showed a rapid increase in wheel-running over the two first weeks until reaching a plateau. Bars represent 95% confidence intervals.

The hardware and software materials used in this study were previously described.^38,39^ Briefly, the running wheels, made of polycarbonate disc which allows an open running surface, were mounted on a plastic base and inclined at a 35° angle (ENV-044, Med Associates; St Albans, VT, USA). The base was fixed on a stable transparent acryl-glass plate. The free-wheel-running activity was recorded permanently during the entire pre-testing period (42 days). The data of each wheel were transferred in real time to a USB interface hub (DIG-804, Med Associates) via a wireless system which relayed data to a Wheel Manager Software (SOF-860, Med Associates). Descriptive statistics of wheel-running activity are displayed in Fig. 1.

### Drug treatments

(−)-Cocaine hydrochloride (BELGOPIA, Louvain-La-Neuve, Belgium) was injected, via the peritoneal route, at the dose of 8 mg/kg and dissolved in 0.01 ml/g of body weight of isotonic SAL solution (0.9% NaCl). An equal volume of isotonic SAL solution served as the control treatment. These parameters, based on our previous studies,^38,39^ were also used in a conditioned place preference test to induce rewarding-like effects in mice.^44^

### Behavioural test chambers

Mice locomotor activity was measured using a pool of eight chambers (one mouse being tested per chamber) and the data were collected through the connection to a custom-written software. These activity chambers were made of a removable transparent polycarbonate tub (22 × 12 cm surface × 12 cm height), fixed on a stable base constituted of a black-paint wooden plank. Infrared light-beams, located on the two long sides of the tub (at 2 cm heights from the floor, 8 cm apart and spaced 6.5 cm from each end of the tub), emanated from two photocell sources and detectors which were mounted on the plank. The locomotor activity was measured in terms of crossings, one crossing being counted every time a mouse broke successively the two parallel beams. During the testing, sound-attenuated shells artificially ventilated and illuminated by a white light bulb, individually encased the activity chambers. Each shell door comprised a window allowing periodic surveillance during testing.

### [^18^F]fallypride radiosynthesis

The radiotracer [^18^F]fallypride was synthetized according to a method previously reported by Brichard *et al*.^45^ with slight modifications. Briefly, the no-carrier-added synthesis of [^18^F]fallypride was conducted by nucleophilic substitution with [^18^F]fluoride of the *p*-toluenesulphonyl group of the commercially available precursor (ABX, Advanced Biochemical Compounds, Radeberg, Germany). After the labelling reaction that was conducted in acetonitrile (1 ml) with 3.5 mg of the substrate at 120°C for 5 min, the crude reaction mixture was diluted with water (6 ml) and the resulting solution injected on a semi-preparative HPLC column. The purification was carried out at 254 nm using a Phenomenex Luna C18 column (5 μm, 250 × 15 mm) at a flow rate of 7 ml/min with an isocratic eluent of water/acetonitrile/trietylamine (45:55:0.1%; retention time of 22 min). The subsequent formulation step^46^ was realized by passing the HPLC collection solution, previously diluted with sodium chloride 0.9% (30 ml) and sodium ascorbate (30 mg) through a tC18 cartridge (360 mg, Waters). [^18^F]fallypride was then eluted from the support with ethanol (1 ml) and diluted with an isotonic solution (6 ml) containing sodium ascorbate (10 mg) as a stabilizer. Based on the starting activity recovered from the cyclotron (111 GBq), this process afforded batches of [^18^F]fallypride ready for subsequent dilution for animal injection. The radiochemical yield was 32 ± 5% (mean ± SD, decay corrected; *n* = 24). At the end of a beam, the averaged specific activity was 49 ± 20.4 Ci/µmol (1813.6 ± 755.6 GBq/µmol, decay corrected, *n* = 24) and the synthesis duration of about 50 min. All the process was automated on a FASTlab synthesizer from GE Healthcare with single-use components.

### [^18^F]fallypride micro-PET imaging data acquisition and processing

Twenty-four [^18^F]fallypride micro-PET imaging sessions were completed and all necessary efforts were made to systematically repeat the same procedure. Anaesthesia was induced with 4% of isoflurane, afterward the mice were placed prone in a dedicated bed. Anaesthesia was maintained with 1–2% isoflurane in a mixture of air and oxygen (30%) at 0.6 L/min. A stereotaxic holder (Minerve, Esternay, France) was systematically used to reduce head movements. Respiratory rate and rectal temperature were permanently measured using a physiological monitoring system (Minerve, Esternay, France). The temperature was maintained at 37 ± 0.5°C, using an air warming system.

[^18^F]fallypride was administered as a bolus intravenous injection in the lateral tail vein over 20 s with a mean injected activity of 12.4 ± 3 MBq (range: 4.9–19.1 MBq). The mean injected mass of fallypride was 0.29 ± 0.35 µg (range: 0.02–2.51 µg). At the time of injection, dynamic micro-PET scans over 60 min were acquired in list-mode using a Siemens Concorde Focus 120 micro-PET (Siemens, Munich, Germany) and followed by 10 min transmission measurement with ^57^Co point source. The list-mode emission data were histogrammed into 3D sinograms by Fourier rebinning and reconstructed by filtered backprojection with a ramp filter cut-off at the Nyquist frequency. All corrections were applied except for scatter events.^47^ No partial volume correction was performed on the acquired data. A set of 3D images was reconstructed in a 256 × 256 × 95 matrix and a zoom factor of 2. The reconstructed voxel size was 0.4 mm × 0.4 mm × 0.8 mm. The dynamic time framing was set as follows: 6 × 5, 6 × 10, 3 × 20, 5 × 30, 5 × 60, 8 × 150, 6 × 300 s and all data were decay corrected to the beginning of each individual frame.

Immediately after PET acquisition, the anaesthetized mice were transferred into a 9.4 T MRI DirectDrive VNMRS horizontal bore system with a shielded gradient system (Agilent Technologies, Palo Alto, CA, USA). A 72 mm inner diameter volumetric coil and a two-channel head surface coil (Rapid Biomedical GmbH, Würzurg, Germany) were used as transmitter and receiver coils, respectively. The 3D anatomical T_2_-weighted brain images were acquired with a fast spin-echo multislice sequence using the following parameters: TR/TEeff = 2500/40 ms, matrix = 128 × 128 × 64, FOV = 20 mm × 20 mm × 10.5 mm, voxel size: 0.156 mm × 0.156 mm × 0.164 mm and a total acquisition time of 21 min.

Imaging data were processed with PMOD software (version 3.7, PMOD Technologies Ltd, Zurich, Switzerland). The processing includes a manual rigid co-registration of individual MRI images to its corresponding PET images, a spatial normalization of the co-registered MRI onto the PMOD MRI template and the extraction of the PET time activity curves of the left and right striatum as well as the cerebellum. Briefly, the inverse deformation parameters obtained during the spatial normalization of the individual MRI images onto the PMOD template were used to bring the mouse brain atlas into the native dynamic PET space and then extract the time activity curves (TACs) based on the atlas predefined structures.^48,49^ The extracted TACs were then transferred into the kinetic modelling module of PMOD in order to estimate the [^18^F]fallypride binding. The non-displaceable binding potential (BP_ND_) parameter was calculated using the multi-linear reference tissue model 2 with the cerebellum as reference tissue.^50,51^ We controlled the homogeneity of the TACs in the reference region (i.e. cerebellum) between each group, to rule out any bias from radiotracer inputs variations in the reference region. The statistical analysis revealed no difference between the four groups of the study for the cerebellum [^18^F]fallypride TACs expressed as area under the curve (AUC) (Supplementary Fig. 1).

### Experimental design and procedure

**Figure 2:**
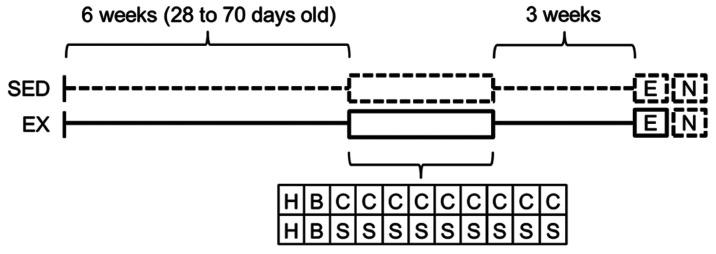
**Experimental timeline and design**. At 28 days of age, 96 mice were housed individually either in the presence (EX, *n* = 48) or the absence (SED, *n* = 48) of a running wheel. Testing began after 6 weeks in these housing conditions (from 28 to 70 days old). The experiment comprised four groups, mice from each housing group (EX or SED) receiving either cocaine or saline (with *n* = 24 per group). Solid lines represent the presence of a running wheel in the home-cage and dotted lines its absence. H, habituation session (to familiarize animals to the novelty of the test context without neither injection nor measures); B, baseline session; the second once-daily session assessing the baseline activity under saline; C, cocaine intraperitoneal administration (nine once-daily sessions); S, control animals receiving saline intraperitoneal administration (nine once-daily sessions); E, session on which the expression of the sensitization was assessed 21–23 days after the last sensitizing injection and under the previous pharmacological treatments; N, neuro-functional measures 24 h after the test of expression (micro-PET).

Experimental timeline and design are presented in Fig. 2. Ninety-six mice were housed in Ex (*n* = 48) or SED (*n* = 48) conditions from 28 days of age and kept in these conditions until the end of behavioural experimentation. Since mice from the two housing environments received COC or SAL during testing, a basic 2 (housing conditions: EX versus SED) × 2 (pharmacological treatment: COC versus SAL) factorial design was generated with *N* = 96, *n* = 24 per group based on preliminary results indicating increasing and decreasing effects of COC (versus SAL, *η*^2^*P* = 0.162) and exercise (versus SED, *η*^2^*P* = 0.093), respectively, on [^18^F]fallypride BP_ND_. Note that experimental procedures and parameters associated with psychopharmacological tests were similar to those used in Lespine and Tirelli,^39^ where continuously Ex females C57BL/6J were found to be less vulnerable than their SED counterparts to acute and sensitized locomotor responsiveness to COC.

Testing included the following phases. (i) A habituation session to familiarize animals to novelty of the test context without neither injection nor measurements. (ii) A session recording baseline locomotor activity where all animals received a SAL solution. (iii) Nine once-daily administrations of COC or SAL. (iv) A final session measuring expression of sensitization, 21–23 days after the last COC administration (animals received their previous respective pharmacological treatment). All sessions lasted 30 min. Experimental blinding was not possible because the experimenter inevitably knew the housing condition and the pharmacological treatment of each mouse. (v) Twenty-four hours after the test of expression of sensitization, mice underwent [^18^F]fallypride micro-PET scan. Note that the wheels (for Ex mice) were removed 24 h before neuroimaging measurement to avoid any potential effect of overnight wheel-running exercise on neuro-functional measures.

Due to practical reasons, the whole experiment was organized into 12 lots purchased and tested successively (each lot consisting of eight mice). In each lot, two mice were assigned to one of the four experimental groups by means of a computer-generated randomization schedule, the eight mice housed in acclimation cages contributing to the four possible groups (SED/COC, SED/SAL, Ex/COC and Ex/SAL). Therefore, the four groups were systematically represented within each lot by two mice to consider any between-lot variability as well as that due to time and circumstances of testing (i.e. randomized block design, Supplementary Fig. 2). Additionally, due to impracticality to test eight mice in a row on the same micro-PET scanning session, each block (*n* = 8) was further split into two blocks (*n* = 4) for the test for expression of sensitization and micro-PET imaging procedures. Again, the four groups were systematically represented within each block by one mouse (Supplementary Fig. 2). Therefore, mice were tested for expression of sensitization either 21 (half) or 23 (other half) days after the last COC injection, while all mice underwent neuroimaging scan 24 h after this test. Note that the order of neuroimaging sessions was counterbalanced across subjects to avoid potential bias due to the specific activity variations. Experimenters conducting neuroimaging testing and analysis were blinded to experimental groups.

### Statistical analysis

Inferential statistics were performed on the following scores. (i) Acute responsiveness to locomotor-activating effects of COC, scored as the difference between values derived from the first COC session and those of the baseline session. (ii) Overall responsiveness to locomotor-activating effects of COC over the initiation of sensitization (nine sessions) scored as the AUC with respect to zero (AUC ground; calculation formula is based on and detailed by Pruessner *et al.*^52^). (iii) Locomotor activity during the expression of sensitization. (iv) The bilateral [^18^F]fallypride BP_ND_ from the left and right striatum of each subject was averaged to give a single [^18^F]fallypride BP_ND_ value per subject.

Each set of data was treated according to a randomized block design with a fixed-model 2 × 2 ANOVA incorporating the housing condition (EX or SED; two levels) and pharmacological treatment (COC or SAL; two levels) as between-group factors (four groups with *n* = 24 each) and with the lot as a blocking factor (with 12 or 24 levels for the behavioural and neuro-functional measures, respectively, see Supplementary Fig. 2). The primary outcomes, defined by our *a priori* hypotheses described below, were given by planned crossed or simple contrasts providing better protection against Type II error.^53^ Each contrast was derived from the mean-square error (MSE) term provided by the ANOVA. Based on previous experiments or preliminary results, Ex mice were expected to display (i) lower COC locomotor responsiveness than SED mice (crossed contrasts) and (ii) lower values of BP_ND_ (simple contrast). COC-receiving mice were expected to show (i) greater locomotor activity and (ii) higher BP_ND_ values than control SAL mice (simple contrasts). Correlations were also computed to determine whether the amount of wheel-running displayed before testing was associated with COC behavioural or neuro-functional outcomes and whether behavioural COC outcomes were associated with neuro-functional measures. Nocturnal distances over the 42-day pre-testing period were averaged for each (Ex) mouse, the resulting individual value serving as the measure of the overall distance travelled on the wheel. Effect sizes were given by *η*^2^*P*, Pearson correlation coefficient *r* and probability of superiority (PS) where appropriate.^54^ Statistical significance threshold was set at 0.05. Statistical analyses were performed using Statistica software version 13.0.

### Data availability

OPTION 1: The authors confirm that the data supporting the findings of this study are available within the Supplementary material.

## Results

### Psychopharmacological measures

**Figure 3:**
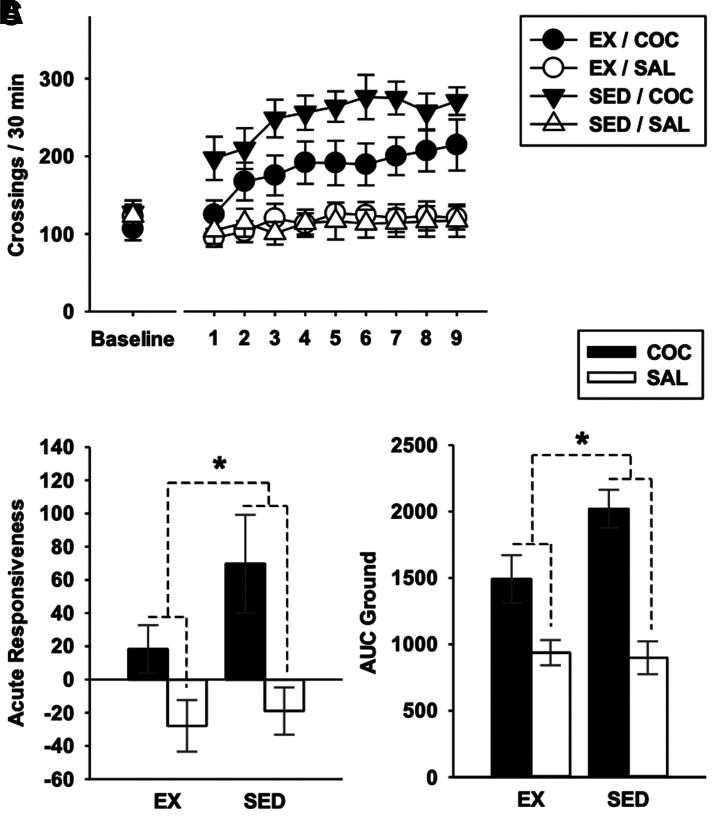
**Acute responsiveness and initiation of sensitization**. (**A**) Baseline locomotor activity (under saline) and initiation of locomotor sensitization over nine once-daily sessions. (**B**) Acute responsiveness scored as the difference between values from the first and baseline sessions. (**C**) Overall locomotor responsiveness over the initiation of sensitization scored as AUC ground. Each set of data was treated according to a randomized block design with a fixed-model 2 × 2 ANOVA incorporating the housing condition (EX or SED) and pharmacological treatment (COC or SAL) as between-group factors (four groups with *n* = 24 each), and with the lot as a blocking factor (with 12 levels, see Supplementary Fig. 2). *Significant planned crossed contrast derived from the MSE provided by the ANOVA, i.e. interaction-related difference between the cocaine effect observed in exercised mice (EX/COC, *n* = 24 versus EX/SAL, *n* = 24) and that measured in sedentary mice (SED/COC, *n* = 24 versus SED/SAL, *n* = 24) taken at a threshold of 0.05. Bars represent 95% confidence intervals.

**Figure 4:**
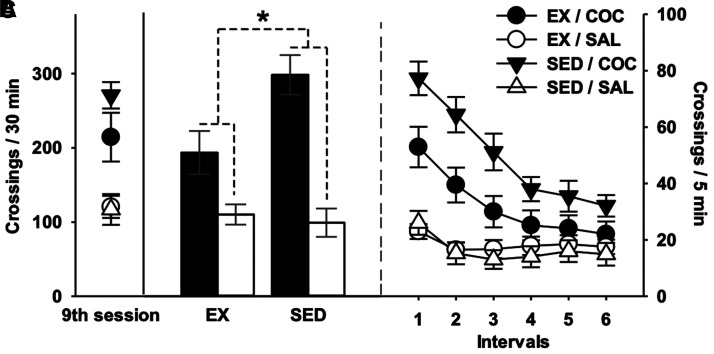
**Long-term expression of sensitization**. (**A**) Locomotor responsiveness on the last (ninth) once-daily session (descriptive statistics). (**B**) Locomotor responsiveness on the test for expression of sensitization. Data were treated according to a randomized block design with a fixed-model 2 × 2 ANOVA incorporating the housing condition (EX or SED) and pharmacological treatment (COC or SAL) as between-group factors (four groups with *n* = 24 each), and with the lot as a blocking factor (with 12 levels, see Supplementary Fig. 2). *Significant planned crossed contrast derived from the MSE term provided by the ANOVA, i.e. interaction-related difference between the cocaine effect observed in exercised mice (EX/COC, *n* = 24 versus EX/SAL, *n* = 24) and that measured in sedentary mice (SED/COC, *n* = 24 versus SED/SAL, *n* = 24) taken at a threshold of 0.05. (**C**) Time-course of locomotor responsiveness during the test for expression of sensitization (descriptive, no inferential statistics were conducted on these data). Bars represent 95% confidence intervals.

Figure 3A depicts baseline locomotor activity and initiation of COC locomotor sensitization. Figure 3B shows scores of acute reactivity. COC locomotor effect (versus SAL, i.e. hyperlocomotor effect) was strong in each group (*t*s_(81)_ = 3.85 and 7.39 with a PS of 72 and 88% in Ex and SED groups, respectively). However, this effect was significantly lower in Ex mice (planned contrasts: *η*^2^*P* = 0.073, *t*_(81)_ = 2.51, *P* = 0.007). Figure 3C depicts AUC ground (i.e. score of overall responsiveness during the initiation of sensitization). The pattern of results was comparable to that found for acute responsiveness. While COC effect was strong in each group (*t*s_(81)_ = 5.47 and 11.07 with PS of 80% and 96% in the Ex and SED groups, respectively), it was much lower in Ex mice (planned contrasts: *η*^2^*P* = 0.162, *t*_(81) _= 3.96, *P* < 0.001). Figure 4 presents locomotor activity on the last (ninth) session of sensitization (Fig. 4A, descriptive statistics) and on the test for expression of sensitization (Fig. 4B and C). Consistent with previous experimental stages, long-term expression of the sensitized locomotor responsiveness was largely reduced in Ex mice (planned contrasts: *η*^2^*P* = 0.262, *t*_(81)_ = 5.37, *P* < 0.001). Again, COC effect was unambiguous in each group (*t*s_(81)_ = 5.48 and 13.08 with PS of 80% and 98% in the Ex and SED groups, respectively). Table 1 reports relationships between amounts of exercise and behavioural outcomes and [^18^F]fallypride BP_ND_. Wheel-running exercise was strongly and positively associated with AUC ground in COC-receiving mice. However, this result should be cautiously interpreted in the context of other correlations results, number of tests performed and sample size. Importantly, AUC ground was strongly and positively associated with the expression of sensitization. The fact that wheel-running distances were strongly associated with AUC ground yet correlated weakly to the expression of sensitization questions the nature of relationships reported between exercise and AUC ground (i.e. risk of false-positive).

**Table 1:** Relationships between averaged pre-testing running distances and behavioural and neuroimaging outcomes [Pearson’s coefficient (*P*-value)]

	Acute responsiveness	AUC ground	Expression of sensitization	[^18^F]fallypride BP_ND_
COC	−0.17 (0.43)	0.43 (0.035)	0.15 (0.50)	−0.24 (0.33)^a^
SAL	−0.11 (0.61)	−0.10 (0.66)	−0.03 (0.89)	−0.09 (0.75)^b^

^a^ *n* = 19.

^b^ *n* = 16, *n* = 24 otherwise.

### [^18^F]fallypride micro-PET neuroimaging

**Figure 5:**
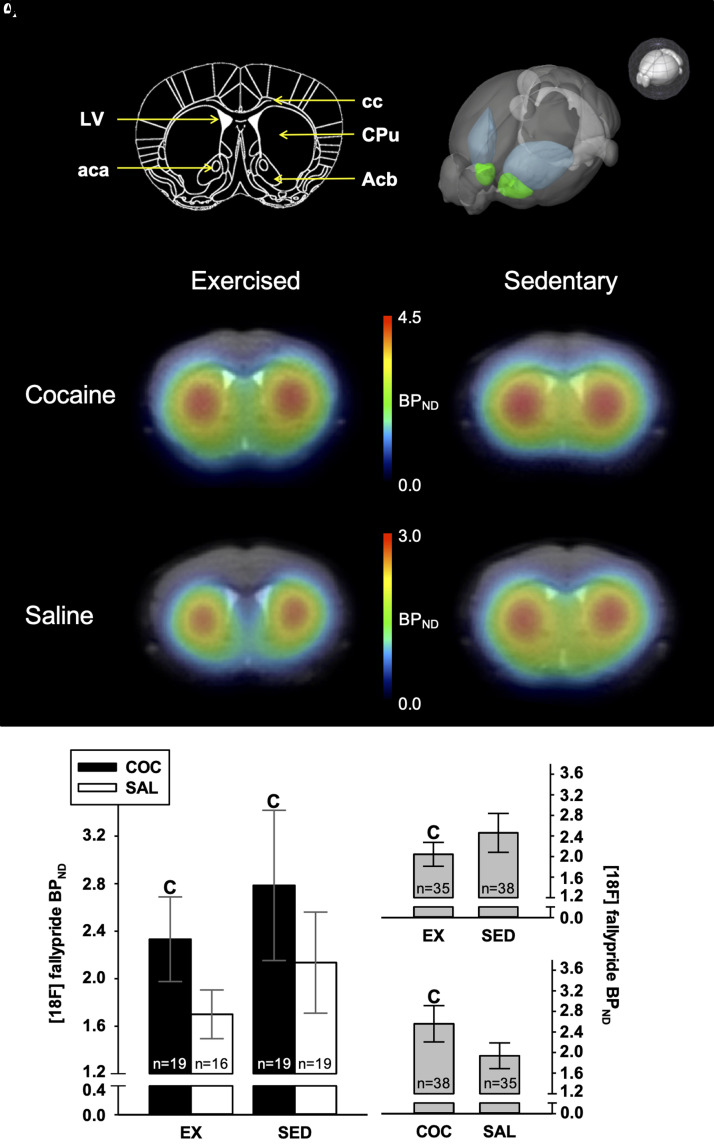
**Neuroimaging outcomes**. (**A**) Left upper panel: coronal slice image of the mouse brain at Bregma 0.8 mm, based on the mouse Atlas of Franklin and Paxinos.^55^ Right upper panel: 3D depiction of regions of interest showing the Caudate-Putamen (in blue) and the Nucleus Accumbens (in green). *Bottom*: representative [^18^F]fallypride BP_ND_ images of mice of the four groups, co-registered to their corresponding individual anatomical MRI. Note the difference in scales between the cocaine groups and the saline groups. (**B**) Micro-PET-derived [^18^F]fallypride BP_ND_ measured 24 h after expression of sensitization in exercised and sedentary mice. (**C**) Marginal means associated with the effect of housing conditions. (**D**) Marginal means associated with the effect of pharmacological treatment. Data were treated according to a randomized block design with a fixed-model 2 × 2 ANOVA incorporating the housing condition (EX or SED) and pharmacological treatment (COC or SAL) as between-group factors, and with the lot as a blocking factor (with 24 levels, see Supplementary Fig. 2). Cs indicate significant planned simple contrast derived from the term (MSE provided by the ANOVA, i.e. difference compared to the corresponding control group, taken at a threshold of 0.05. Bars represent 95% confidence intervals.

Figure 5A displays representative [^18^F]fallypride BP_ND_ images of mice of the four groups. Figure 5B presents [^18^F]fallypride BP_ND_ measured in the striatum 24 h after the expression of sensitization in Ex (EX/COC and EX/SAL) and SED (SED/COC and SED/SAL) mice. Due to technical problems (fails in proper intravenous radiotracer delivery at injection time), data from 23 mice (over 96) were not acquired or useable (EX/COC: *n* = 5; EX/SAL: *n* = 8; SED/COC: *n* = 5 and SED/SAL: *n* = 5). Note that the exclusion of those animals from behavioural analysis led to a similar pattern of results (reported in Supplementary Table 1). Figure 5C and D presents the marginal means associated with main effects of housing conditions (EX: *n* = 35; SED: *n* = 38) and pharmacological treatment (COC: *n* = 38; SAL: *n* = 35), respectively. We found evidence for a moderate attenuating effect of aerobic exercise on [^18^F]fallypride BP_ND_ (*η*^2^*P* = 0.075, PS = 65, *t*_(50)_ = 2.01, *P* = 0.024). Additionally, COC-receiving mice exhibited higher [^18^F]fallypride BP_ND_ in striatum than their SAL counterparts as supported by a large effect of pharmacological treatment (*η*^2^*P* = 0.170, PS = 74, *t*_(50)_ = 3.20, *P* = 0.001). However, crossed contrasts indicated that the interaction between housing conditions and the pharmacological treatment was clearly negligible (*η*^2^*P* < 0.005, *t*_(50)_ = 0.20, *P* = 0.42). Table 2 reports relationships between behavioural outcomes and [^18^F]fallypride BP_ND_. There was no evidence for an association between behavioural outcomes and BP_ND_ values.

**Table 2:** Relationships between behavioural outcomes and neuroimaging outcomes expressed as [^18^F]fallypride BP_ND_ [Pearson’s coefficients (*P*-value)]

	EX/COC^a^	EX/SAL^b^	SED/COC^a^	SED/SAL^a^
Acute responsiveness	−0.28 (0.25)	−0.26 (0.34)	0.05 (0.85)	−0.28 (0.25)
AUC ground	−0.17 (0.48)	0.17 (0.52)	0.28 (0.24)	0.37 (0.12)
Expression of sensitization	0.20 (0.41)	0.06 (0.81)	0.005 (0.98)	0.27 (0.27)

^a^ *n* = 19.

^b^ *n* = 16.

## Discussion

The main findings of the present study can be summarized as follows. (i) Previous results obtained in our laboratory were reproduced by showing that wheel-running exercise-induced preventive effects against acute and chronic locomotor responsiveness to a rewarding dose of COC (8 mg/kg) in female C57BL/6J mice.^11,38^ (ii) The COC-sensitized brain, at the time of long-term expression, revealed a striatal increase in dopamine D2/3 receptors availability measured by [^18^F]fallypride micro-PET. (iii) Wheel-running was associated with attenuated dopamine D2/3 receptors availability measured by [^18^F]fallypride micro-PET.

Our behavioural results are consistent with previously reported findings showing that male Wistar rats continuously housed with a wheel expressed little or no sensitized locomotor activity 15 days after five once-daily injections of 10 mg/kg COC.^10^ Another study showed that continuous wheel-running exercise was effective at reducing the locomotor-activating effects of 3 and 10 mg/kg COC in Long–Evans females.^56^ Such attenuating effect was also observed in C57BL/6J mice housed in large home-cages comprising a running wheel as part of a composite housing environment made of inanimate objects and conspecifics.^57,58^ More generally, our results add to extensive preclinical literature reporting preventive consequences of wheel-running exercise on behavioural markers of sensitivity to addictive properties of drugs of abuse.^2^

Physical exercise is known to act on DA system and to possess rewarding properties, like COC and other drugs of abuse.^59,60^ For instance, Greenwood *et al.*^60^ reported that Fischer344 rats exercising with a running wheel for 6 weeks preferred a compartment paired with the aftereffect of exercise. They also showed that a 6-week continuous access to wheels resulted in increases in ΔFosB/FosB immunoreactivity in the nucleus accumbens (Acb), tyrosine hydroxylase (TH) mRNA levels in the ventral tegmental area (VTA) and delta-opioid receptor mRNA levels in the Acb shell, whereas dopamine receptor D2 mRNA is reduced in the Acb core. It is thus tempting to ascribe the exercise-induced protective effects against the COC-induced locomotor sensitization to its neuroplastic effects on dopaminergic neurotransmission. To our knowledge, our study is the first to report *in vivo* PET imaging of mouse striatum D2/3R under aerobic exercise in the context of COC sensitization. First, we observed that long-term expression of COC sensitization is associated with an increase of D2/3R availability in the mouse striatum. Those results are in line with studies suggesting that psychomotor sensitization to a stimulant is linked to enhanced D2/3R availability which may explain the high locomotor response of psychostimulant sensitized mice to direct-acting D2 agonists.^31,32,61,62^ Although previous reports hypothesized that the behavioural sensitization could occur through an increase of the dopamine D2 high-affinity state receptors availability,^36,63,64^ the [^18^F]fallypride radiotracer is not able to discriminate between the two affinity states of D2 receptors. Therefore, our results must not rely on the proportion of high- versus low-affinity state but rather on the total amount of available receptors. Our work could be interpreted in the light of the supposed enhanced activity of DA neurons in the VTA during COC sensitization, where the increased D2/3R availability in the striatum may contribute to the enhanced locomotor response induced by repeated COC injections.^65^ Vanderschuren and Kalivas^66^ well reviewed the adaptations of DA transmission associated with the long-term expression of sensitization. They highlighted that COC-sensitized synapses showed increased release of DA and increased sensitivity of DA receptors. In our protocol, imaging sessions took place 24 h after the last exposure to COC, we suggest that our results are related to an increased D2/3R density rather than a decrease in synaptic DA.

Second, we observed a decreased D2/3R availability in the striatum of Ex mice compared to their SED counterparts. This is in accordance with previous results showing that wheel-running induced a reduction of D2R mRNA in Fisher 344 rats Acb core.^60^ The authors also reported an increased TH mRNA level in the VTA leading them to propose that voluntary exercise can increase the synthetic capacity of DA in the striatum.^60,67^ These modifications could be related to neuroplastic events induced by an enhanced expression of ΔFosB transcription factor in the Acb nucleus.^60^ Interestingly, a sustained accumulation of ΔFosB in the Acb nucleus was also described following chronic COC exposure, as well as for others drugs of abuse.^68–70^ This common feature led to the assumption that neuroplasticity induced by voluntary exercise could alter DA neurotransmission in the mesolimbic reward pathway, which may contribute then to the beneficial effects of exercise on COC sensitization.^60^ This is supported by data reporting that chronically Ex Sprague–Dawley rats displayed a lower DA release and a hampered DA reuptake, under amphetamine challenge.^71^ Physical exercise has been shown to reduce the basal level of DA in the rat striatum, which accord to the hypothesis of a reduced DA tone resulting from chronic physical exercise despite an enhanced synthetic capacity.^72^ Besides, our current results are coherent with the study of Fisher *et al.*^73^ reporting a reduced dopamine D2 mRNA expression as a result of an exercise in basal ganglia of C57Bl6 mice. We suggest that voluntary physical exercise-induced neuroplastic changes, among others on DA mesolimbic reward pathway partly through a reduced D2/3R availability in the mouse striatum. This trait might participate to render Ex mice more resilient to psychomotor effects of COC. However, our current data do not allow us to unravel the mechanism of decreased D2/3R availability which can be related to a decreased D2/3R density or an increase in endogenous synaptic DA. Further studies are warranted to investigate the persistence of the impact of physical exercise on D2/3R availability similarly to what has been done in behavioural assessment, as well as the drug-induced plasticity we described in the present study.

Some limitations warrant mention. One of those relies on the single-sex design. We cannot rule out the possibility that male C57BL/6J mice could display a different pattern of results. Regarding the imaging results, the [^18^]Fallypride radiotracer displays a nearly equal affinity for D2 and D3 DA receptors *in vivo*^74^; hence, the [^18^]Fallypride BP_ND_ parameter primarily reflect a combination of signals from D2 and D3 receptors. It has been suggested that up to 20% of the [^18^]Fallypride binding may be due to D3R *in vivo*.^74^ This is of relevance considering that D3R is highly expressed in the mesolimbic DA system and involved in the pathophysiology of addiction.^75^ Strikingly, their expression seemed to be increased in nicotine behavioural sensitization.^76^ Determining the respective proportion of D2R and D3R in our present results would definitely need further investigations. The second technical limitation relies on the spatial resolution of the micro-PET scanner, which is ∼1.5 mm^2^ in our setup.^47^ This has of course to be taken into account when attempting to achieve *in vivo* molecular imaging of the mouse brain. The mouse brain atlas implemented in Pmod 3.7, derived from the work of Mirrione *et al.*,^47^ do not allow to analyse separately the ventral and the dorsal parts of the striatum, the Acb and the Caudate-Putamen (CPu), respectively. As a consequence, the region of interest (ROI) analysed to compute the [^18^F]fallypride BP_ND_ is constituted by the entire striatum. This is of interest as the DA inputs within the Acb hail from the VTA whereas the DA afferences throughout the CPu originate from the Substancia nigra (SN). On the one hand, DA neurons of the VTA are known to play a major role into the reward and motivation processes^77^ and to their pathological counterpart that is addiction. On the other hand, DA neurons lying in the SN are controlling motor functions. Intuitively, one would think that the [^18^F]fallypride BP_ND_ modifications described here are primarily located in the Acb. However, D2R is involved in locomotor activity and may be implicated in [^18^F]fallypride BP_ND_ variations induced by physical exercise.^78^ Our results may be conflicting with those of Vučcković *et al.*,^78^ reporting an increased [^18^F]fallypride BP_ND_ in a mouse model relevant for the study of Parkinson’s disease under exercise condition. However, the pathophysiological paradigm shift prevents any direct comparison of the data as it has been revealed that exercise has a differential effect on the dopaminergic system in DA terminals depleted mice compared to control mice.^73,79^ One of the main limitations of our study relies on the use of females only. A single-sex experiment in male C57BL/6J mice did not find any evidence for an effect of (treadmill) exercise on [^18^F]fallypride BP_ND_ within the basal ganglia.^78^ Lynch *et al.*^41^ reviewed evidence for the sex-specific differences in the efficacy of exercise against addiction disorders and highlighted that females have an enhanced sensitivity to this protective effect.

The sensitization phenomenon has been achieved in healthy men in laboratory context by Boileau *et al.*^80^ The authors reported an increased DA release after repeated exposure to amphetamine. Of note, in this study, no changes in striatal D2R binding potential were reported following several doses of d-amphetamine. Beyond the interspecies variations, experimental design may account for this discrepancy (e.g. single-sex design with men only in the study of Boileau *et al*.; not the same control groups).

Clinical assessment of D2/3R in methamphetamine users under behavioural intervention with exercise training has been achieved using [^18^F]fallypride PET imaging.^81^ Interestingly, they reported an increased [^18^F]fallypride BP_ND_ in the whole striatum of Ex patients compared to those of the control group (i.e. methamphetamine users under behavioural intervention with educational training). Thus, the authors suggest that under depleted striatal D2/3R availability, as shown by the methamphetamine users, physical exercise may increase the availability of these receptors while pointing out the relatively small sample size. Besides, the absence of healthy control in the study design precludes conclusions drawing on the effect of exercise on striatal D2/3R availability in physiological conditions. Moreover, our design focused on the sensitization process (and the associated long-term expression) which is thought to be a useful model to investigate early events in the natural history of addiction pathophysiology (e.g. recreational use). On the contrary, the work of Robertson *et al.*^81^ examined exercise intervention in patients with an extensive drug history that has been shown to be associated with a decrease in D2/3R availability. Furthermore, patients were under complete abstinence which could impact DA neurochemical processes.

In conclusion, we report a replication study of a protective effect of wheel-running exercise on both initiation and expression of COC locomotor sensitization in female C57BL/6J mice. We provided data as proof-of-concept in female C57BL/6J, showing that exercise-induced neuroplasticity within mesolimbic DA pathway includes a reduced D2/3R availability in the striatum, while COC locomotor sensitization is associated with an increased D2/3R availability in this brain area, and contributing to the neurobiological understanding of physical exercise and its positive impact on addictive behaviours. Further investigations are warranted to unravel the molecular mechanisms by which exercise affects dopaminergic signalling and to characterize sex-related differences in aerobic exercise-induced plasticity and its effects.

## Supplementary Material

fcab294_Supplementary_Data

## Abbreviations

Acb =: accumbens
AUC =: area under the curve
BP_ND_ =: non-displaceable binding potential
CPu =: Caudate-Putamen
COC =: cocaine
DA =: dopamine
D2/3R =: dopamine 2/3 receptors
EX =: exercised
ROI =: region of interest
SAL =: saline
SED =: sedentary
SN =: Substancia nigra
TAC =: time activity curve
VTA =: ventral tegmental area

## References

[1] De La Garza R, Yoon JH, Thompson-Lake DGY, et al. Treadmill exercise improves fitness and reduces craving and use of cocaine in individuals with concurrent cocaine and tobacco-use disorder. Psychiatry Res. 2016;245:133–140. 27541349 10.1016/j.psychres.2016.08.003 PMC5067203

[2] Lynch WJ, Peterson AB, Sanchez V, Abel J, Smith MA. Exercise as a novel treatment for drug addiction: A neurobiological and stage-dependent hypothesis. Neurosci Biobehav Rev. 2013;37(8):1622–1644. 23806439 10.1016/j.neubiorev.2013.06.011 PMC3788047

[3] Lisha NE, Sussman S. Relationship of high school and college sports participation with alcohol, tobacco, and illicit drug use: A review. Addict Behav. 2010;35(5):399–407. 20100638 10.1016/j.addbeh.2009.12.032 PMC3134407

[4] Smith MA, Schmidt KT, Iordanou JC, Mustroph ML. Aerobic exercise decreases the positive-reinforcing effects of cocaine. Drug Alcohol Depend. 2008;98(1–2):129–135. 18585870 10.1016/j.drugalcdep.2008.05.006 PMC2613778

[5] Smith MA, Walker KL, Cole KT, Lang KC. The effects of aerobic exercise on cocaine self-administration in male and female rats. Psychopharmacology. 2011;218(2):357–369. 21567123 10.1007/s00213-011-2321-5 PMC3752981

[6] Smith MA, Pitts EG. Access to a running wheel inhibits the acquisition of cocaine self-administration. Pharmacol Biochem Behav. 2011;100(2):237–243. 21924284 10.1016/j.pbb.2011.08.025 PMC3199311

[7] Smith MA, Pitts EG. Wheel running decreases the positive reinforcing effects of heroin. Pharmacol Rep. 2012;64(4):960–964. 23087148 10.1016/s1734-1140(12)70891-5 PMC3760409

[8] Engelmann AJ, Aparicio MB, Kim A, et al. Chronic wheel running reduces maladaptive patterns of methamphetamine intake: regulation by attenuation of methamphetamine-induced neuronal nitric oxide synthase. Brain Struct Funct. 2014;219(2):657–672. 23443965 10.1007/s00429-013-0525-7 PMC3702684

[9] Lacy RT, Strickland JC, Brophy MK, Witte MA, Smith MA. Exercise decreases speedball self-administration. Life Sci. 2014;114(2):86–92. 25132360 10.1016/j.lfs.2014.08.005 PMC4175302

[10] Renteria Diaz L, Siontas D, Mendoza J, Arvanitogiannis A. High levels of wheel running protect against behavioural sensitization to cocaine. Behav Brain Res. 2013;237:82–85. 22985687 10.1016/j.bbr.2012.09.014

[11] Geuzaine A, Tirelli E. Wheel-running mitigates psychomotor sensitization initiation but not post-sensitization conditioned activity and conditioned place preference induced by cocaine in mice. Behav Brain Res. 2014;262:57–67. 24434305 10.1016/j.bbr.2014.01.002

[12] Robinson TE, Berridge KC. The neural basis of drug craving: an incentive-sensitization theory of addiction. Brain Res Rev. 1993;18(3):247–291. 8401595 10.1016/0165-0173(93)90013-p

[13] Berridge KC, Robinson TE. Liking, wanting, and the incentive-sensitization theory of addiction. Am Psychol. 2016;71(8):670–679. 27977239 10.1037/amp0000059 PMC5171207

[14] Schenk S, Partridge B. Sensitization and tolerance in psychostimulant self-administration. Pharmacol Biochem Behav. 1997;57(3):543–550. 9218279 10.1016/s0091-3057(96)00447-9

[15] Robinson TE, Berridge KC. Addiction. Annu Rev Psychol. 2003;54(1):25–53. 12185211 10.1146/annurev.psych.54.101601.145237

[16] Anderson SM, Pierce RC. Cocaine-induced alterations in dopamine receptor signaling: Implications for reinforcement and reinstatement. Pharmacol Ther. 2005;106(3):389–403. 15922019 10.1016/j.pharmthera.2004.12.004

[17] Wang GJ, Smith L, Volkow ND, et al. Decreased dopamine activity predicts relapse in methamphetamine abusers. Mol Psychiatry. 2012;17(9):918–925. 21747399 10.1038/mp.2011.86 PMC3261322

[18] Volkow ND, Fowler JS, Wolf AP, et al. Effects of chronic cocaine abuse on postsynaptic dopamine receptors. Am J Psychiatry. 1990;147(6):719–724. 2343913 10.1176/ajp.147.6.719

[19] Volkow ND . Is methylphenidate like cocaine? Arch Gen Psychiatry. 1995;52(6):456. 7771915 10.1001/archpsyc.1995.03950180042006

[20] Kalivas PW, Duffy P, Barrow J. Regulation of the mesocorticolimbic dopamine system by glutamic acid receptor subtypes. J Pharmacol Exp Ther. 1989;251(1):378–387. 2552079

[21] Klitenick MA, DeWitte P, Kalivas PW. Regulation of somatodendritic dopamine release in the ventral tegmental area by opioids and GABA: an in vivo microdialysis study. J Neurosci. 1992;12(7):2623–2632. 1319478 10.1523/JNEUROSCI.12-07-02623.1992 PMC6575858

[22] Lu W, Chen H, Xue C-J, Wolf ME. Repeated amphetamine administration alters the expression of mRNA for AMPA receptor subunits in rat nucleus accumbens and prefrontal cortex. Synapse. 1997;26(3):269–280. 9183816 10.1002/(SICI)1098-2396(199707)26:3<269::AID-SYN8>3.0.CO;2-5

[23] Pierce RC, Bell K, Duffy P, Kalivas PW. Repeated cocaine augments excitatory amino acid transmission in the nucleus accumbens only in rats having developed behavioral sensitization. J Neurosci. 1996;16(4):1550–1560. 8778304 10.1523/JNEUROSCI.16-04-01550.1996 PMC6578548

[24] Tanner T . GABA-induced locomotor activity in the rat, after bilateral injection into the ventral tegmental area. Neuropharmacology. 1979;18(5):441–446. 460540 10.1016/0028-3908(79)90067-4

[25] Xue C-J, Ng JP, Li Y, Wolf ME. Acute and repeated systemic amphetamine administration: effects on extracellular glutamate, aspartate, and serine levels in rat ventral tegmental area and nucleus accumbens. J Neurochem. 2002;67(1):352–363. 10.1046/j.1471-4159.1996.67010352.x 8667013

[26] Will BE, Toniolo G, Brailowsky S. Unilateral infusion of GABA and saline into the nucleus basalis of rats: 1. Effects on motor function and brain morphology. Behav Brain Res. 1988;27(2):123–129. 3358849 10.1016/0166-4328(88)90038-1

[27] Abel JM, Nesil T, Bakhti-Suroosh A, Grant PA, Lynch WJ. Mechanisms underlying the efficacy of exercise as an intervention for cocaine relapse: a focus on mGlu5 in the dorsal medial prefrontal cortex. Psychopharmacology. 2019;236(7):2155–2171. 31161451 10.1007/s00213-019-05208-0 PMC6626681

[28] Volkow ND, Fowler JS, Wang G-J, Swanson JM. Dopamine in drug abuse and addiction: results from imaging studies and treatment implications. Mol Psychiatry. 2004;9(6):557–569. 15098002 10.1038/sj.mp.4001507

[29] Nader J, Claudia C, El Rawas R, et al. Loss of environmental enrichment increases vulnerability to cocaine addiction. Neuropsychopharmacology. 2012;37(7):1579–1587. 22334125 10.1038/npp.2012.2 PMC3358749

[30] Nader MA, Czoty PW. PET imaging of dopamine D2 receptors in monkey models of cocaine abuse: genetic predisposition versus environmental modulation. Am J Psychiatry. 2005;162(8):1473–1482. 16055768 10.1176/appi.ajp.162.8.1473

[31] Peris J, Boyson SJ, Cass WA, et al. Persistence of neurochemical changes in dopamine systems after repeated cocaine administration. J Pharmacol Exp Ther. 1990;253(1):38–44. 2329520

[32] Sousa FCF, Gomes PB, Macêdo DS, Marinho MMF, Viana GSB. Early withdrawal from repeated cocaine administration upregulates muscarinic and dopaminergic D2-like receptors in rat neostriatum. Pharmacol Biochem Behav. 1999;62(1):15–20. 9972840 10.1016/s0091-3057(98)00142-7

[33] Maggos C . Sustained withdrawal allows normalization of in vivo [11C]N-methylspiperone dopamine D2 receptor binding after chronic binge cocaine a positron emission tomography study in rats. Neuropsychopharmacology. 1998;19(2):146–153. 9629568 10.1016/S0893-133X(98)00009-8

[34] Claye LH, Akunne HC, Duff Davis M, DeMattos S, Soliman KFA. Behavioral and neurochemical changes in the dopaminergic system after repeated cocaine administration. Mol Neurobiol. 1995;11(1–3):55–66. 8561968 10.1007/BF02740684

[35] Stanwood GD, Lucki I, McGonigle P. Differential regulation of dopamine D2 and D3 receptors by chronic drug treatments. J Pharmacol Exp Ther. 2000;295:1232–1240. 11082460

[36] Briand LA, Flagel SB, Seeman P, Robinson TE. Cocaine self-administration produces a persistent increase in dopamine D2 High receptors. Eur Neuropsychopharmacol. 2008;18(8):551–556. 18284941 10.1016/j.euroneuro.2008.01.002 PMC2527181

[37] Seeman P, Ko F, Tallerico T. Dopamine receptor contribution to the action of PCP, LSD and ketamine psychotomimetics. Mol Psychiatry. 2005;10(9):877–883. 15852061 10.1038/sj.mp.4001682

[38] Lespine L-F, Tirelli E. The protective effects of free wheel-running against cocaine psychomotor sensitization persist after exercise cessation in C57BL/6J mice. Neuroscience. 2015;310:650–664. 26454024 10.1016/j.neuroscience.2015.10.009

[39] Lespine L-F, Tirelli E. Evidence for a long-term protection of wheel-running exercise against cocaine psychomotor sensitization in adolescent but not in adult mice. Behav Brain Res. 2018;349:63–72. 29727710 10.1016/j.bbr.2018.04.054

[40] Lespine L-F, Plenevaux A, Tirelli E. Wheel-running exercise before and during gestation against acute and sensitized cocaine psychomotor-activation in offspring. Behav Brain Res. 2019;363:53–60. 30703395 10.1016/j.bbr.2019.01.049

[41] Lynch WJ, Robinson AM, Abel J, Smith MA. Exercise as a prevention for substance use disorder: a review of sex differences and neurobiological mechanisms. Curr Addict Reports. 2017;4:455–466. 10.1007/s40429-017-0178-3 PMC5802367 29430384

[42] Zhou Y, Zhao M, Zhou C, Li R. Sex differences in drug addiction and response to exercise intervention: From human to animal studies. Front Neuroendocrinol. 2016;40:24–41. 26182835 10.1016/j.yfrne.2015.07.001 PMC4712120

[43] Kilkenny C, Browne WJ, Cuthill IC, Emerson M, Altman DG. Improving bioscience research reporting: the ARRIVE guidelines for reporting animal research. PLoS Biol. 2010;8(6):e1000412. 20613859 10.1371/journal.pbio.1000412 PMC2893951

[44] Brabant C, Quertemont E, Tirelli E. Evidence that the relations between novelty-induced activity, locomotor stimulation and place preference induced by cocaine qualitatively depend upon the dose: a multiple regression analysis in inbred C57BL/6J mice. Behav Brain Res. 2005;158:201–210. 15698886 10.1016/j.bbr.2004.08.020

[45] Brichard L, Ferrari V, Smith R, Aigbirhio FI. Synthesis of [18 F]-fallypride. In: Scott PJH, Hockley BG, eds. Radiochemical syntheses: radiopharmaceuticals for positron emission tomography. John Wiley & Sons, Inc., 2012:95–102.

[46] Lemaire C, Plenevaux A, Aerts J, et al. Solid phase extraction—an alternative to the use of rotary evaporators for solvent removal in the rapid formulation of PET radiopharmaceuticals. J Label Compd Radiopharm. 1999;42(1):63–75.

[47] Bahri MA, Plenevaux A, Warnock G, Luxen A, Seret A. NEMA NU4-2008 image quality performance report for the microPET focus 120 and for various transmission and reconstruction methods. J Nucl Med. 2009;50(10):1730–1738. 19759103 10.2967/jnumed.109.063974

[48] Ma Y, Hof PR, Grant SC, et al. A three-dimensional digital atlas database of the adult C57BL/6J mouse brain by magnetic resonance microscopy. Neuroscience. 2005;135:1203–1215. 16165303 10.1016/j.neuroscience.2005.07.014

[49] Mirrione MM, Schiffer WK, Fowler JS, Alexoff DL, Dewey SL, Tsirka SE. A novel approach for imaging brain–behavior relationships in mice reveals unexpected metabolic patterns during seizures in the absence of tissue plasminogen activator. Neuroimage. 2007;38:34–42. 17707126 10.1016/j.neuroimage.2007.06.032 PMC2084071

[50] Innis RB, Cunningham VJ, Delforge J, et al. Consensus nomenclature for in vivo imaging of reversibly binding radioligands. J Cereb Blood Flow Metab. 2007;27(9):1533–1539. 17519979 10.1038/sj.jcbfm.9600493

[51] Ichise M, Liow J-S, Lu JQ, et al. Linearized reference tissue parametric imaging methods: application to [^11^C]DASB positron emission tomography studies of the serotonin transporter in human brain. J Cereb Blood Flow Metab. 2003;23(9):1096–1112. 12973026 10.1097/01.WCB.0000085441.37552.CA

[52] Pruessner JC, Kirschbaum C, Meinlschmid G, Hellhammer DH. Two formulas for computation of the area under the curve represent measures of total hormone concentration versus time-dependent change. Psychoneuroendocrinology. 2003;28(7):916–931. 12892658 10.1016/s0306-4530(02)00108-7

[53] Rosnow RL, Rosenthal R. Effect sizes. Z Psychol/J Psychol. 2009;217(1):6–14.

[54] Fritz CO, Morris PE, Richler JJ. Effect size estimates: current use, calculations, and interpretation. J Exp Psychol Gen. 2012;141(1):2–18. 21823805 10.1037/a0024338

[55] Franklin K, Paxinos G. The mouse brain in stereotaxic coordinates. In: The mouse brain in stereotaxic coordinates. Elsevier; 2007:xv. https://linkinghub.elsevier.com/retrieve/pii/B9780123742476500043

[56] Smith MA, Witte MA. The effects of exercise on cocaine self-administration, food-maintained responding, and locomotor activity in female rats: Importance of the temporal relationship between physical activity and initial drug exposure. Exp Clin Psychopharmacol. 2012;20:437–446. 22924703 10.1037/a0029724 PMC3752996

[57] Bezard E, Dovero S, Belin D, et al. Enriched environment confers resistance to 1-methyl-4-phenyl-1,2,3,6-tetrahydropyridine and cocaine: involvement of dopamine transporter and trophic factors. J Neurosci. 2003;23(35):10999–11007. 14657156 10.1523/JNEUROSCI.23-35-10999.2003 PMC6741042

[58] Solinas M, Thiriet N, El Rawas R, Lardeux V, Jaber M. Environmental enrichment during early stages of life reduces the behavioral, neurochemical, and molecular effects of cocaine. Neuropsychopharmacology. 2009;34(5):1102–1111. 18463628 10.1038/npp.2008.51

[59] Belke TW . Running and responding reinforced by the opportunity to run: effect of reinforcer duration. J Exp Anal Behav. 1997;67(3):337–351. 9163938 10.1901/jeab.1997.67-337 PMC1284610

[60] Greenwood BN, Foley TE, Le TV, et al. Long-term voluntary wheel running is rewarding and produces plasticity in the mesolimbic reward pathway. Behav Brain Res. 2011;217(2):354–362. 21070820 10.1016/j.bbr.2010.11.005 PMC3021978

[61] Trulson ME, Ulissey MJ. Chronic cocaine administration decreases dopamine synthesis rate and increases [3H] spiroperidol binding in rat brain. Brain Res Bull. 1987;19(1):35–38. 3115498 10.1016/0361-9230(87)90162-6

[62] De Vries T . Relapse to cocaine- and heroin-seeking behavior mediated by dopamine D2 receptors is time-dependent and associated with behavioral sensitization. Neuropsychopharmacology. 2002;26(1):18–26. 11751029 10.1016/S0893-133X(01)00293-7

[63] Seeman P, Tallerico T, Ko F, Tenn C, Kapur S. Amphetamine-sensitized animals show a marked increase in dopamine D2 high receptors occupied by endogenous dopamine, even in the absence of acute challenges. Synapse. 2002;46(4):235–239. 12373738 10.1002/syn.10139

[64] Novak G, Seeman P, Le Foll B. Exposure to nicotine produces an increase in dopamine D2 High receptors: A possible mechanism for dopamine hypersensitivity. Int J Neurosci. 2010;120(11):691–697. 20942582 10.3109/00207454.2010.513462

[65] Liu C-L, Wang Y-K, Jin G-Z, Shi W-X, Gao M. Cocaine-induced locomotor sensitization associates with slow oscillatory firing of neurons in the ventral tegmental area. Sci Rep. 2018;8(1):1–11. 29459754 10.1038/s41598-018-21592-7 PMC5818474

[66] Vanderschuren LJMJ, Kalivas PW. Alterations in dopaminergic and glutamatergic transmission in the induction and expression of behavioral sensitization: a critical review of preclinical studies. Psychopharmacology. 2000;151(2-3):99–120. 10972458 10.1007/s002130000493

[67] Foley TE, Fleshner M. Neuroplasticity of dopamine circuits after exercise: implications for central fatigue. NeuroMolecular Med. 2008;10(2):67–80. 18274707 10.1007/s12017-008-8032-3

[68] Kelz MB, Chen J, Carlezon WA Jr, et al. Expression of the transcription factor deltaFosB in the brain controls sensitivity to cocaine. Nature. 1999;401(6750):272–276. 10499584 10.1038/45790

[69] Pich EM, Pagliusi SR., Tessari M, Talabot-Ayer D, van Huijsduijnen RH, Chiamulera C. Common neural substrates for the addictive properties of nicotine and cocaine. Science 1997;275(5296):83–86. 8974398 10.1126/science.275.5296.83

[70] Atkins JB, Chlan-Fourney J, Nye HE, Hiroi N, Carlezon WA, Nestler EJ. Region-specific induction of ΔFosB by repeated administration of typical versus atypical antipsychotic drugs. Synapse. 1999;33(2):118–128. 10400890 10.1002/(SICI)1098-2396(199908)33:2<118::AID-SYN2>3.0.CO;2-L

[71] Marques E, Vasconcelos F, Rolo MR, et al. Influence of chronic exercise on the amphetamine-induced dopamine release and neurodegeneration in the striatum of the rat. Ann N Y Acad Sci. 2008;1139(1):222–231. 18991868 10.1196/annals.1432.041

[72] Meeusen R, Smolders I, Sarre S, et al. Endurance training effects on neurotransmitter release in rat striatum: an in vivo microdialysis study. Acta Physiol Scand. 1997;159(4):335–341. 9146755 10.1046/j.1365-201X.1997.00118.x

[73] Fisher BE, Petzinger GM, Nixon K, et al. Exercise-induced behavioral recovery and neuroplasticity in the 1-methyl-4-phenyl-1,2,3,6-tetrahydropyridine-lesioned mouse basal ganglia. J Neurosci Res. 2004;77(3):378–390. 15248294 10.1002/jnr.20162

[74] Mukherjee J, Constantinescu CC, Hoang AT, Jerjian T, Majji D, Pan M-L. Dopamine D3 receptor binding of 18 F-fallypride: Evaluation using in vitro and in vivo PET imaging studies. Synapse. 2015;69(12):577–591. 26422464 10.1002/syn.21867 PMC4624469

[75] Volkow ND, Morales M. The brain on drugs: from reward to addiction. Cell. 2015;162(4):712–725. 26276628 10.1016/j.cell.2015.07.046

[76] Le Foll B, Diaz J, Sokoloff P. Increased dopamine D3 receptor expression accompanying behavioral sensitization to nicotine in rats. Synapse. 2003;47(3):176–183. 12494400 10.1002/syn.10170

[77] Salamone JD, Correa M. The mysterious motivational functions of mesolimbic dopamine. Neuron. 2012;76(3):470–485. 23141060 10.1016/j.neuron.2012.10.021 PMC4450094

[78] Vučcković MG, Li Q, Fisher B, et al. Exercise elevates dopamine D2 receptor in a mouse model of Parkinson’s disease: In vivo imaging with [18F]fallypride. Mov Disord. 2010;25(16):2777–2784. 20960487 10.1002/mds.23407 PMC3273304

[79] Petzinger GM, Walsh JP, Akopian G, et al. Effects of treadmill exercise on dopaminergic transmission in the 1-methyl-4-phenyl-1,2,3,6-tetrahydropyridine-lesioned mouse model of basal ganglia injury. J Neurosci. 2007;27(20):5291–5300. 17507552 10.1523/JNEUROSCI.1069-07.2007 PMC6672356

[80] Boileau I, Dagher A, Leyton M, et al. Modeling sensitization to stimulants in humans. Arch Gen Psychiatry. 2006;63(12):1386–1395. 17146013 10.1001/archpsyc.63.12.1386

[81] Robertson CL, Ishibashi K, Chudzynski J, et al. Effect of exercise training on striatal dopamine D2/D3 receptors in methamphetamine users during behavioral treatment. Neuropsychopharmacology. 2016;41(61740-634X):1629–1636. 26503310 10.1038/npp.2015.331 PMC4832026

