# Supplementary material for: Exercise against cocaine sensitization in mice: a [18F]fallypride micro-PET study
Source: Brain Commun. 2021 Dec 15;4(1):fcab294. doi: 10.1093/braincomms/fcab294 (PMC8833578; doi:10.1093/braincomms/fcab294)
Supplement: fcab294_Supplementary_Data [file fcab294_supplementary_data.zip › Supplementary_material.docx]

**Supplementary material**

**Exercise against cocaine sensitization in mice: a [^18^F]fallypride micro-PET study**

Guillaume Becker^1*^, Louis-Ferdinand Lespine^2,3^, Mohamed Ali Bahri^1^, Maria Elisa Serrano^1^, Christian Lemaire^1^, André Luxen^1^, Ezio Tirelli^2^ and Alain Plenevaux^1^

^1^ GIGA – Cyclotron Research Center – In Vivo Imaging, University of Liège, 4000 Liege, Belgium.

^2^ Department of Psychology, University of Liège, 4000 Liege, Belgium.

^3^ Pôle MOPHA, Pôle Est, Centre Hospitalier Le Vinatier, Bron, France

^*^ Corresponding author:

Guillaume Becker

Email: g.becker@uliege.be

Allée du 6 Août, 8. Quartier Agora, B30, 4000 Liège, Belgium.

ORCID ID 0000-0002-1714-0267

Current address:

Laboratoire CarMeN, INSERM U1060, University Lyon1, INRA U1397, INSA Lyon, Hospices Civils Lyon.

Groupement Hospitalier Est, 59 Boulevard Pinel, 69 500 BRON, France

**List of supplemental material:**

**Supplementary Figure 1. Cerebellum TACs (expressed as AUCs) homogeneity between groups.**

**Supplementary Figure 2. Randomized block design.**

**Supplementary Table 1. Comparisons of behavioural data between full samples (n=24/group) and samples without animals with missing neuro-functional data.**

**Supplementary Figure 1.**

**Cerebellum TACs (expressed as AUCs) homogeneity between groups.**

AUCs data set was treated according to a randomized block design with a fixed-model 2 x 2 ANOVA incorporating the housing condition (EX or SED; 2 levels) and pharmacological treatment (COC or SAL; 2 levels) as between-group factors, and with the lot as a blocking factor (with 24 levels)

No evidence for effect of aerobic exercise, cocaine, or interaction:

**AUC** (η²p = 0.05, F(1,46) = 2.610, p = 0.11; η²p = 0.02, F(1,46) = 0.862, p = 0.36; η²p = 0.02, F(1,46) = 0.797, p = 0.38, respectively).

**Supplementary Figure 2.**

**Randomized block design.**

Due to practical reasons, the whole experiment was organized into twelve lots purchased and tested successively (each lot consisting of 8 mice). In each lot, two mice were assigned to one of the four experimental group by means of a computer-generated randomization schedule, the eight mice housed in the acclimation cages contributing to theses four possible groups (sedentary/cocaine, sedentary/saline, exercised/cocaine, and exercised/saline). Therefore, the four groups were systematically represented within each lot by 2 mice to take into account any between-lot variability as well as that due to the time and circumstances of testing (i.e. randomized block design). Additionally, due to impossibility to test 8 mice the same day in micro-PET, each block (n=8) was further split into 2 blocks (n=4) for the test for expression of sensitization and micro-PET procedures. Again, the four groups were systematically represented within each block by one mouse. Therefore, mice were tested for expression of sensitization either 30 (half) or 32 (other half) days after the last cocaine injection, while all mice underwent neuroimaging scan 24h after this test.

**Supplementary Table 1.**

**Comparisons of behavioural data between full samples (n=24/group) and samples without animals with missing neuro-functional data.**

There was **no evidence for statistically significant differences** between the samples (Cohen’s d ranging from 0.02 to 0.45 with p-values ranging from .961 to .189).

Following tables: “Acute responsiveness”: number of crossings during the 1st cocaine injection minus number of crossings during baseline, “AUC ground”: refs for formula are cited in the main text, “Expression of sensitization”: number of crossings during the corresponding session.

Table S1. Mean (SD) — Exercised mice receiving cocaine.

|  | ALL mice (n=24) | n=5 excluded (n=19) | t-value | p-value | Cohen’s d |
| --- | --- | --- | --- | --- | --- |
| Acute responsiveness | 18 (36) | 25 (37) | 0.626 | .535 | 0.19 |
| AUC ground | 1491 (450) | 1504 (470) | 0.092 | .926 | 0.03 |
| Expression of sensitization | 194 (73) | 193 (58) | 0.048 | .961 | 0.02 |

Table S2. Mean (SD) — Exercised mice receiving saline.

|  | ALL mice (n=24) | n=8 excluded  (n=16) | t-value | p-value | Cohen’s d |
| --- | --- | --- | --- | --- | --- |
| Acute responsiveness | -28 (39) | -13 (27) | 1.337 | .189 | 0.45 |
| AUC ground | 937 (237) | 922 (253) | 0.191 | .849 | 0.06 |
| Expression of sensitization | 110 (34) | 113 (36) | 0.267 | .791 | 0.09 |

Table S3. Mean (SD) — Sedentary mice receiving cocaine.

|  | ALL mice (n=24) | n=5 excluded  (n=19) | t-value | p-value | Cohen’s d |
| --- | --- | --- | --- | --- | --- |
| Acute responsiveness | 70 (74) | 76 (77) | 0.259 | .796 | 0.08 |
| AUC ground | 2020 (358) | 2101 (335) | 0.757 | .453 | 0.23 |
| Expression of sensitization | 298 (66) | 303 (71) | 0.238 | .812 | 0.07 |

Table S4. Mean (SD) — Sedentary mice receiving saline.

|  | ALL mice (n=24) | n=5 excluded (n=19) | t-value | p-value | Cohen’s d |
| --- | --- | --- | --- | --- | --- |
| Acute responsiveness | -19 (36) | -18 (39) | 0.087 | .931 | 0.03 |
| AUC ground | 899 (309) | 942 (331) | 0.439 | .662 | 0.13 |
| Expression of sensitization | 99 (48) | 100 (50) | 0.066 | .947 | 0.02 |
