# Supplementary material for: Exercise against cocaine sensitization in mice: a [18F]fallypride micro-PET study
Source: Brain Commun. 2021 Dec 15;4(1):fcab294. doi: 10.1093/braincomms/fcab294 (PMC8833578; doi:10.1093/braincomms/fcab294)
Supplement: fcab294_Supplementary_Data [file fcab294_supplementary_data.zip › Original Submission.pdf]

OXFORD UNIVERSITY PRESS | Brain Communications

Exercise Against Cocaine Sensitization In Mice: A [18F]Fallypride Micro-PET Study

Journal:	*Brain Communications*
Manuscript ID	BRAINCOM-2021-197
Manuscript Type:	Original Article
Date Submitted by the Author:	02-Jun-2021
Complete List of Authors:	Becker, Guillaume; University of Liege, GIGA - CRC In vivo Imaging<br>Lespine, Louis-Ferdinand; University of Liege, Department of Psychology; Groupement Hospitalier Est, Pôle MOPHA<br>Bahri, Mohamed; University of Liege, GIGA-Cyclotron Research Centre<br>Serrano, Maria Elisa; University of Liege, GIGA - CRC In vivo Imaging<br>Lemaire, Christian; University of Liege, GIGA - CRC In vivo Imaging<br>Luxen, André; University of Liege, GIGA - CRC In vivo Imaging<br>Tirelli, Ezio; University of Liege, Department of Psychology<br>Plenevaux, Alain; University of Liege, GIGA - CRC In vivo Imaging
Keywords:	Exercise, Cocaine sensitization, Mice, Dopamine, MicroPET

SCHOLARONE™
Manuscripts

Full title: Exercise Against Cocaine Sensitization In Mice: A [^{18}F]Fallypride Micro-PET Study

Running title: **Exercise And Cocaine Effects On Mice Striatum**

Guillaume Becker (Pharm.D. – Ph.D.)[1,§,*], Louis-Ferdinand Lespine (Ph.D.)[2,3,§], Mohamed Ali Bahri (Ph.D.)[1], Maria Elisa Serrano (Ph.D.)[1], Christian Lemaire (Ph.D.)[1], André Luxen (Ph.D.)[1], Ezio Tirelli (Ph.D.)[2] and Alain Plenevaux (Ph.D.)[1]

[1] GIGA – Cyclotron Research Center – In Vivo Imaging, University of Liège, 4000 Liege, Belgium.

[2] Department of Psychology, University of Liège, 4000 Liege, Belgium.

[3] Pôle MOPHA, Pôle Est, Centre Hospitalier Le Vinatier, Bron, France

[§] These authors contributed equally to this work.

[*] Corresponding author:

Guillaume Becker

Email: g.becker@uliege.be

Allée du 6 Août, 8. Quartier Agora, B30, 4000 Liège, Belgium.

ORCID ID 0000-0002-1714-0267

Current address:

Laboratoire CarMeN, INSERM U1060, University Lyon1, INRA U1397, INSA Lyon, Hospices Civils Lyon.

Groupement Hospitalier Est, 59 Boulevard Pinel, 69 500 BRON, France

For Review Only

ABBREVIATED SUMMARY

Using microPET with [^{18}F]fallypride radiotracer, Becker et al. report that cocaine sensitization is associated with an increase of [^{18}F]fallypride binding potential in the mouse striatum, while exercise is associated with a decrease in D2/3R density, a mechanism that might contribute to protective properties of exercise against cocaine vulnerability.

ABSTRACT

Wheel-running exercise in laboratory rodents (animal model useful to study the neurobiology of aerobic exercise) decreases behavioral markers of vulnerability to addictive properties of various drugs of abuse including cocaine. However, neurobiological mechanisms underpinning this protective effect are far from fully characterized. Here, 28-day-old female C57BL/6J mice were housed with (n=48) or without (n=48) a running wheel for 6 weeks before being tested for acute locomotor responsiveness and initiation of locomotor sensitization to intraperitoneal injections of 8 mg/kg cocaine. The long-term expression of sensitization took place 3 weeks after the last session. On the day after, all mice underwent a micro-PET imaging session with [^{18}F]fallypride radiotracer (dopamine 2/3 receptors antagonist). Exercised mice were less sensitive to acute and sensitized cocaine hyperlocomotor effects, such attenuation being particularly well-marked for long-term expression of sensitization (η^2p = 0.262). Chronic administration of cocaine was associated with a clear-cut increase of [^{18}F]fallypride binding potential in mouse striatum (η^2p = 0.170) while wheel-running exercise was associated with a moderate decrease in dopamine 2/3 receptors density in striatum (η^2p = 0.075), a mechanism that might contribute to protective properties of exercise against drugs of abuse vulnerability.

KEYWORDS

Exercise; cocaine sensitization; mice; dopamine; micro-PET

For Review Only

INTRODUCTION

Epidemiological studies have reported a negative association between physical exercise or sports participation and the initiation of drugs of abuse.[1–3] Research using animal models useful for the study of addiction has provided causal evidence for preventive effects of physical activity on drugs of abuse vulnerability. In rodent studies, physical exercise is often modelled using a freely available running wheel placed in the housing cages. With such a paradigm, exercised rats exhibited reduced rates of acquisition, motivation or escalation of self-administration of cocaine, heroin, methamphetamine or speedball, as compared to sedentary animals.[4–9] Wheel-running exercise has also been shown to be effective at reducing the acute and chronic locomotor-stimulating effects of cocaine as well as the sensitization to those effects[10,11], a phenomenon thought to play an integral role in craving and relapse.[12,13] However, the neurobiological mechanisms that underlie this relationship are far from fully understood.

In vivo neuro-imaging has greatly impacted our understanding of pathophysiology of the chronic brain state of addiction.[14–16] Imaging studies have evidenced a decreased dopamine D2 receptors availability in the dorsal striatum of patients suffering from cocaine addiction.[17] Preclinical imaging investigations on non-human primates have shown similar results following cocaine self-administration.[18,19] In rodents, however, consequences of repeated exposure to cocaine on the D2 receptor availability remains unclear, with reports of increases[20,21], decreases[22] or unchanged D2 receptor availability.[23,24] Some of these discrepancies may be due to differences in experimental designs and protocols (e.g. doses and routes of injection, duration of drug withdrawal, and timing of the expression of sensitization). It has been further hypothesized that chronic amphetamine or cocaine administration could result in an increase in D2 high affinity state receptors density with unchanged total amount of receptors (low and high affinity states).[25,26]

For Review Only

The present study was set-up to enrich our previous behavioral results in female C57BL/6J mice. In fact, we found that the effectiveness of wheel-running exercise at attenuating cocaine locomotor sensitization not only resisted to exercise cessation but was also unambiguously persistent.[27] Further on, we reported observations suggesting that early-life period such as early adolescence (vs early adulthood) may be particularly sensitive to protective properties of this form of exercise against vulnerability to cocaine-induced locomotor sensitization.[28] The purpose of the current study was to go further and investigate the neuro-functional correlates of such protective properties by assessing dopamine D2/3 receptors (D2/3R) availability with [^{18}F]fallypride microPET. We aimed to test whether the protective effect of exercise on cocaine locomotor sensitization was associated with a modified D2/3R availability.

METHODS

Subjects

Ninety-six 21-day-old C57BL/6J females were obtained from JANVIER, Le-Genest-Saint-Isle, France. The choice of C57BL/6J strain was based on its extensive use in addiction research and previous experiments performed in our laboratory. Given available resources, we did not investigate sex-related differences in the interaction between exercise and cocaine responsiveness in favor of statistical power (i.e., through higher sample size). Female were preferred over male as they may receive more benefits from exercise.[28–31] Upon arrival, mice were housed in groups of eight in large transparent polycarbonate cages (38.2 x 22 cm surface x 15 cm height; TECHNIPLAST, Milano, Italy) for one week of acclimation. On the following day, they were housed individually according to the experimental housing conditions (exercise or sedentary receiving cocaine or saline during testing) in smaller TECHNIPLAST transparent polycarbonate cages (32.5 x 17 cm surface x 14 cm height), with pine sawdust bedding, between-animal visual, olfactory and acoustic interactions remaining possible. Tap water and

For Review Only

food (standard pellets, CARFIL QUALITY, Oud-Turnhout, Belgium) were continuously available. The animal room was maintained on a 12:12 h light-dark cycle (lights on at 07.00 a.m.) and at an ambient temperature of 20-23°C. All experimental treatments and animal maintenance were reviewed by the University of Liège Animal Care and Experimentation Committee (animal subjects review board), which gave its approval according to the Belgian implementation of the animal welfare guidelines laid down by the European Union ("Arrêté Royal relatif à la protection des animaux d'expérience" released on 23 May 2013, and "Directive 2010/63/EU of the European Parliament and of the Council of 22 September 2010 on the protection of animals used for scientific purposes"). The ARRIVE guidelines (Animal Research Reporting In Vivo Experiments), which have been developed to improve quality of experimenting and reporting in animals studies, were followed as closely as possible.[32]

For Review Only

Exercise paradigm

A running wheel was made of an orange polycarbonate saucer-shaped disk (diameter 15 cm, circumference 37.8 cm; allowing an open running surface) mounted (bearing pin) on a plastic cup-shaped base (height 4.5 cm) and tilted at a 35° angle from the vertical plane (ENV-044, Med Associates; St Albans, VT, USA). The base was fixed on a stable transparent acryl-glass plate. Running was monitored and recorded continuously during the 42-day pre-testing period via a wireless system, each wheel being connected to a USB interface hub (DIG-804, Med Associates) which relayed data to a Wheel Manager Software (SOF-860, Med Associates). Data dealing with wheel-running activity are shown in Fig. 1.

Drug treatments

(−)-Cocaine hydrochloride (BELGOPIA, Louvain-La-Neuve, Belgium), dissolved in an isotonic saline solution (0.9% NaCl), was injected intraperitoneally at a dose of 8 mg/kg in a

volume of 0.01 ml/g of body weight, the control treatment consisting of an equal volume of isotonic saline solution. The dose and route of administration were selected on the basis of our previous studies[27,28], these parameters being known to also induce rewarding-like effects in mice as measured by conditioned place preference.[33]

Behavioral test chambers

A battery of eight chambers, connected to a custom written software for data collection, was used to measure mice locomotor activity, one mouse being tested in each chamber. Each activity chamber was constituted of a removable transparent polycarbonate tub (22 x 12 cm surface x 12 cm height), embedded onto a black-paint wooden plank serving as a stable base. The lid was made of a transparent perforated acryl-glass tablet. Two photocell sources and detectors were mounted on the plank such that infrared light-beams were located on the two long sides of the tub at 2-cm heights from the floor, 8-cm apart and spaced 6.5 cm from each end of the tub. Locomotor activity was measured in terms of crossings detected by the beams, one crossing count being recorded every time an ambulating mouse broke successively the two parallel beams. The activity chambers were individually encased in sound-attenuated shells that were artificially ventilated and illuminated by a white light bulb during testing. Each shell door comprised a one-way window allowing periodic surveillance during testing.

[^{18}F]fallypride radiosynthesis

The radiotracer [^{18}F]fallypride was synthetized according to a method previously reported by Brichard *et al.* with slight modifications.[34] Briefly, the no-carrier-added synthesis of [^{18}F]fallypride was conducted by nucleophilic substitution with [^{18}F]fluoride of the p-toluenesulfonyl group of the commercially available precursor (ABX, Advanced Biochemical Compounds, Radeberg, Germany). After the labelling reaction that was conducted in

For Review Only

acetonitrile (1 mL) with 3.5 mg of the substrate at 120 °C for 5 min, the crude reaction mixture was diluted with water (6 mL) and the resulting solution injected on a semi-preparative HPLC column. The purification was carried out at 254 nm using a Phenomenex Luna C18 column (5 µm, 250 × 15 mm) at a flow rate of 7 mL/min with an isocratic eluent of water/acetonitrile/trietylamine (45:55:0.1%; retention time of 22 min). The subsequent formulation step (Lemaire et al., 1999) was realized by passing the HPLC collection solution, previously diluted with sodium chloride 0.9% (30 mL) and sodium ascorbate (30 mg) through a tC18 cartridge (360 mg, Waters). [^{18}F]fallypride was then eluted from the support with ethanol (1 mL) and diluted with an isotonic solution (6 mL) containing sodium ascorbate (10 mg) as stabilizer. Based on the starting activity recovered from the cyclotron (111 GBq), this process afforded batches of [^{18}F]fallypride ready for subsequent dilution for animal injection. The radiochemical yield was of 32 ± 5% (mean ± SD, decay corrected; n=24). At the end of beam, the averaged specific activity was 49 ± 20.4 Ci/µmol (1813.6 ± 755.6 GBq/µmol, decay corrected, n=24) and the synthesis duration of about 50 min. All the process was automated on a FASTlab synthesizer from GE Healthcare with single use components.

[^{18}F]fallypride microPET imaging data acquisition and processing

Twenty-four [^{18}F]fallypride microPET imaging sessions were completed and all necessary efforts were made to systematically repeat the same procedure. Anesthesia was induced with 4% of isoflurane, afterward the mice were placed prone in a dedicated bed. Anesthesia was maintained with 1–2% isoflurane in a mixture of air and oxygen (30%) at 0.6 l/min. A stereotaxic holder (Minerve, Esternay, France) was systematically used to reduce head movements. Respiratory rate and rectal temperature were permanently measured using a physiological monitoring system (Minerve, Esternay, France). Temperature was maintained at 37 ± 0.5° C, using an air warming system.

For Review Only

[^{18}F]fallypride was administered as bolus intravenous injection in the lateral tail vein over 20 seconds with a mean injected activity of 12.4 ± 3 MBq (range: 4.9 – 19.1 MBq). The mean injected mass of fallypride was 0.29 ± 0.35 µg (range: 0.02 – 2.51 µg). At the time of injection, dynamic microPET scans over 60 minutes were acquired in list-mode using a Siemens Concorde Focus 120 microPET (Siemens, Munich, Germany) and followed by 10 minutes transmission measurement with ^{57}Co point source. The list-mode emission data were histogrammed into three-dimensional (3D) sinograms by Fourier rebinning and reconstructed by filtered backprojection with a ramp filter cutoff at the Nyquist frequency. All Corrections were applied except for scatter events (Bahri et al., 2009). No partial volume correction was performed on the acquired data. A set of 3D images was reconstructed in a 256 x 256 x 95 matrix and a zoom factor of 2. The reconstructed voxel size was 0.4 x 0.4 x 0.8 mm^3. The dynamic time framing was set as follows: 6 x 5 s, 6 x 10 s, 3 x 20 s, 5 x 30 s, 5 x 60 s, 8 x 150 s, 6 x 300 s, and all data were decay corrected to the beginning of each individual frame.

Immediately after PET acquisition, the anesthetized mice were transferred into a 9.4 Tesla MRI DirectDrive VNMRS horizontal bore system with a shielded gradient system (Agilent Technologies, Palo Alto, CA, USA). A 72-mm inner diameter volumetric coil and a 2-channels head surface coil (Rapid Biomedical GmbH, Würzurg, Germany) were used as transmitter and receiver coils, respectively. The 3D anatomical T2-weighted brain images were acquired with a fast spin echo multislice sequence using the following parameters: TR/TEeff = 2500/40 ms, matrix = 128 x 128 x 64, FOV = 20 x 20 x 10.5 mm^3, voxel size: 0.156 x 0.156 x 0.164 mm^3, and a total acquisition time of 21 min.

Imaging data were processed with PMOD software (version 3.7, PMOD Technologies Ltd., Zurich, Switzerland). The processing includes a manual rigid co-registration of individual MRI images to its corresponding PET images, a spatial normalization of the co-registered MRI onto the PMOD MRI template, and the extraction of the PET time-activity curves of the left and

For Review Only

right striatum as well as the cerebellum. Briefly, the inverse deformations parameters obtained during the spatial normalization of the individual MRI images onto the PMOD template were used to bring the mouse brain atlas into the native dynamic PET space and then extract the TACs based on the atlas predefined structures.[37,38] The extracted TACs were then transferred into the kinetic modelling module of PMOD in order to estimate the [^{18}F]fallypride binding. The non-displaceable binding potential (BP_{ND}) parameter was calculated using the multi-linear reference tissue model (MRTM2) with the cerebellum as reference tissue.[39,40] We controlled the homogeneity of the TACs in the reference region (i.e. cerebellum) between each group, to rule out any bias from radiotracer inputs variations in the reference region. The statistical analysis revealed no difference between the four groups of the study for the cerebellum [^{18}F]fallypride TACs expressed as area under the curve (Supp. S1).

Experimental design and procedure

Experimental timeline and design are presented in Fig. 2. Ninety-six mice were housed in exercised (n=48) or sedentary (n=48) conditions from 28 days of age and kept in these conditions until the end of behavioral experimentation. Since mice from the two housing environments received cocaine or saline during testing, a basic 2 (housing conditions: EX vs SED) x 2 (pharmacological treatment: COC vs SAL) factorial design was generated with N=96, n=24 per group based on preliminary results indicating increasing and decreasing effects of cocaine (vs saline, η^2p = 0.162) and exercise (vs sedentary, η^2p = 0.093) respectively on [^{18}F]fallypride BP_{ND}. Note that experimental procedures and parameters associated with psychopharmacological tests were similar to those used in Lespine and Tirelli[28] where continuously exercised females C57BL/6J were found to be less vulnerable than their sedentary counterparts to acute and sensitized locomotor responsiveness to cocaine.

For Review Only

Testing included the following phases. (1) A pre-test habituation session to familiarize animals to novelty of the test context without neither injection nor measurements. (2) A 2nd session evaluating baseline locomotor activity under saline given to all animals. (3) Nine once-daily injections of cocaine or saline, with the measurement of hyperlocomotor effect of cocaine after each injection, initiating locomotor sensitization after the baseline session. (4) Taking place 21-23 days after the last cocaine injection, a session assessing expression of sensitization on which animals received their previous respective pharmacological treatment. Throughout psychopharmacological testing, mice were weighed and received their pharmacological treatment right before being placed in the test chamber, recording of locomotion lasting 30 min in all sessions. Experimental blinding was not realized because the unique experimenter inevitably knew the housing condition and the pharmacological treatment of each mouse. (5) Twenty-four hours after the test of expression of sensitization, mice underwent [^{18}F]fallypride microPET scan. Note that the wheels (for exercised mice) were removed 24h before neuroimaging measurement to avoid any potential effect of overnight wheel-running exercise on neuro-functional measures.

Due to practical reasons, the whole experiment was organized into twelve lots purchased and tested successively (each lot consisting of 8 mice). In each lot, two mice were assigned to one of the four experimental group by means of a computer-generated randomization schedule, the eight mice housed in acclimation cages contributing to the four possible groups (sedentary/cocaine, sedentary/saline, exercised/cocaine, and exercised/saline). Therefore, the four groups were systematically represented within each lot by 2 mice to consider any between-lot variability as well as that due to time and circumstances of testing (i.e. randomized block design, Supp. S2). Additionally, due to impracticality to test 8 mice in a row on the same micro-PET scanning session, each block (n=8) was further split into 2 blocks (n=4) for the test for expression of sensitization and microPET imaging procedures. Again, the four groups were

For Review Only

systematically represented within each block by one mouse (Supp. S2). Therefore, mice were tested for expression of sensitization either 21 (half) or 23 (other half) days after the last cocaine injection, while all mice underwent neuroimaging scan 24h after this test. Note that the order of neuroimaging session was counterbalanced across subjects to avoid potential bias due to the specific activity variations. Experimenters conducting neuroimaging testing and analysis were blinded to experimental groups.

Statistical analysis

Inferential statistics were computed on the following data. (1) Acute responsiveness to locomotor-activating effects of cocaine, scored as the difference between values derived from the first cocaine session and those of the baseline session. (2) Overall responsiveness to locomotor-activating effects of cocaine over the initiation of sensitization (9 sessions) scored as the area under the curve with respect to zero (AUC ground; calculation formula are based on and detailed by Pruessner and coll.[41]). (3) Locomotor activity exhibited during the expression of sensitization. (4) The bilateral [^{18}F]fallypride BP_{ND} from left and right striatum of each subject were averaged to give a single [^{18}F]fallypride BP_{ND} value per subject.

Each set of data was treated according to a randomized block design with a fixed-model 2 x 2 ANOVA incorporating the housing condition (EX or SED; 2 levels) and pharmacological treatment (COC or SAL; 2 levels) as between-group factors, and with the lot as a blocking factor (with 12 or 24 levels for the behavioral and neuro-functional measures respectively, see Supp. S1). This was followed by planned crossed or simple contrasts[42]. Each contrast was derived from the mean-square error term (MSE) provided by the ANOVA. Based on previous experiments or preliminary results, exercised mice were expected to display (1) lower cocaine locomotor responsiveness than sedentary mice (crossed contrasts) and (2) lower values of BP_{ND} (simple contrast). Cocaine-receiving mice were expected to show (1) greater locomotor activity

For Review Only

and (2) higher BP_{ND} values than control saline mice (simple contrasts). Correlations were also computed to determine whether the amount of wheel-running displayed before testing was associated with cocaine behavioral or neuro-functional outcomes, and whether behavioral cocaine outcomes were associated with neuro-functional measures. Nocturnal distances over the 42-day pre-testing period were averaged for each (exercised) mouse, the resulting individual value serving as the measure of the overall distance travelled on the wheel. Effect sizes were given by η^2p, Pearson correlation coefficient r, and probability of superiority (PS) where appropriate.[43] Statistical significance threshold was set at 0.05.

DATA AVAILIBILITY

OPTION 1: The authors confirm that the data supporting the findings of this study are available within the supplementary material.

RESULTS

Psychopharmacological measures

Figure 3 Panel A depicts results dealing with baseline locomotor activity and initiation of cocaine locomotor sensitization over 9 once-daily sessions Panel B presents scores of acute responsiveness. Cocaine locomotor effect (vs saline, i.e., hyperlocomotor effect) was strong in each group ($ts_{(81)}$ = 3.85 and 7.39 with a probability of superiority (PS) of 72 % and 88 % in exercised and sedentary groups, respectively). However, this effect was significantly lower in exercised mice (planned contrasts: $\eta^2p = 0.073$, $t_{(81)} = 2.51$, $p = .007$). Panel C depicts overall responsiveness during the initiation of sensitization (AUC ground). The pattern of results was comparable to that found for acute responsiveness. While cocaine effect was strong in each group ($ts_{(81)}$ = 5.47 and 11.07 with PS of 80 % and 96 % in exercised and sedentary groups, respectively), it was much lower in exercised mice (planned contrasts: $\eta^2p = 0.162$, $t_{(81)} = 3.96$,

For Review Only

p < .001). Figure 4 presents locomotor activity on the last (9th) session of sensitization (panel A, descriptive statistics) and on the test for expression of sensitization (panels B and C). Consistent with previous experimental stages, long-term expression of the sensitized locomotor responsiveness was largely reduced in exercised mice (planned contrasts: $\eta^2p = 0.262$, $t_{(81)} = 5.37$, $p < .001$). Again, cocaine effect was unambiguous in each group ($ts_{(81)} = 5.48$ and 13.08 with PS of 80 % and 98 % in exercised and sedentary groups, respectively). Table 1 reports relationships between amounts of exercise and behavioral outcomes, and [^{18}F]fallypride BP_{ND}. Wheel-running exercise was strongly and positively associated with AUC ground in cocaine-receiving mice. However, this result should be cautiously interpreted in the context of other correlations results, number of tests performed, and sample size. Importantly, AUC ground was strongly and positively associated with expression of sensitization. The fact that wheel-running distances were strongly associated with AUC ground yet correlated weakly to expression of sensitization questions the nature of relationships reported between exercise and AUC ground (i.e., risk of false-positive).

[^{18}F]fallypride microPET neuroimaging

Figure 5 panel A displays representative [^{18}F]fallypride BP_{ND} images of mice of the four groups. Panel B presents [^{18}F]fallypride BP_{ND} measured in the striatum 24h after the expression of sensitization in exercised (EX/COC and EX/SAL) and sedentary (SED/COC and SED/SAL) mice. Due to technical problems (fails in proper intravenous radiotracer delivery at injection time), data from 23 mice (over 96) were not acquired or useable (EX/COC: n=5; EX/SAL: n=8; SED/COC: n=5; and SED/SAL: n=5). Panels C and D present the marginal means associated with main effects of housing conditions (EX: n=35; SED: n=38) and pharmacological treatment (COC: n=38; SAL: n=35) respectively. We found evidence for a moderate attenuating effect of aerobic exercise on [^{18}F]fallypride BP_{ND} ($\eta^2p = 0.075$, PS = 65, $t_{(50)} = 2.01$, $p = .024$).

https://mc.manuscriptcentral.com/braincom

For Review Only

Additionally, cocaine-receiving mice exhibited higher [^{18}F]fallypride BP_{ND} in striatum than their saline counterparts as supported by a large effect of pharmacological treatment (η^2p = 0.170, PS = 74 , $t_{(50)} = 3.20$, $p = .001$). However, crossed contrasts indicated that the interaction between housing conditions and the pharmacological treatment was clearly negligible ($\eta^2p < 0.005$, $t_{(50)} = 0.20$, $p = .42$). Table 2 reports relationships between behavioral outcomes and [^{18}F]fallypride BP_{ND}. There was no evidence for association between behavioral outcomes and BP_{ND} values.

Table 1. Relationships between averaged pre-testing running distances and behavioral and neuro-imaging outcomes (Pearson's coefficient (*p*-value)).

	Acute Responsiveness	AUC ground	Expression of Sensitization	[^{18}F]fallypride BP_{ND}
COC	-0.17 (.43)	0.43 (.035)	0.15 (.50)	-0.24 (.33)[a]
SAL	-0.11 (.61)	-0.10 (.66)	-0.03 (.89)	-0.09 (.75)[b]

[a] n=19, [b] n=16, n=24 otherwise.

Table 2. Relationships between behavioral outcomes and neuro-imaging outcomes expressed as [^{18}F]fallypride BP_{ND} (Pearson's coefficients (*p*-value)).

	EX/COC[a]	EX/SAL[b]	SED/COC[a]	SED/SAL[a]
Acute Responsiveness	-0.28 (.25)	-0.26 (.34)	0.05 (.85)	-0.28 (.25)
AUC ground	-0.17 (.48)	0.17 (.52)	0.28 (.24)	0.37 (.12)
Expression of Sensitization	0.20 (.41)	0.06 (.81)	0.005 (.98)	0.27 (.27)

[a] n=19, [b] n=16.

For Review Only

DISCUSSION

The main findings of the present study can be summarized as follows. (1) Previous results obtained in our laboratory were reproduced by showing that wheel-running exercise induced preventive effects against acute and chronic locomotor responsiveness to a rewarding dose of cocaine (8 mg/kg) in female C57BL/6J mice.[11,27] (2) The cocaine-sensitized brain, at the time of long-term expression, revealed a striatal increase in dopamine D2/3 receptors availability measured by [^{18}F]fallypride microPET. (3) Wheel-running was associated with attenuated dopamine D2/3 receptors availability measured by [^{18}F]fallypride microPET.

Our behavioral results are consistent with previously reported findings showing that male Wistar rats continuously housed with a wheel expressed little or no sensitized locomotor activity 15 days after 5 once-daily injections of 10 mg/kg cocaine.[10] Another study showed that continuous wheel-running exercise was effective at reducing the locomotor-activating effects of 3 and 10 mg/kg cocaine in Long-Evans females.[44] Such attenuating effect was also observed in C57BL/6J mice housed in large home-cages comprising a running wheel as part of a composite housing environment made of inanimate objects and conspecifics.[45,46] More generally, our results add to extensive preclinical literature reporting preventive consequences of wheel-running exercise on behavioral markers of sensitivity to addictive properties of drugs of abuse.[2]

Physical exercise is known to act on DA system and to possess rewarding properties, like cocaine and other drugs of abuse.[47,48] For instance, Greenwood and co-workers[48] reported that Fischer344 rats exercising with a running wheel for 6 weeks preferred a compartment paired with the aftereffect of exercise. They also showed that a 6-week continuous access to wheels resulted in increases in ΔFosB/FosB immunoreactivity in the nucleus accumbens (Acb), tyrosine hydroxylase (TH) mRNA levels in the ventral tegmental area (VTA) and delta opioid

For Review Only

receptor mRNA levels in the Acb shell, whereas dopamine receptor D2 mRNA is reduced in the Acb core. It is thus tempting to ascribe the exercise-induced protective effects against the cocaine-induced locomotor sensitization to its neuroplastic effects on dopaminergic neurotransmission. To our knowledge, our study is the first to report in vivo PET imaging of mouse striatum D2/3R under aerobic exercise in the context of cocaine sensitization. First, we observed that long-term expression of cocaine sensitization is associated with an increase of D2/3R availability in the mouse striatum. Those results are in line with studies suggesting that psychomotor sensitization to stimulant is linked to enhanced D2/3R availability which may explain the high locomotor response of psychostimulant sensitized mice to direct-acting D2 agonists.[20,21,49,50]. Although previous reports hypothesized that the behavioral sensitization could occur through an increase of the dopamine D2 high affinity state receptors availability[25,51,52], the [^{18}F]fallypride radiotracer is not able to discriminate between the two affinity states of D2 receptors. Therefore, our results must not rely on the proportion of high versus low affinity state but rather on the total amount of available receptors. Our work could be interpreted in light of the supposed enhanced activity of DA neurons in the VTA during cocaine sensitization where the increased D2/3R availability in the striatum may contribute to the enhanced locomotor response induced by repeated cocaine injections.[53] Vanderschuren and Kalivas[54] well reviewed the adaptations of DA transmission associated with the long-term expression of sensitization. They highlighted that cocaine sensitized synapses showed increased release of dopamine and increased sensitivity of DA receptors. In our protocol, imaging sessions took place 24 hours after the last exposure to cocaine, we suggest that our results are related to an increased D2/3R density rather than a decrease in synaptic DA.

Second, we observed a decreased D2/3R availability in the striatum of exercised mice compared to their sedentary counterparts. This is in accordance with previous results showing that wheel-

For Review Only

running induced a reduction of D2R mRNA in Fisher 344 rats Acb core.[48] The authors also reported an increased TH mRNA level in the VTA leading them to propose that voluntary exercise can increase the synthetic capacity of DA in the striatum.[48,55] These modifications could be related to neuroplastic events induced by an enhanced expression of ΔFosB transcription factor in the Acb nucleus.[48] Interestingly, a sustained accumulation of ΔFosB in the Acb nucleus was also described following chronic cocaine exposure, as well as for others drugs of abuse.[56–58] This common feature led to the assumption that neuroplasticity induced by voluntary exercise could alter DA neurotransmission in the mesolimbic reward pathway which may contribute then to the beneficial effects of exercise on cocaine sensitization.[48] This is supported by data reporting that chronically exercised Sprague Dawley rats displayed a lower DA release, and a hampered DA reuptake, under amphetamine challenge.[59] Physical exercise has been shown to reduce the basal level of DA in the rat striatum, which accord to the hypothesis of a reduced DA tone resulting from chronic physical exercise despite an enhanced synthetic capacity.[60] Besides, our current results are coherent with the study of Fisher and coll. reporting a reduced dopamine D2 mRNA expression as a result of exercise in basal ganglia of C57Bl6 mice.[61] We suggest that voluntary physical exercise acts on DA mesolimbic reward pathway partly through a reduced D2/3R availability in the mouse striatum, which trait makes exercised mice more resilient to psychomotor effects of cocaine. However, our current data do not allow us to unravel the mechanism of decreased D2/3R availability which can be related to a decreased D2/3R density or an increase in endogenous synaptic DA.

Some limitations warrant mention. First, the [^{18}]Fallypride radiotracer displays a nearly equal affinity for D2 and D3 dopamine receptors in vivo[62], hence the [^{18}]Fallypride BP_{ND} parameter primarily reflect a combination of signals from D2 and D3 receptors. It has been suggested that up to 20% of the [^{18}]Fallypride binding may be due to D3R in vivo.[62] This is of relevance

For Review Only

considering that D3R are highly expressed in the mesolimbic DA system and involved in the pathophysiology of addiction.[63] Strikingly, their expression seemed to be increased in nicotine behavioral sensitization.[64] Determining the respective proportion of D2R and D3R in our present results would definitely need further investigations. The second technical limitation relies on the spatial resolution of the microPET scanner, which is about 1.5 mm^2 in our setup.[36] This has of course to be taken into account when attempting to achieve in vivo molecular imaging of mouse brain. The mouse brain atlas implemented in Pmod 3.7, derived from the work of Mirrione and coll.[36], do not allow to analyze separately the ventral and the dorsal parts of the striatum, the Accumbens (Acb) and the Caudate Putamen (CPu) respectively. As a consequence, the ROI analyzed to compute the [^{18}F]fallypride BP_{ND} is constituted by the entire striatum. This is of interest as the DA inputs within the Acb hail from the VTA whereas the DA afferences throughout the CPu originate from the Substancia Nigra (SN). On one hand, DA neurons of the VTA are known to play a major role into the reward and motivation processes[65], and to their pathological counterpart that is addiction. On the other hand, DA neurons lying in the SN are controlling motor functions. Intuitively, one would think that the [^{18}F]fallypride BP_{ND} modifications described here are primarily located in the Acb. However, D2R are involved in locomotor activity and may be implicated in [^{18}F]fallypride BP_{ND} variations induced by physical exercise.[66] Our results may be conflicting with those of Vucckovic and coll.[66], reporting an increased [^{18}F]fallypride BP_{ND} in a mouse model relevant for the study of Parkinson's disease under exercise condition. However, the pathophysiological paradigm shift prevents any direct comparison of the data as it has been revealed that exercise have a differential effect on the dopaminergic system in DA terminals depleted mice compare to control mice.[61,67] One of the main limitations of our study relies on the use of female only. A single-sex experiment in male C57BL/6J mice did not find any evidence for an effect of (treadmill) exercise on [^{18}F]fallypride BP_{ND} within the basal ganglia.[66] Lynch and coll.

For Review Only

reviewed evidence for the sex-specific differences in the efficacy of exercise against addiction disorders and highlighted that females have an enhanced sensitivity to this protective effect.[30] Clinical assessment of D2/3R in methamphetamine users under behavioral intervention with exercise training has been achieved using [^{18}F]fallypride PET imaging.[68] Interestingly, they reported an increased [^{18}F]fallypride BP_{ND} in the whole striatum of exercised patients compared to those of the control group (*i.e.* methamphetamine users under behavioral intervention with educational training). Thus, the authors suggest that under depleted striatal D2/3R availability, as shown by the methamphetamine users, physical exercise may increase the availability of these receptors while pointing out the relatively small sample size. Besides, the absence of healthy control in the study design precludes conclusions drawing on the effect of exercise on striatal D2/3R availability in physiological conditions. Moreover, our design focused on the sensitization process (and the associated long-term expression) which is thought to be a useful model to investigate early events in the natural history of addiction pathophysiology (e.g. recreational use). On the contrary, the work of Robertson and coll.[68] examined exercise intervention in patients with an extensive drug history that has been shown to be associated with a decrease in D2/3R availability. Furthermore, patients were under complete abstinence which could impact DA neurochemical processes.

In conclusion, we report a replication study of a protective effect of wheel-running exercise on both initiation and expression of cocaine locomotor sensitization in female C57BL/6J mice. We provided data as proof-of-concept in female C57BL/6J, showing that exercise-induced neuroplasticity within mesolimbic DA pathway includes a reduced D2/3R availability in the striatum, while cocaine locomotor sensitization is associated with an increased D2/3R availability in this brain area, and contributing to the neurobiological understanding of physical exercise and its positive impact on addictive behaviors. Further investigations are warranted to

For Review Only

unravel the molecular mechanisms by which exercise affects dopaminergic signaling and to characterize sex-related differences in aerobic exercise induced plasticity and its effects.

ACKNOWLEDGMENTS

None

FUNDING

The present work was supported by grants FRSM 3.4590.12 from the “Fonds National de la Recherche Scientifique” (FNRS) and the University of Liège “Fonds Spéciaux pour la Recherche” obtained by Ezio TIRELLI and Alain PLENEVAUX.

For Review Only

CONFLICTS OF INTEREST

The authors declare no competing financial interests.

REFERENCES

1. De La Garza R, Yoon JH, Thompson-Lake DGY, et al. Treadmill exercise improves fitness and reduces craving and use of cocaine in individuals with concurrent cocaine and tobacco-use disorder. *Psychiatry Res*. 2016;245:133-140. doi:10.1016/j.psychres.2016.08.003
2. Lynch WJ, Peterson AB, Sanchez V, Abel J, Smith MA. Exercise as a novel treatment for drug addiction: A neurobiological and stage-dependent hypothesis. *Neurosci Biobehav Rev*. 2013;37(8):1622-1644. doi:10.1016/j.neubiorev.2013.06.011

3. Lisha NE, Sussman S. Relationship of high school and college sports participation with alcohol, tobacco, and illicit drug use: A review. *Addict Behav*. 2010;35(5):399-407. doi:10.1016/j.addbeh.2009.12.032
4. Smith MA, Schmidt KT, Iordanou JC, Mustroph ML. Aerobic exercise decreases the positive-reinforcing effects of cocaine. *Drug Alcohol Depend*. 2008;98(1-2):129-135. doi:10.1016/j.drugalcdep.2008.05.006
5. Smith MA, Walker KL, Cole KT, Lang KC. The effects of aerobic exercise on cocaine self-administration in male and female rats. *Psychopharmacology (Berl)*. 2011;218(2):357-369. doi:10.1007/s00213-011-2321-5
6. Smith MA, Pitts EG. Access to a running wheel inhibits the acquisition of cocaine self-administration. *Pharmacol Biochem Behav*. 2011;100(2):237-243. doi:10.1016/j.pbb.2011.08.025
7. Smith MA, Pitts EG. Wheel running decreases the positive reinforcing effects of heroin. *Pharmacol Reports*. 2012;64(4):960-964. doi:10.1016/S1734-1140(12)70891-5
8. Engelmann AJ, Aparicio MB, Kim A, et al. Chronic wheel running reduces maladaptive patterns of methamphetamine intake: regulation by attenuation of methamphetamine-induced neuronal nitric oxide synthase. *Brain Struct Funct*. 2014;219(2):657-672. doi:10.1007/s00429-013-0525-7
9. Lacy RT, Strickland JC, Brophy MK, Witte MA, Smith MA. Exercise decreases speedball self-administration. *Life Sci*. 2014;114(2):86-92. doi:10.1016/j.lfs.2014.08.005
10. Renteria Diaz L, Siontas D, Mendoza J, Arvanitogiannis A. High levels of wheel running protect against behavioral sensitization to cocaine. *Behav Brain Res*. 2013;237:82-85. doi:10.1016/j.bbr.2012.09.014
11. Geuzaine A, Tirelli E. Wheel-running mitigates psychomotor sensitization initiation

For Review Only

but not post-sensitization conditioned activity and conditioned place preference induced by cocaine in mice. *Behav Brain Res*. 2014;262:57-67. doi:10.1016/j.bbr.2014.01.002

12. Robinson TE, Berridge KC. Addiction. *Annu Rev Psychol*. 2003;54(1):25-53. doi:10.1146/annurev.psych.54.101601.145237
13. Anderson S., Pierce RC. Cocaine-induced alterations in dopamine receptor signaling: Implications for reinforcement and reinstatement. *Pharmacol Ther*. 2005;106(3):389-403. doi:10.1016/j.pharmthera.2004.12.004
14. Wang GJ, Smith L, Volkow ND, et al. Decreased dopamine activity predicts relapse in methamphetamine abusers. *Mol Psychiatry*. 2012;17(9):918-925. doi:10.1038/mp.2011.86
15. Volkow ND, Fowler JS, Wolf AP, et al. Effects of chronic cocaine abuse on postsynaptic dopamine receptors. *Am J Psychiatry*. 1990;147(6):719-724. doi:10.1176/ajp.147.6.719
16. Volkow ND. Is Methylphenidate Like Cocaine? *Arch Gen Psychiatry*. 1995;52(6):456. doi:10.1001/archpsyc.1995.03950180042006
17. Volkow ND, Fowler JS, Wang G-J, Swanson JM. Dopamine in drug abuse and addiction: results from imaging studies and treatment implications. *Mol Psychiatry*. 2004;9(6):557-569. doi:10.1038/sj.mp.4001507
18. Nader J, Claudia C, Rawas R El, et al. Loss of Environmental Enrichment Increases Vulnerability to Cocaine Addiction. *Neuropsychopharmacology*. 2012;37(7):1579-1587. doi:10.1038/npp.2012.2
19. Nader MA, Czoty PW. PET Imaging of Dopamine D2 Receptors in Monkey Models of Cocaine Abuse: Genetic Predisposition Versus Environmental Modulation. *Am J Psychiatry*. 2005;162(8):1473-1482. doi:10.1176/appi.ajp.162.8.1473

For Review Only

20. Peris J, Boyson SJ, Cass WA, et al. Persistence of neurochemical changes in dopamine systems after repeated cocaine administration. *J Pharmacol Exp Ther*. 1990;253(1):38-44. http://www.ncbi.nlm.nih.gov/entrez/query.fcgi?cmd=Retrieve&db=PubMed&dopt=Citation&list_uids=2329520

21. Sousa FCF, Gomes PB, Mace^do DS, Marinho MMF, Viana GSB. Early Withdrawal From Repeated Cocaine Administration Upregulates Muscarinic and Dopaminergic D2-Like Receptors in Rat Neostriatum. *Pharmacol Biochem Behav*. 1999;62(1):15-20. doi:10.1016/S0091-3057(98)00142-7

22. Maggos C. Sustained Withdrawal Allows Normalization of In Vivo [11C]N-Methylspiperone Dopamine D2 Receptor Binding after Chronic Binge Cocaine A Positron Emission Tomography Study in Rats. *Neuropsychopharmacology*. 1998;19(2):146-153. doi:10.1016/S0893-133X(98)00009-8

23. Claye LH, Akunne HC, Duff Davis M, DeMattos S, Soliman KFA. Behavioral and neurochemical changes in the dopaminergic system after repeated cocaine administration. *Mol Neurobiol*. 1995;11(1-3):55-66. doi:10.1007/BF02740684

24. Stanwood GD, Lucki I, McGonigle P. Differential regulation of dopamine D2 and D3 receptors by chronic drug treatments. *J Pharmacol Exp Ther*. Published online 2000.

25. Briand LA, Flagel SB, Seeman P, Robinson TE. Cocaine self-administration produces a persistent increase in dopamine D2 High receptors. *Eur Neuropsychopharmacol*. 2008;18(8):551-556. doi:10.1016/j.euroneuro.2008.01.002

26. Seeman P, Ko F, Tallerico T. Dopamine receptor contribution to the action of PCP, LSD and ketamine psychotomimetics. *Mol Psychiatry*. 2005;10(9):877-883. doi:10.1038/sj.mp.4001682

27. Lespine LF, Tirelli E. The protective effects of free wheel-running against cocaine

For Review Only

psychomotor sensitization persist after exercise cessation in C57BL/6J mice. *Neuroscience*. 2015;310:650-664. doi:10.1016/j.neuroscience.2015.10.009

28. Lespine LF, Tirelli E. Evidence for a long-term protection of wheel-running exercise against cocaine psychomotor sensitization in adolescent but not in adult mice. *Behav Brain Res*. 2018;349(May):63-72. doi:10.1016/j.bbr.2018.04.054

29. Lespine LF, Plenevaux A, Tirelli E. Wheel-running exercise before and during gestation against acute and sensitized cocaine psychomotor-activation in offspring. *Behav Brain Res*. 2019;363(August 2018):53-60. doi:10.1016/j.bbr.2019.01.049

30. Lynch WJ, Robinson AM, Abel J, Smith MA. Exercise as a Prevention for Substance Use Disorder: a Review of Sex Differences and Neurobiological Mechanisms. *Curr Addict Reports*. 2017;4(4):455-466. doi:10.1007/s40429-017-0178-3

31. Zhou Y, Zhao M, Zhou C, Li R. Sex differences in drug addiction and response to exercise intervention: From human to animal studies. *Front Neuroendocrinol*. 2016;40:24-41. doi:10.1016/j.yfrne.2015.07.001

32. Kilkenny C, Browne WJ, Cuthill IC, Emerson M, Altman DG. Improving Bioscience Research Reporting: The ARRIVE Guidelines for Reporting Animal Research. *PLoS Biol*. 2010;8(6):e1000412. doi:10.1371/journal.pbio.1000412

33. Brabant C, Quertemont E, Tirelli E. Evidence that the relations between novelty-induced activity, locomotor stimulation and place preference induced by cocaine qualitatively depend upon the dose: a multiple regression analysis in inbred C57BL/6J mice. *Behav Brain Res*. 2005;158(2):201-210. doi:10.1016/j.bbr.2004.08.020

34. Brichard L, Ferrari V, Smith R, Aigbirhio FI. Synthesis of [18 F]-Fallypride. In: *Radiochemical Syntheses*. John Wiley & Sons, Inc.; 2012:95-102. doi:10.1002/9781118140345.ch11

35. Lemaire C, Plenevaux A, Aerts J, et al. Solid phase extraction—an alternative to the

For Review Only

use of rotary evaporators for solvent removal in the rapid formulation of PET radiopharmaceuticals. *J Label Compd Radiopharm*. 1999;42(1):63-75. doi:10.1002/(SICI)1099-1344(199901)42:1<63::AID-JLCR167>3.0.CO;2-R

36. Bahri MA, Plenevaux A, Warnock G, Luxen A, Seret A. NEMA NU4-2008 image quality performance report for the microPET focus 120 and for various transmission and reconstruction methods. *J Nucl Med*. 2009;50(10):1730-1738. doi:10.2967/jnumed.109.063974

37. Ma Y, Hof PR, Grant SC, et al. A three-dimensional digital atlas database of the adult C57BL/6J mouse brain by magnetic resonance microscopy. *Neuroscience*. 2005;135:1203-1215. doi:10.1016/j.neuroscience.2005.07.014

38. Mirrione MM, Schiffer WK, Fowler JS, Alexoff DL, Dewey SL, Tsirka SE. A novel approach for imaging brain–behavior relationships in mice reveals unexpected metabolic patterns during seizures in the absence of tissue plasminogen activator. *Neuroimage*. 2007;38(1):34-42. doi:10.1016/j.neuroimage.2007.06.032

39. Innis RB, Cunningham VJ, Delforge J, et al. Consensus Nomenclature for in vivo Imaging of Reversibly Binding Radioligands. *J Cereb Blood Flow Metab*. 2007;27(9):1533-1539. doi:10.1038/sj.jcbfm.9600493

40. Ichise M, Liow JS, Lu JQ, et al. Linearized reference tissue parametric imaging methods: Application to [11C]DASB positron emission tomography studies of the serotonin transporter in human brain. *J Cereb Blood Flow Metab*. 2003;23(9):1096-1112. doi:10.1097/01.WCB.0000085441.37552.CA

41. Pruessner JC, Kirschbaum C, Meinlschmid G, Hellhammer DH. Two formulas for computation of the area under the curve represent measures of total hormone concentration versus time-dependent change. *Psychoneuroendocrinology*. 2003;28(7):916-931. doi:10.1016/S0306-4530(02)00108-7

For Review Only

42. Rosnow RL, Rosenthal R. Effect Sizes. *Zeitschrift für Psychol / J Psychol*. 2009;217(1):6-14. doi:10.1027/0044-3409.217.1.6

43. Fritz CO, Morris PE, Richler JJ. Effect size estimates: Current use, calculations, and interpretation. *J Exp Psychol Gen*. 2012;141(1):2-18. doi:10.1037/a0024338

44. Smith MA, Witte MA. The effects of exercise on cocaine self-administration, food-maintained responding, and locomotor activity in female rats: Importance of the temporal relationship between physical activity and initial drug exposure. *Exp Clin Psychopharmacol*. Published online 2012. doi:10.1037/a0029724

45. Bezard E, Dovero S, Belin D, et al. Enriched Environment Confers Resistance to 1-Methyl-4-Phenyl-1,2,3,6-Tetrahydropyridine and Cocaine: Involvement of Dopamine Transporter and Trophic Factors. *J Neurosci*. 2003;23(35):10999-11007. doi:10.1523/JNEUROSCI.23-35-10999.2003

46. Solinas M, Thiriet N, Rawas R El, Lardeux V, Jaber M. Environmental Enrichment During Early Stages of Life Reduces the Behavioral, Neurochemical, and Molecular Effects of Cocaine. *Neuropsychopharmacology*. 2009;34(5):1102-1111. doi:10.1038/npp.2008.51

47. Belke TW. Running and responding reinforced by the opportunity to run: effect of reinforcer duration. *J Exp Anal Behav*. 1997;67(3):337-351. doi:10.1901/jeab.1997.67-337

48. Greenwood BN, Foley TE, Le T V., et al. Long-term voluntary wheel running is rewarding and produces plasticity in the mesolimbic reward pathway. *Behav Brain Res*. 2011;217(2):354-362. doi:10.1016/j.bbr.2010.11.005

49. Trulson ME, Ulissey MJ. Chronic cocaine administration decreases dopamine synthesis rate and increases [3H] spiroperidol binding in rat brain. *Brain Res Bull*. 1987;19(1):35-38. doi:10.1016/0361-9230(87)90162-6

For Review Only

50. De Vries T. Relapse to Cocaine- and Heroin-Seeking Behavior Mediated by Dopamine D2 Receptors Is Time-Dependent and Associated with Behavioral Sensitization. *Neuropsychopharmacology*. 2002;26(1):18-26. doi:10.1016/S0893-133X(01)00293-7

51. Seeman P, Tallerico T, Ko F, Tenn C, Kapur S. Amphetamine-sensitized animals show a marked increase in dopamine D2 high receptors occupied by endogenous dopamine, even in the absence of acute challenges. *Synapse*. 2002;46(4):235-239. doi:10.1002/syn.10139

52. Novak G, Seeman P, Foll B Le. Exposure to nicotine produces an increase in dopamine D2 High receptors: A possible mechanism for dopamine hypersensitivity. *Int J Neurosci*. 2010;120(11):691-697. doi:10.3109/00207454.2010.513462

53. Liu CL, Wang YK, Jin GZ, Shi WX, Gao M. Cocaine-induced locomotor sensitization associates with slow oscillatory firing of neurons in the ventral tegmental area. *Sci Rep*. 2018;8(1):1-11. doi:10.1038/s41598-018-21592-7

54. Vanderschuren LJMJ, Kalivas PW. Alterations in dopaminergic and glutamatergic transmission in the induction and expression of behavioral sensitization: a critical review of preclinical studies. *Psychopharmacology (Berl)*. 2000;151(2-3):99-120. doi:10.1007/s002130000493

55. Foley TE, Fleshner M. Neuroplasticity of Dopamine Circuits After Exercise: Implications for Central Fatigue. *NeuroMolecular Med*. 2008;10(2):67-80. doi:10.1007/s12017-008-8032-3

56. Kelz MB, Chen J, Carlezon Jr. W a, et al. Expression of the transcription factor deltaFosB in the brain controls sensitivity to cocaine. *Nature*. 1999;401(6750):272-276. doi:10.1038/45790

57. Merlo Pich E. Common Neural Substrates for the Addictive Properties of Nicotine and Cocaine. *Science (80-)*. 1997;275(5296):83-86. doi:10.1126/science.275.5296.83

For Review Only

58. Atkins JB, Chlan-Fourney J, Nye HE, Hiroi N, Carlezon WA, Nestler EJ. Region-specific induction of ?FosB by repeated administration of typical versus atypical antipsychotic drugs. *Synapse*. 1999;33(2):118-128. doi:10.1002/(SICI)1098-2396(199908)33:2<118::AID-SYN2>3.0.CO;2-L

59. Marques E, Vasconcelos F, Rolo MR, et al. Influence of Chronic Exercise on the Amphetamine-Induced Dopamine Release and Neurodegeneration in the Striatum of the Rat. *Ann N Y Acad Sci*. 2008;1139(1):222-231. doi:10.1196/annals.1432.041

60. Meeusen R, Smolders I, Sarre S, et al. Endurance training effects on neurotransmitter release in rat striatum: an in vivo microdialysis study. *Acta Physiol Scand*. 1997;159(4):335-341. doi:10.1046/j.1365-201X.1997.00118.x

61. Fisher BE, Petzinger GM, Nixon K, et al. Exercise-induced behavioral recovery and neuroplasticity in the 1-methyl-4-phenyl-1,2,3,6-tetrahydropyridine-lesioned mouse basal ganglia. *J Neurosci Res*. 2004;77(3):378-390. doi:10.1002/jnr.20162

62. Mukherjee J, Constantinescu CC, Hoang AT, Jerjian T, Majji D, Pan M-L. Dopamine D3 receptor binding of 18 F-fallypride: Evaluation using in vitro and in vivo PET imaging studies. *Synapse*. 2015;69(12):577-591. doi:10.1002/syn.21867

63. Volkow ND, Morales M. The Brain on Drugs: From Reward to Addiction. *Cell*. 2015;162(4):712-725. doi:10.1016/j.cell.2015.07.046

64. Le Foll B, Diaz J, Sokoloff P. Increased dopamine D3 receptor expression accompanying behavioral sensitization to nicotine in rats. *Synapse*. 2003;47(3):176-183. doi:10.1002/syn.10170

65. Salamone JD, Correa M. The Mysterious Motivational Functions of Mesolimbic Dopamine. *Neuron*. 2012;76(3):470-485. doi:10.1016/j.neuron.2012.10.021

66. Vučcković MG, Li Q, Fisher B, et al. Exercise elevates dopamine D2 receptor in a mouse model of Parkinson's disease: In vivo imaging with [18F]fallypride. *Mov*

For Review Only

Disord. 2010;25(16):2777-2784. doi:10.1002/mds.23407

67. Petzinger GM, Walsh JP, Akopian G, et al. Effects of treadmill exercise on dopaminergic transmission in the 1-methyl-4-phenyl-1,2,3,6-tetrahydropyridine-lesioned mouse model of basal ganglia injury. *J Neurosci*. 2007;27(20):5291-5300. doi:10.1523/JNEUROSCI.1069-07.2007

68. Robertson CL, Ishibashi K, Chudzynski J, et al. Effect of Exercise Training on Striatal Dopamine D2/D3 Receptors in Methamphetamine Users during Behavioral Treatment. *Neuropsychopharmacology*. 2015;41(1740-634X (Electronic)):1629-1636. doi:10.1038/npp.2015.331

69. Franklin K, Paxinos G. The Mouse Brain in Stereotaxic Coordinates. Elsevier; 2007.

For Review Only

Figure legend:

Figure 1. Wheel-running activity recorded prior to the testing period. Nocturnal (light off) and diurnal (light on) wheel-running activity of mice randomly assigned to exercise conditions. Since at this stage of the experiment mice from the cocaine (COC, n=24) and saline (SAL, n=24) groups were still undistinguishable, no inferential statistics were conducted on these data. All mice showed a rapid increase in wheel-running over the two first weeks until reaching a plateau. Bars represent 95% confidence intervals.

Figure 2. **Experimental timeline and design.** At 28 days of age, 96 mice were housed individually either in the presence (EX, n=48) or the absence (SED, n=48) of a running wheel. Testing began after 6 weeks in these housing conditions (from 28 to 70 days old). The experiment comprised four groups, mice from each housing group (EX or SED) receiving either cocaine or saline (with n=24 per group). Solid lines represent the presence of a running wheel

in the home-cage and dotted lines its absence. H: habituation session (to familiarize animals to the novelty of the test context without neither injection nor measures); B: baseline session; the 2nd once-daily session assessing the baseline activity under saline; C: cocaine intraperitoneal administration (9 once-daily sessions); S: control animals receiving saline intraperitoneal administration (9 once-daily sessions). E: session on which the expression of the sensitization was assessed 21-23 days after the last sensitizing injection, and under the previous pharmacological treatments. N: neuro-functional measures 24 h after the test of expression (microPET).

Figure 3. Acute responsiveness and initiation of sensitization. (A) Baseline locomotor activity (under saline) and initiation of locomotor sensitization over 9 once-daily sessions. (B) Acute responsiveness scored as the difference between values from the 1st and baseline sessions. (C) Overall locomotor responsiveness over the initiation of sensitization scored as AUC ground. *significant interaction-related difference between the cocaine effect observed in exercised mice (EX/COC, n=24 vs EX/SAL, n=24) and that measured in sedentary mice (SED/COC, n=24 vs SED/SAL, n=24) taken at a threshold of 0.05. Bars represent 95% confidence intervals.

Figure 4. Long-term expression of sensitization. (A) Locomotor responsiveness on the last (9th) once-daily session (descriptive statistics). (B) Locomotor responsiveness on the test for expression of sensitization. *significant housing conditions x pharmacological treatment interaction: cocaine effect measured in sedentary mice (SED/COC, n=24 vs. SED/SAL, n=24) is greater than that observed in exercised mice (EX/COC, n=24 vs EX/SAL, n=24). (C) Time-course of locomotor responsiveness during the test for expression of sensitization (descriptive, no inferential statistics were conducted on these data). Bars represent 95% confidence intervals.

For Review Only

Figure 5. Neuroimaging outcomes. (A) Left upper panel: coronal slice image of the mouse brain at Bregma 0.8 mm, based on the mouse Atlas of Franklin and Paxinos.[69] (A) Right upper panel: 3D depiction of regions of interest showing the Caudate Putamen (in blue) and the Nucleus Accumbens (in green). (A) Bottom: representative [^{18}F]fallypride BP_{ND} images of mice of the four groups, co-registered to their corresponding individual anatomical MRI. Note the difference in scales between the cocaine groups and the saline groups. (B) microPET-derived [^{18}F]Fallypride BP_{ND} measured 24 h after expression of sensitization in exercised and sedentary mice. (C) Marginal means associated with the effect of housing conditions. (D) Marginal means associated with the effect of pharmacological treatment. **C**s indicate significant difference compared to the corresponding control group. Bars represent 95% confidence intervals.

For Review Only

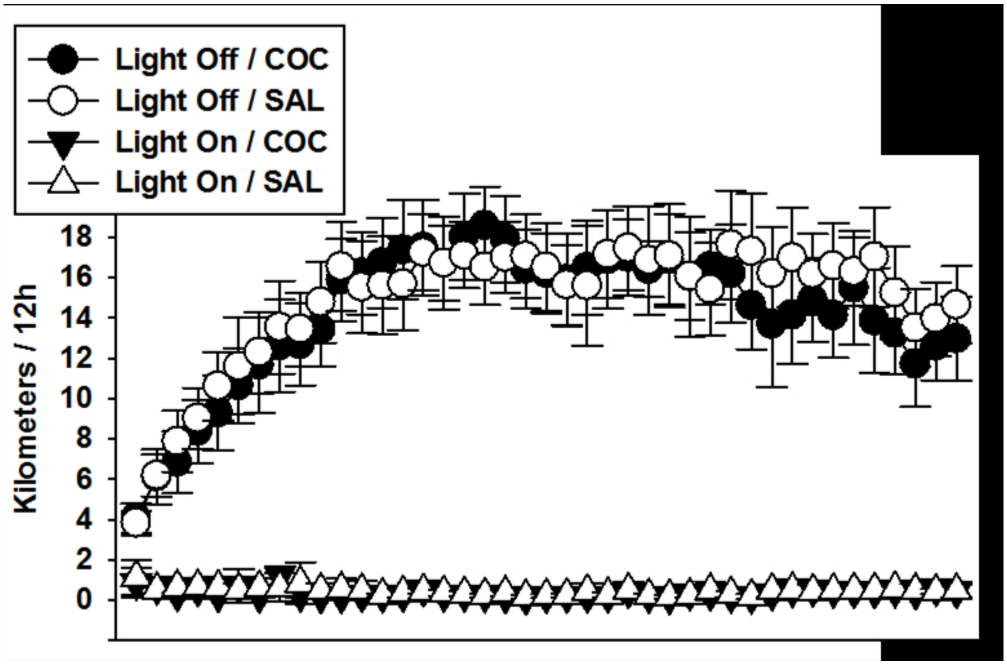

Figure 1. Wheel-running activity recorded prior to the testing period. Nocturnal (light off) and diurnal (light on) wheel-running activity of mice randomly assigned to exercise conditions. Since at this stage of the experiment mice from the cocaine (COC, n=24) and saline (SAL, n=24) groups were still undistinguishable, no inferential statistics were conducted on these data. All mice showed a rapid increase in wheel-running over the two first weeks until reaching a plateau. Bars represent 95% confidence intervals.

131x86mm (150 x 150 DPI)

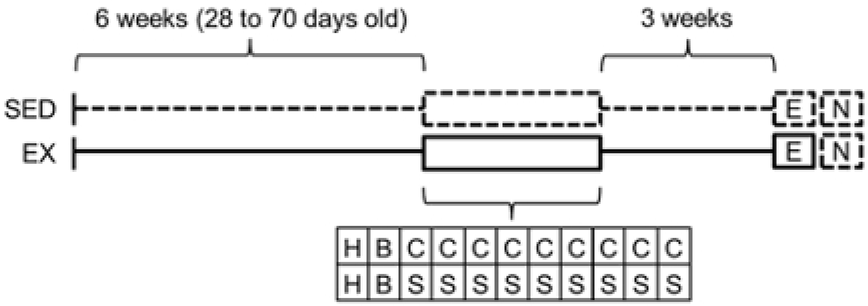

Figure 2. Experimental timeline and design. At 28 days of age, 96 mice were housed individually either in the presence (EX, n=48) or the absence (SED, n=48) of a running wheel. Testing began after 6 weeks in these housing conditions (from 28 to 70 days old). The experiment comprised four groups, mice from each housing group (EX or SED) receiving either cocaine or saline (with n=24 per group). Solid lines represent the presence of a running wheel in the home-cage and dotted lines its absence. H: habituation session (to familiarize animals to the novelty of the test context without neither injection nor measures); B: baseline session; the 2nd once-daily session assessing the baseline activity under saline; C: cocaine intraperitoneal administration (9 once-daily sessions); S: control animals receiving saline intraperitoneal administration (9 once-daily sessions). E: session on which the expression of the sensitization was assessed 21-23 days after the last sensitizing injection, and under the previous pharmacological treatments. N: neuro-functional measures 24 h after the test of expression (microPET).

152x56mm (72 x 72 DPI)

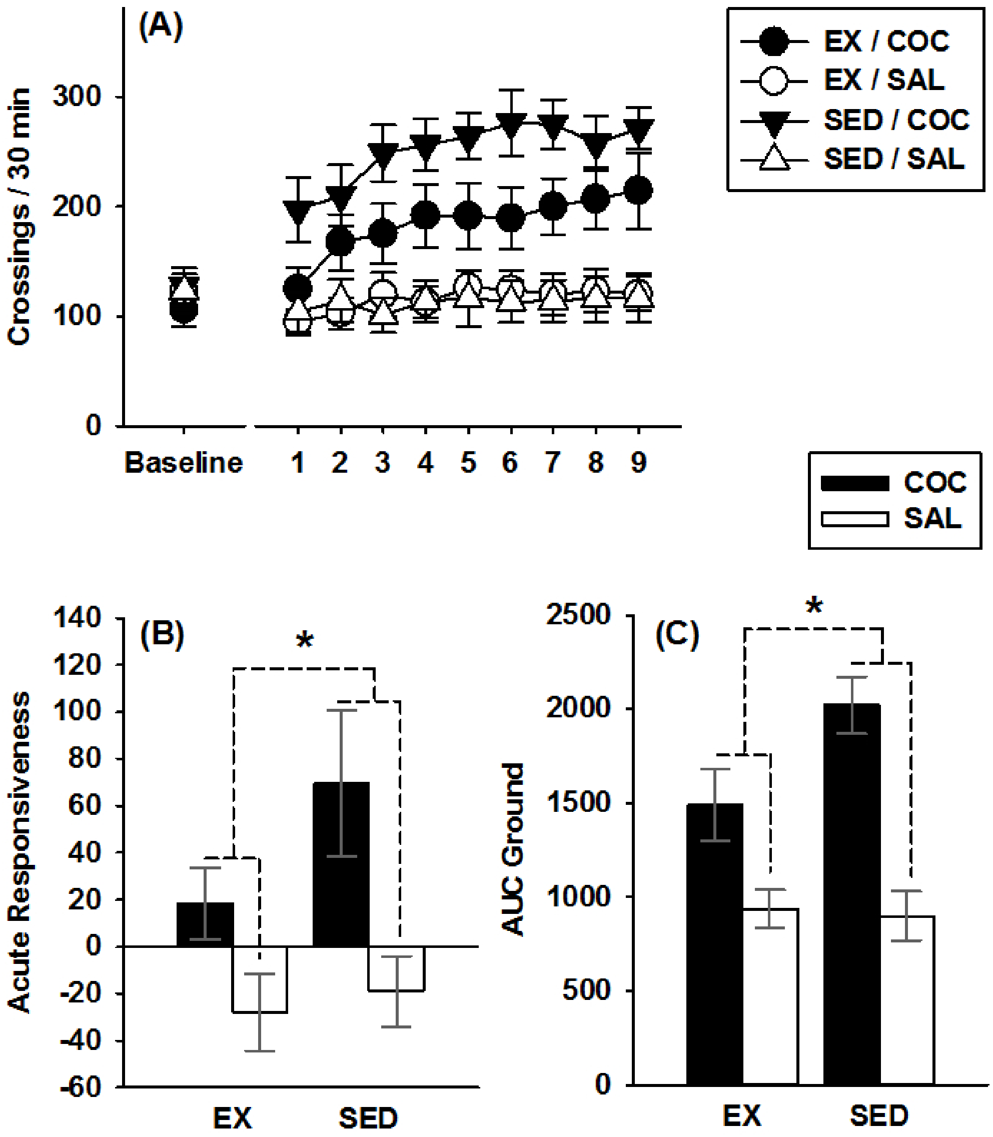

Figure 3. Acute responsiveness and initiation of sensitization. (A) Baseline locomotor activity (under saline) and initiation of locomotor sensitization over 9 once-daily sessions. (B) Acute responsiveness scored as the difference between values from the 1st and baseline sessions. (C) Overall locomotor responsiveness over the initiation of sensitization scored as AUC ground. *significant interaction-related difference between the cocaine effect observed in exercised mice (EX/COC, n=24 vs EX/SAL, n=24) and that measured in sedentary mice (SED/COC, n=24 vs SED/SAL, n=24) taken at a threshold of 0.05. Bars represent 95% confidence intervals.

118x136mm (150 x 150 DPI)

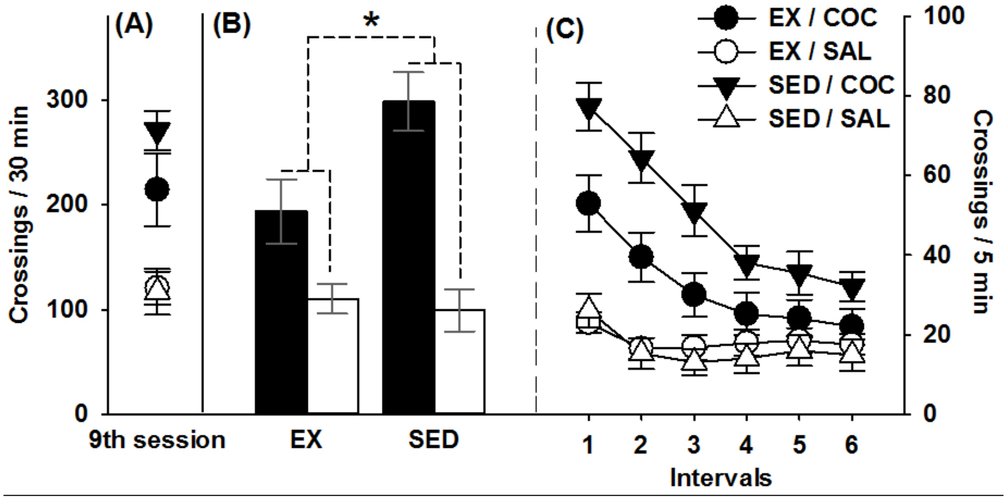

Figure 4. Long-term expression of sensitization. (A) Locomotor responsiveness on the last (9th) once-daily session (descriptive statistics). (B) Locomotor responsiveness on the test for expression of sensitization. *significant housing conditions x pharmacological treatment interaction: cocaine effect measured in sedentary mice (SED/COC, n=24 vs. SED/SAL, n=24) is greater than that observed in exercised mice (EX/COC, n=24 vs EX/SAL, n=24). (C) Time-course of locomotor responsiveness during the test for expression of sensitization (descriptive, no inferential statistics were conducted on these data). Bars represent 95% confidence intervals.

155x79mm (150 x 150 DPI)

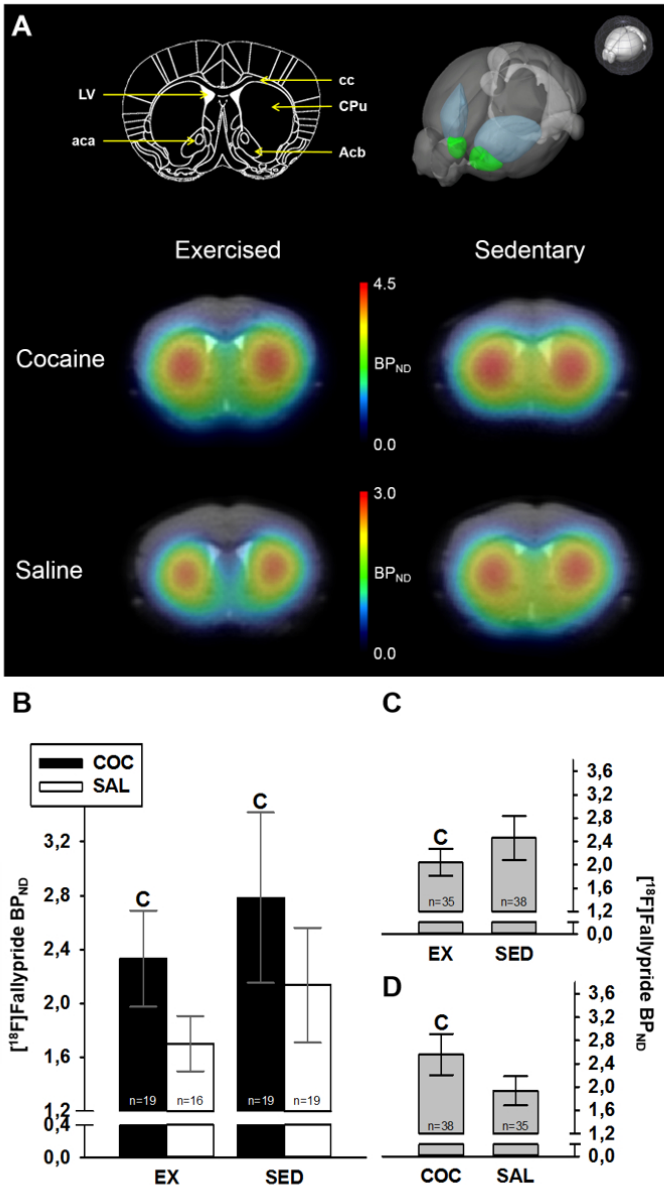

Figure 5

176x315mm (96 x 96 DPI)

Supplementary data for

Exercise Against Cocaine Sensitization In Mice: A [^{18}F]Fallypride Micro-PET Study.

Guillaume Becker[1,§,*], Louis-Ferdinand Lespine[2,3,§], Mohamed Ali Bahri[1], Maria Elisa Serrano[1], Christian Lemaire[1], André Luxen[1], Ezio Tirelli[2] and Alain Plenevaux[1]

[1] GIGA – Cyclotron Research Center – In Vivo Imaging, University of Liège.

[2] Department of Psychology, University of Liège.

[3] Pôle MOPHA, Pôle Est, Centre Hospitalier Le Vinatier, Bron, France

[§] These authors contributed equally to this work.

* Corresponding author:

Guillaume Becker

Email: g.becker@uliege.be

Allée du 6 Août, 8. Quartier Agora, B30, 4000 Liège, Belgium.

ORCID ID 0000-0002-1714-0267

Current address:

Laboratoire CarMeN, INSERM U1060, University Lyon1, INRA U1397, INSA Lyon, Hospices Civils Lyon.

Groupement Hospitalier Est, 59 Boulevard Pinel, 69 500 BRON, France

List of supplemental data:

Supplementary Figure S1. Cerebellum TACs (expressed as AUCs) homogeneity between groups.

Supplementary Figure S2. Randomized block design.

For Review Only

Supplementary Figure S1. Cerebellum TACs (expressed as AUCs) homogeneity between groups.

AUCs data set was treated according to a randomized block design with a fixed-model 2 x 2 ANOVA incorporating the housing condition (EX or SED; 2 levels) and pharmacological treatment (COC or SAL; 2 levels) as between-group factors, and with the lot as a blocking factor (with 24 levels)

No evidence for effect of aerobic exercise, cocaine, or interaction:

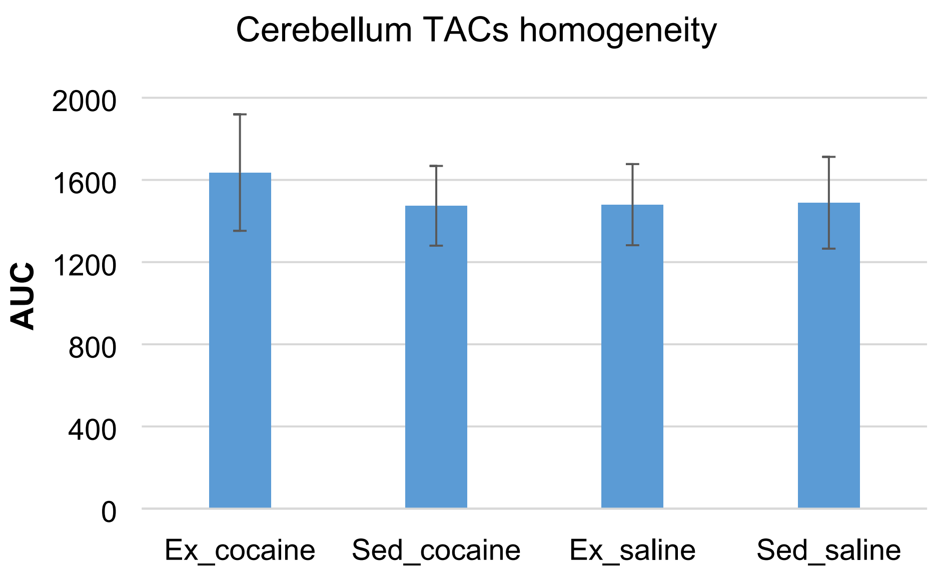

AUC ($\eta^2p = 0.05$, $F(1,46) = 2.610$, $p = 0.11$; $\eta^2p = 0.02$, $F(1,46) = 0.862$, $p = 0.36$; $\eta^2p = 0.02$, $F(1,46) = 0.797$, $p = 0.38$, respectively).

ew Only

Supplementary Figure S2. Randomized block design.

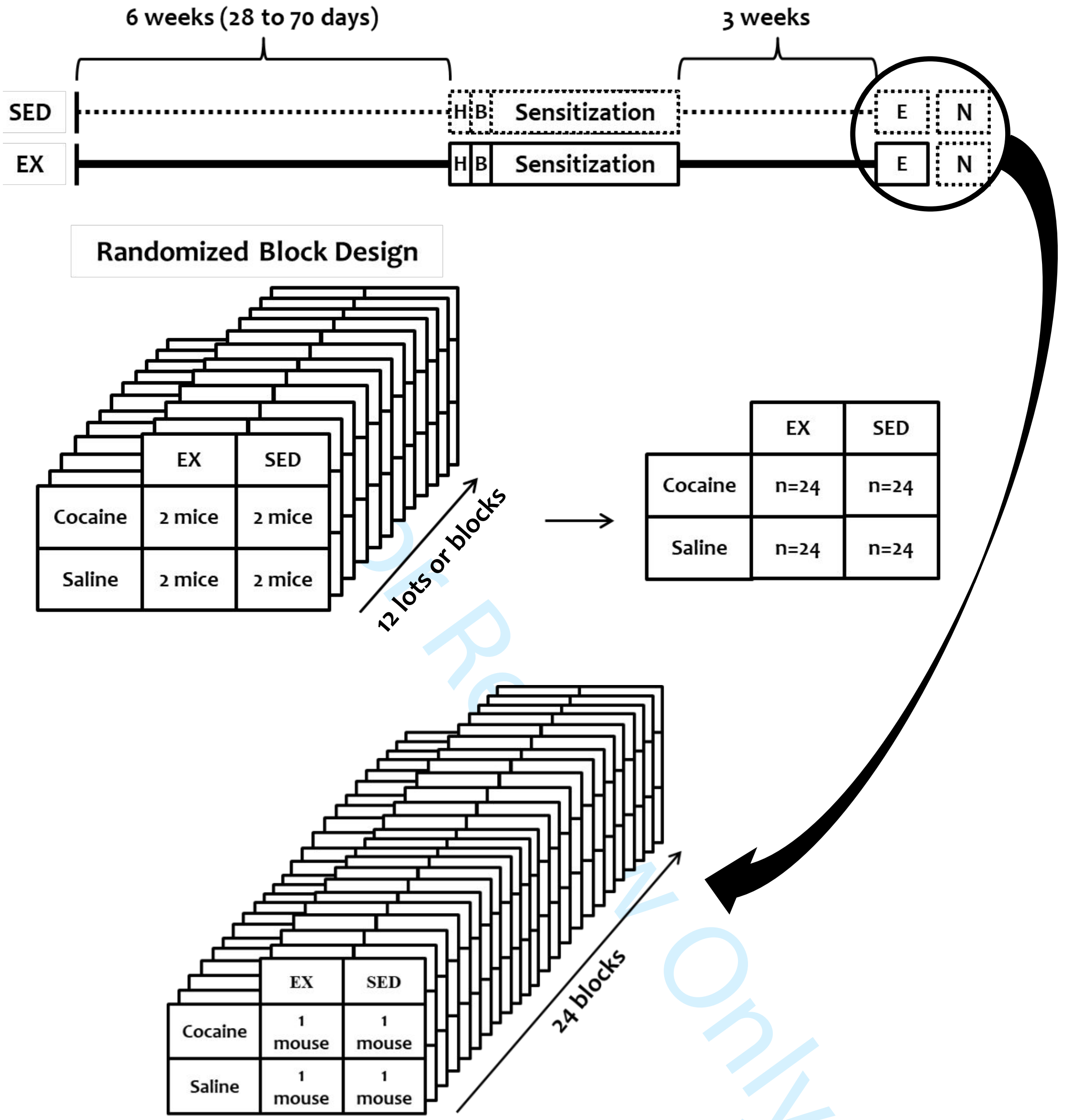

Due to practical reasons, the whole experiment was organized into twelve lots purchased and tested successively (each lot consisting of 8 mice). In each lot, two mice were assigned to one of the four experimental group by means of a computer-generated randomization schedule, the eight mice housed in the acclimation cages contributing to theses four possible groups (sedentary/cocaine, sedentary/saline, exercised/cocaine, and exercised/saline). Therefore, the four groups were systematically represented within each lot by 2 mice to take into account any between-lot variability as well as that due to the time and circumstances of testing (i.e. randomized block design). Additionally, due to impossibility to test 8 mice the same day in micro-PET, each block (n=8) was further split into 2 blocks (n=4) for the test for expression of sensitization and micro-PET procedures. Again, the four groups were systematically represented within each block by one mouse. Therefore, mice were tested for expression of sensitization either 30 (half) or 32 (other half) days after the last cocaine injection, while all mice underwent neuroimaging scan 24h after this test.

For Review Only
