# Supplementary material for: Exercise against cocaine sensitization in mice: a [18F]fallypride micro-PET study
Source: Brain Commun. 2021 Dec 15;4(1):fcab294. doi: 10.1093/braincomms/fcab294 (PMC8833578; doi:10.1093/braincomms/fcab294)
Supplement: fcab294_Supplementary_Data [file Original Submission-Responses_to_reviewers.pdf › Original Submission-Responses_to_reviewers.pdf]

Letter of response to the reviewers' comments.

Reviewer #1: Comments and responses

Comment #1:
The relevance of a sensitized locomotor response to non-contingent, experimenter-delivered administration of cocaine to the understanding of addiction remains elusive. It would be very helpful to have a better justification than a citation related to its potential relevance for relapse and craving: is relapse and/or craving (a subjective state) somewhat related to the locomotor activating effects of cocaine in humans?
Sensitisation is a behavioural manifestation of an anticipatory pavlovian response (conditioned locomotion added to the activating effects of the drug over time), but it is not presented as such in this study, thereby making it difficult to understand what is being discussed.

Response:
We thank Reviewer #1 for this helpful comment. We agree that the relevance of locomotor sensitization to the understanding of addiction in the introduction was "elusive" and we have made changes in the revised manuscript with relevant citations (p.3, l. 56):

Initial version:
"Wheel-running exercise has also been shown to be effective at reducing the acute and chronic locomotor-stimulating effects of cocaine as well as the sensitization to those effects, a phenomenon thought to play an integral role in craving and relapse."

Revised version:
"Wheel-running exercise has also been shown to be effective at reducing the acute and chronic locomotor-stimulating effects of cocaine as well as the sensitization to those effects, a phenomenon that has been implied in the shift from recreational use to pathological abuse, i.e. in the pathogenesis of addiction[12,13], and suggested to play a role in animal models of drug self-administration.[14–16]"

Nevertheless, we would like to emphasize that a substantial amount of preclinical literature over past decades has extensively studied the features of both acute and chronic psychomotor effects (e.g., locomotion) of cocaine (among others drugs of abuse). Although this set of research does not speak (directly) to the question of vulnerability to cocaine addiction, it addresses the question of initial vulnerability to cocaine responsiveness and its components and determinants. This should not be underestimated due to evidence in both humans and animals that initial response to addictive substances can predict subsequent use and abuse (for a review, see de Wit & Phillips 2012 Neurosci Biobehav Rev 36(6): 1565–1576)[1].

In both rats and mice, acute and chronic locomotor activation following drugs administration has been used as a measure of initial sensitivity to those substances. For example, many studies have shown that hyperlocomotion induced by amphetamine and cocaine could predict active drug-seeking behaviors (self-administration). More specifically, elevated lomocotor activation was associated with higher levels of self-administration (Deminiere et al. 1989, Neurosci Biobehav Rev, 13:141–147; Piazza et al. 1989, Science, 245:1511–1513; Mantsch et al. 2001, Psychopharmacology, 157: 31–39).[2–4] Other studies in mice have also provided evidence for a positive association between initial locomotor response to cocaine and motivation for self-administration as measured by breakpoints (e.g., Roberts et al. 2018, Psychopharmacology, 235(4): 1179-1189).[5]

It goes without saying that cocaine self-administration, mostly done via the intravenous route, is a more appropriate and valuable paradigm in assessing vulnerability to its motivational properties. However, Reviewer #1 will probably acknowledge that the technical feasibility is particularly limited in mice compared to larger animals (rats). Even if our psychopharmacological outcomes (acute and sensitized locomotor response to cocaine) are not the most commonly employed in our field, Reviewer 1 will certainly agree on the scientific usefulness to try investigating a physiological or behavioural effect with more than one experimental model/paradigm.

In regards with the second part of Reviewer's 1 comment (sensitisation as a behavioural manifestation of an anticipatory pavlovian response added to the activating effects of the drug over time, and not presented as such in this study), we do not believe that the anticipatory pavlovian component of locomotor sensitization needs to be explicitly mentioned.

First and foremost, the anticipatory pavlovian component of locomotor sensitization (as typically revealed in the experimental context by challenging with saline the animals that have paired the drug effect with that context) was not an outcome of interest in this study.

Second, although it is often believed that contextual sensitized effects are modulated by, if not rely on, pavlovian conditioning processes (Stewart & Badiani, Behav Pharmacol 1993; 4: 289–312; Crombag et al. Behav Brain Res 2000; 116: 1–22; Carey & Gui Behav Brain Res 1998; 92: 67–76; Tirelli & Terry, Behav Pharmacol 1998; 9: 409–19)[6–9], it must be kept in mind that the role of pavlovian excitatory conditioning accounting for contextual sensitization has been challenged in many papers, in which a clear-cut dissociation between the post-sensitization conditioned response (in drug-free conditions) and the contextually sensitized response (following drug administration) has been unambiguously found. For example, contextual sensitization to amphetamine- and cocaine-induced hyperlocomotion in rats and mice persists without any decrease in amplitude after extinction of the conditioned response, instead of being extinguished as well (Drew & Glick, Psychopharmacology 1988; 95: 482–7; Stewart & Vezina Behav Pharmacol 1991; 2:65–71; for critical discussion see Carey & Gui, Behav Brain Res 1998; 92: 67–76)[7,10,11]. Also, strong contextually sensitized locomotor responses to apomorphine or quinpirole in rats have been obtained, without any conditioned response after a saline challenge (Mattingly & Gotsick, Behav Neurosci 1989; 103:1311–7; Szechtman et al. Behav Pharmacol 1993; 4: 405–10).[12,13] Conversely, conditioned hyperactivity in amphetamine-pretreated rats has been reported in the absence of prior contextual sensitization of the response (Mazurski & Beninger, Pharmacol Biochem Behav 1987; 27: 61–5).[14] Other studies have shown that the contextual sensitization and the conditioned response are differentially sensitive to pharmacological blockade, i.e. dissociable (Reimer & Martin-Iverson Psychopharmacology 1994; 113: 404–10; Mattingly et al. Psychopharmacology, 1998; 140: 387–97).[15,16]

Additionally, no correlation has been found between the post-sensitization conditioned response and several parameters of contextual sensitization, challenging one of the most important predictions of the assumption that a pavlovian excitatory conditioning may account for contextual sensitization (Michel & Tirelli Behav Brain Res 2002; 132: 179–86; Michel & Tirelli Behav Pharmacol 2002; 13: 59–71; Tirelli et al. 2003 Eur Neuropsychopharmacol 13(4): 289-96).[17–19]

Comment #2:
Is it the case that a differential sensitivity to the reinforcing properties of addictive drugs is a valid marker of vulnerability to addiction?

Response:
Reviewer #1 will probably acknowledge that the answer to that comment can be found in our response to the previous comment (comment #1), that we reproduce below:

There is evidence in both humans and animals that initial response to addictive substances can predict subsequent use and abuse (for a review, see de Wit & Phillips 2012 Neurosci Biobehav Rev 36(6): 1565–1576).[1] In both rats and mice, acute and chronic locomotor activation following drugs administration has been used as a measure of initial sensitivity to those substances. For example, many studies have shown that hyperlocomotion induced by amphetamine and cocaine could predict active drug-seeking behaviors (self-administration). More specifically, elevated lomocotor activation was associated with higher levels of self-administration (Deminiere et al. 1989, Neurosci Biobehav Rev, 13:141–147; Piazza et al. 1989, Science, 245:1511–1513; Mantsch et al. 2001, Psychopharmacology, 157: 31–39).[2–4] Other studies in mice have also provided evidence for a positive association between initial locomotor response to cocaine and motivation for self-administration as measured by breakpoints (e.g., Roberts et al. 2018, Psychopharmacology, 235(4): 1179-1189).[5]

Comment #3:
Is it not the case that high impulsivity trait, a marker of addition vulnerability in humans and rodents, is associated with decreased D2/3 striatal binding, as measured with PET?

Response:
We thank the reviewer for this highly relevant comment. The reviewer is probably referring to the works of Buckholtz et al. for human (Buckholtz et al. Science, 2010; 329 (5991):532-532)[20], and Dalley et al./Caprioli et al. for rodents (Dalley et al. Science, 2007;315(5816):1267-1270; Caprioli Neuropsychopharmacology. 2013;38(8):1460-1471).[21,22] These studies interestingly revealed indeed an inverse correlation between D2/3 R availability (assessed through [^{18}F]Fallypride PET) and high impulsivity phenotype. Although there are slight differences between these phenomena. On the one hand, the work of Buckholtz et al. strikingly showed **in humans** that higher levels of trait impulsivity were predicted by **diminished midbrain D2/D3 R availability**. As there were lying in the midbrain, those receptors were identified as D2/D3 autoreceptor. Consistently with inhibitory influence, this decrease in midbrain D2/D3 autoreceptor binding was associated to an **increase in amphetamine-induced DA release** in the striatum. On the other hand, studies revealed that high impulsivity in **rats** is a trait related to a significantly **reduced D2/3 R availability in the nucleus accumbens**.[21] Besides, they specified that this decrease in D2/3 R is **not related to variations in striatal DA release**. Interestingly, the same team revealed that the reduction in striatal D2/3 R availability at baseline is further normalized by cocaine exposure which also reduced impulsivity in highly impulsive rats.[22]
As such, we acknowledge the apparent discrepancies between the above discussed studies and our results. Although the striatal D2/3 R availability reduction we described in our experimental setting is an example of exercise-induced brain plasticity and is therefore related to a general impact of physical exercise on the brain. On the contrary, studies on high impulsive rats highlighted the specificity of DA involvement in impulsive behavior. Therefore, mechanisms leading to reduced D2/3 R availability could be totally different between the two conditions.

Besides, methodological considerations need to be highlighted. Studies on impulsivity assigned rats to high and low impulsive groups on the basis of behavioral outcomes of the five-choice serial reaction time task. Consequently, the significant difference in striatal D2/3 R availability (lower availability for the high impulsive rats) is a baseline state for the animal in these studies. Whereas in our study, we randomly assigned the mice in the four groups before any treatment's application (neither exercise nor drug injection). Albeit impulsive behaviors have been described in C57Bl6 mice (Isles et al. J Neurosci. 2004;24(30):6733-6740; Gubner et al. Alcohol Clin Exp Res. 2010;34(8):1353-1362).[23,24], the randomization should have normalized the number of impulsive individuals between the four groups, as well as the basal striatal D2/3 R availability.
As we mentioned in the limitation part of our manuscript, the use of microPET imaging in mice preclude the separate analyze of ventral and dorsal striatum. In a review on mechanisms involved in rodent models of impulsivity, Jupp and coll. highlighted that nucleus accumbens core and shell may react differently to DA stimulants (cocaine/amphetamine) in impulsive animals (Jupp et al. DMM Dis Model Mech. 2013;6(2):302-311).[25] In parallel, we also mention that the radiotracer [^{18}F]Fallypride cannot differentiate D2 R from D3 R whereas D3 R have specific role in addiction. Those issues, in our context of exercise-induced reduction in striatal D2/3 R availability, would definitely need further clarifications that microPET alone in mice is not able to address.
Finally, the reviewer 's comment raises a very interesting question on the interplay between physical exercise and impulsive behavior. Although this point is out of the scope of our study, it would be very interesting to address the potential effect of exercise on impulsive behavior. The potential therapeutic involvement of physical exercise in Attention Deficit and Hyperactivity Disorder (ADHD) (Tsai et al. Front Hum Neurosci. 2021;15(June):1-13)[26] highlight the interest of such studies, as high impulsive rodents are thought to be relevant model for the study of ADHD.

Comment #4:
In humans, the subjective report of euphoria or high associated with the administration of methylphenidate is negatively correlated with the level of D2/3 DA receptors in the striatum (see elegant work from Volkow). How do the authors reconcile the effect of exercise on D2/3 receptors levels and their suggestion that it represents a biological signature of the protective effect of exercise on addiction vulnerability?

Response:
The reviewer referred appropriately to a study of Volkow and coll. investigating the reinforcing effects of psychostimulants in humans using [^{11}C]Raclopride PET imaging (Volkow et al. J Pharmacol Exp Ther. 1999 Oct;291(1):409-15).[27]. Thanks to a drug occupancy design, this study nicely showed a positive correlation between the intensity of the self-reporting drug effect (i.e. "high") induced by methylphenidate and the levels of released dopamine. They were able to demonstrate that reinforcing effects of drugs in humans (reflected by subjective reporting of stimulant-induced high), is associated with increases in brain dopamine release. Nevertheless, the use of a drug occupancy PET design with intravenous injection of methylphenidate relates on acute measure of synaptic DA in striatal synapses whereas our study, comparing exercise mice to their sedentary counterparts, relates to a reduced DA tone in a chronic state (at basal level, without any pharmacological challenge or stimulation). Although we clearly acknowledge in the main text (p.17, l.409) that "our current data do not allow us to unravel the mechanism of decreased D2/3R availability which can be related to a decreased D2/3R density or an increase in endogenous synaptic DA".

Comment #5.
Could it be the case that mice used to run on a wheel then exclusively run on a wheel (perhaps accounting for the seemingly lower level of baseline locomotor activity in EX mice as compared to SED mice – effect not reported in the manuscript), hence it would be very informative to provide a wheel in the test context at the time the expression of the sensitized response to cocaine is measured.

Response:
We might not have fully understand this comment.
However, any locomotor measures (either running wheel distances or locomotor activity off the wheel) would not be comparable between exercised and sedentary animals, due to the novelty of the context for the latter, and thus expected differential use of the wheel. The assessment of exercise on cocaine responsiveness would be highly biased.

Comment #6.
According to T. Robinson, the behavioural manifestation of sensitisation follows an inverted u-shaped curve in that animals will stop running to engage in an ever narrowing repertoire of stereotypic behaviours when sensitised. Have these behaviours been measured here?

Response:
The psychomotor activation induced by cocaine represents a complex set of different behaviors, which can compete with one another. The most frequent psychomotor effect studied involves the measure of locomotor activity, as we did here. However, cocaine can produce many other psychomotor effects, including complex patterns of repetitive motor actions (e.g. stereotyped behaviors). Which psychomotor effect dominates and how it develops depend on many factors including the dose and age. Importantly, and as mentioned by the reviewer #1, these different psychomotor effects can be dissociated, and they do not all change uniformly as a function of repeated drug administration.

Unfortunately, we were not able to measure these behaviors. That being said, while cocaine can produce compulsive and stereotype behaviors (such as licking and gnawing), with no further increase or even a reduction in locomotion and exploration (as revealed sometimes by an inverted u-shaped curve), this pattern did not occur in our study (see Fig 3 panel A). This is not surprising as such pattern generally occurs at higher doses than the one used here. Instead, the dose we used is known to stimulate explorative and locomotor activity (e.g., Tirelli et al. 2003 Neuroscience and Biobehavioral Reviews 27,2003 163–178).[19]
Note that it would be very interesting to assess how exercise might impact the sensitization to stereotyped behaviors (i.e., repeated administrations of high cocaine doses) — an animal model of psychotic schizophrenia-like symptoms (Sams-Dodd F. 1999 Rev Neurosci 1999;10:59–90).[28] However, this was beyond the scope of our study.

Comment #7.
Why were the wheels only removed in half the EX population prior to the PET session? Rather than controlling for a potential effect of wheel running on the outcome of the PET measurements, the removal of the wheel can have triggered a massive stress in animals that have had access to it daily. This is a major confounding factor.

Response:

- **Why the wheels were only removed in half the EX population prior to the PET session? :**

The wheels were effectively removed ***in all EX mice*** (as stated p. 10, l. 239): "Note that the wheels (for exercised mice) were removed 24h before neuroimaging measurement to avoid any potential effect of overnight wheel-running exercise on neuro-functional measures."

- **The removal of the wheel can have triggered a massive stress in animals that have had access to it daily. This is a major confounding factor:**

We appreciate this comment. We acknowledge that this is (highly) counter-intuitive, but the removal of the wheel does not induce a massive stress in animals used to have continuous access to it. Previous papers from our laboratory (Lespine & Tirelli, 2015; Lespine & Tirelli, 2018) explicitly tested this hypothesis and disconfirmed it.[29,30]

A few studies have found accentuated levels of drug responsiveness or "anxiety" in rats and mice undergoing cessation of free wheel-running prior to testing (Engelmann et al 2014 Brain Structure and Function 219, 657–672; Nishijima et al 2013 Behavioural Brain Research 245, 34-41).[31,32] In Engelmann et al 2014 study, rats housed with a running wheel for several weeks and tested after exercise cessation exhibited accentuated levels of methamphetamine self-administration, as compared with the sedentary rats. In another, non-pharmacological, study (Nishijima et al 2013 study), higher levels of "anxiety" were found in C57BL6J mice that had lived for 8 weeks with a running wheel before being deprived of it.

Such an accentuation is a sort of deprivation-like effect reflecting the stress induced by exercise cessation, which could thus be considered as an acute stressor by itself. Also, the inhibiting effects that wheel-running exert on drug effects (without exercise cessation) have been ascribed to a reduction of moderate stress and anxiety since these negative emotions were found to be tempered by exercise in non-pharmacological studies. In the absence of a running wheel, these negative emotions are thought to be instilled by the poor living environment of singly-housed sedentary animals and concomitantly by the drugs (cocaine in our case), which are known to mobilize on their own the brain stress-responsive systems. In other words, exercise-inhibited drug effects may be mediated by exercise-reduced stress. That explanatory framework is sometimes referred as to the anti-stress (or stress) view of the "anti-drug" effects of exercise.

The anti-stress view predicts that cessation of exercise will be followed by an accentuation of a drug effect (e.g., cocaine sensitization). We disconfirmed the anti-stress prediction (no significant accentuation of sensitization) which is also to say that wheel-running exerted a persistent protective effect against psychomotor sensitization to cocaine (Lespine & Tirelli, 2015; Lespine & Tirelli, 2018).[29,30] One can hypothesize that the long-term anti-cocaine effects of exercise was due to its lasting anti-stress effects. This explanation is partly compatible with the delayed increase in anxiety levels produced by exercise cessation in rats (Greenwood et al. 2012 Behavioural Brain Research 233, 314-321).[33]

That being said, the disparities between our results and those of Engelmann et al 2014 and Nishijima et al 2013 are still difficult to reconcile. This may arise from variations in experimental model, species, and drug. On the whole, given the scarcity of studies purposely designed to test such a hypothesis, one should reasonably admit that the mediating role of the anxiolytic properties of exercise in its reducing effects of drug vulnerability has yet to be satisfyingly characterized and understood.

Therefore, in absence of any observations and knowledge regarding the potential effect of overnight wheel-running on neuro-functional measures, we decided to remove the wheels. Again, this choice was based on consistent observations (at least in our lab) that such procedure (wheel removal) has no stress-related effects.

Comment #8.
The absence of a sal vs coc design at test (expression of sensitization) makes it difficult to appreciate the magnitude of sensitization here: what is the effect of an acute cocaine injection in mice with a history of repeated saline administration and associated exposure to the novel test box and, more importantly, what is the magnitude of the conditioned response to the injection in cocaine treated mice (which can only be revealed in a coc-treatment – sal-challenge group)?

Response:
We thank the reviewer #1 for their comment. We did not incorporate "coc-treatment—sal-challenge" and "sal-treatment—coc-challenge" groups because the primary purpose of our study did not require it. In fact, these groups are not necessary to give an interpretation of the reported findings i.e., protective effects of exercise against acute and chronic hyperlocomotion induced by cocaine.

Perhaps, a 2x2x2 design as described in the table below could have been useful to complete the interpretation of the effects of exercise on the pavlovian conditioning processes of sensitization (although see our response to the Comment #1).

	Groups used in the study	*Not used*
Exercised	Coc treatment — Coc challenge	*Coc treatment —* ***Sal challenge***
Exercised	Sal treatment — Sal challenge	*Sal treatment —* ***Coc challenge***
Sedentary	Coc treatment — Coc challenge	*Coc treatment —* ***Sal challenge***
Sedentary	Sal treatment — Sal challenge	*Sal treatment —* ***Coc challenge***

In fact, whatever effect exercise would have on post-sensitization conditioned response, it would have not changed the validity of the conclusion that exercise induces attenuating effects of hyperlocomotor effects of cocaine. Moreover, this would have made our design much more complex and thus ethically problematic (i.e., doubling the number of animals with no (or little) incremental scientific value).

Comment #9.
The justification for an approach by contrasts, instead of a post-hoc interrogation of the main effects is not clear. Why would the pairwise comparisons not be carried with a Newman Keuls or a Sidak/Tuckey?

Response.
Similar to our previous studies (e.g., Lespine & Tirelli 2018; Lespine et al. 2019)[30,34], we chose an approach by contrasts (instead of post hoc tests) because we had a planned set of comparisons, the set of contrasts being determined by the logic of our experiment and hypotheses based on our previous studies. For instance, we planned to test cocaine groups vs their corresponding saline groups; expecting higher levels of locomotor activity in cocaine-

receiving mice. However, we were not interested to compare (for example) sed-coc vs. ex-sal groups.

Post-hoc tests are performed after obtaining a significant effect for a factor, where all possible comparisons might be considered (i.e., no prior expectation about what differences might be seen). In other words, comparisons are not planned in advance. One of the important advantages of planned comparisons is the protection they offer against Type II error (Rosenthal, R.L. Rosnow, Contrast Analysis: Focused Comparisons in the Analysis of Variance, Cambridge University Press, NY, 2009).[35]

Also, we have made changes in the revised manuscript (p. 11, l. 273):

Initial version:
"Each set of data was treated according to a randomized block design with a fixed-model 2 x 2 ANOVA incorporating the housing condition (EX or SED; 2 levels) and pharmacological treatment (COC or SAL; 2 levels) as between-group factors, and with the lot as a blocking factor (with 12 or 24 levels for the behavioral and neuro-functional measures respectively, see Supp. S1). This was followed by planned crossed or simple contrasts (42). Each contrast was derived from the mean-square error term (MSE) provided by the ANOVA. Based on previous experiments or preliminary results, exercised mice were expected to display (1) lower cocaine locomotor responsiveness than sedentary mice (crossed contrasts) and (2) lower values of BPND (simple contrast). Cocaine-receiving mice were expected to show (1) greater locomotor activity and (2) higher BPND values than control saline mice (simple contrasts)."

Revised version:
"Each set of data was treated according to a randomized block design with a fixed-model 2 x 2 ANOVA incorporating the housing condition (EX or SED; 2 levels) and pharmacological treatment (COC or SAL; 2 levels) as between-group factors, and with the lot as a blocking factor (with 12 or 24 levels for the behavioral and neuro-functional measures respectively, see Supp. S1). The primary outcomes, defined by our a priori hypotheses described below, were given by planned crossed or simple contrasts providing better protection against Type II error (42). Each contrast was derived from the mean-square error term (MSE) provided by the ANOVA. Based on previous experiments or preliminary results, exercised mice were expected to display (1) lower cocaine locomotor responsiveness than sedentary mice (crossed contrasts testing interaction) and (2) lower values of BP_{ND} (simple contrast). Cocaine-receiving mice were expected to show (1) greater locomotor activity and (2) higher BPND values than control saline mice (simple contrasts)."

Comment #10.
PET does not distinguish between states of DARs, as well pointed out in the manuscript. It does not distinguish either between pre- and post-synaptic receptors. When searching through the literature on D2 DA receptors in VTA vs SNc neurons, it appears that an up-regulation of D2R mRNA occurs in mice with a history of cocaine sensitization in dopaminergic neurons per se (10.1007/s00213-007-0790-3).

Response.
We would like to thank the reviewer for this very important point. The instructive study mentioned above effectively reveals an increase in D2R mRNA in C57Bl6 mice with an history of cocaine. Although experimental considerations deserve specific attention. Mice that display

enhanced D2R mRNA are challenged with vehicle at the time of sensitization expression (cocaine history – vehicle challenge = coc – veh). The authors reminds that coc – veh mice have repeatedly experienced the effects of cocaine in a new environment (activity cage), we might hypothesize that this D2R mRNA increase could be partly related to the cue triggered by the activity cage environment.

Most of all, the key element discuss by the authors is the lack of any difference in D2R mRNA expression between coc – coc mice and veh – coc mice (i.e. mice with history of cocaine and challenged with cocaine at the time of sensitization, coc – coc ; versus mice without history of cocaine and challenged with cocaine at the time of sensitization, veh – coc). They reported: "No difference was observed between coc–coc and veh–coc mice regarding the D2 mRNA level; **we thus conclude that D2 autoreceptor mRNA level is not altered after the expression of behavioural sensitization**." (Belin et al. Psychopharmacology. 2007;193(4):567-578)[36]

Reviewer #2: Comments and responses

Comment #1.

Firstly, the authors need a clearer rationale for studying sensitization as an explanatory mechanism for addiction. The introduction is written in such a way to suggest that the experimental phenomenon of sensitization is central to the persistence of drug use. The authors may wish to be more circumspect about the necessary and sufficient involvement of sensitization in addiction and moreover take a less dopamine-centric view of underlying neurochemical mechanisms.

Response.

We thank the reviewer #2 for this helpful comment. We have made changes in the revised manuscript with relevant citations. As suggested, we are now "more circumspect about the necessary and sufficient involvement of sensitization in addiction" (p.3, l. 56).

Initial version:

"Wheel-running exercise has also been shown to be effective at reducing the acute and chronic locomotor-stimulating effects of cocaine as well as the sensitization to those effects, a phenomenon thought to play an integral role in craving and relapse."

Revised version:

" Wheel-running exercise has also been shown to be effective at reducing the acute and chronic locomotor-stimulating effects of cocaine as well as the sensitization to those effects[10,11], a phenomenon that has been implied in the shift from recreational use to pathological abuse, i.e. in the pathogenesis of addiction[12,13], and suggested to play a role in animal models of drug self-administration.[14–16] "

We reproduce below our response to Reviewer #1 who commented on this introductory section as well:

We would like to emphasize that a substantial amount of preclinical literature over past decades has extensively studied the features of both acute and chronic psychomotor effects (e.g., locomotion) of cocaine (among others drugs of abuse). Although this set of research does not speak (directly) to the question of vulnerability to cocaine addiction, it addresses the question of initial vulnerability to cocaine responsiveness and its components and determinants. This should not be underestimated due to evidence in both humans and animals that initial response

to addictive substances can predict subsequent use and abuse (for a review, see de Wit & Phillips 2012 Neurosci Biobehav Rev 36(6): 1565–1576).[1]

In both rats and mice, acute and chronic locomotor activation following drugs administration has been used as a measure of initial sensitivity to those substances. For example, many studies have shown that hyperlocomotion induced by amphetamine and cocaine could predict active drug-seeking behaviors (self-administration). More specifically, elevated lomocotor activation was associated with higher levels of self-administration (Deminiere et al. 1989, Neurosci Biobehav Rev, 13:141–147; Piazza et al. 1989, Science, 245:1511–1513; Mantsch et al. 2001, Psychopharmacology, 157: 31–39).[2–4] Other studies in mice have also provided evidence for a positive association between initial locomotor response to cocaine and motivation for self-administration as measured by breakpoints (e.g., Roberts et al. 2018, Psychopharmacology, 235(4): 1179-1189).[5]

It goes without saying that cocaine self-administration, mostly done via the intravenous route, is a more appropriate and valuable paradigm in assessing vulnerability to its motivational properties. However, the Reviewers will probably acknowledge that such experimental procedures require high technical resources and skills (that we do not have in our lab unfortunately). The technical feasibility is particularly limited in mice compared to larger animals (rats). Even if our psychopharmacological outcomes (acute and sensitized locomotor response to cocaine) are not the most commonly employed in our field, the Reviewers will certainly agree on the scientific usefulness to try investigating a physiological or behavioural effect with more than one experimental model/paradigm.

As recommended by the reviewer, we have made changes in the revised manuscript with relevant citations to adopt "a less dopamine-centric view of underlying neurochemical mechanisms" (p. 3, l. 61):

Initial version:
"In vivo neuro-imaging has greatly impacted our understanding of pathophysiology of the chronic brain state of addiction.[14–16] Imaging studies have evidenced a decreased dopamine D2 receptors availability in the dorsal striatum of patients suffering from cocaine addiction.[17]"

Revised version:
"In vivo neuro-imaging has greatly impacted our understanding of pathophysiology of the chronic brain state of addiction.[17–19] Drugs of abuse are known to induce brain plasticity, notably through changes in neurotransmitter release as well as in pre-and post-synaptic signal transduction. These neuroadaptations underlying the pharmacology of addiction disorders were described particularly for glutamate, through AMPA and metabotropic receptors, and GABA neurotransmissions.[20–27] Monoaminergic systems are the main targets of psychostimulant drugs like amphetamine and cocaine. Notably, dopamine signalling has received considerable attention. Imaging studies have evidenced a decreased dopamine D2 receptors availability in the dorsal striatum of patients suffering from cocaine addiction.[28]"

Comment #2.

- **Secondly, and more problematically, the findings run counter to the rich corpus of studies showing reduced D2Rs as a striatal biomarker of addiction. This discrepancy should be addressed, possibly at the level of the experimental protocol used in the present study. It would be helpful if the authors referred to the work**

of Isabelle Boileau (2006), one of the first PET demonstrations of sensitization in humans. Of note, in this study, no changes in striatal D2R binding potential were reported following several doses of d-amphetamine (discussed on page 1392 – Boileau et al., 2006).

Response.
The reviewer's comment rises a crucial point and allows us to precise the focus of our study, which is not addiction per se, but the specific phenomenon of sensitization to psychostimulant. Therefore, rather than showing discrepancy with the "rich corpus of studies showing reduced D2Rs as a striatal biomarker of addiction", our results should be interpretated in the specific context of drug sensitization, a phenomenon that could account for the development of drug seeking behaviours. Of note, the work of Isabelle Boileau (2006), mentioned above, reports "no change in striatal D2R binding potential were reported following several doses of d-amphetamine" as highlighted by the reviewer. Those results would also present this apparent discrepancy with a "reduced D2Rs as a striatal biomarker of addiction". On the contrary, there were interpreted within the precise framework of sensitization phenomenon.
We clarified this point in the manuscript (p. 4, l. 78): "Within this specific context of sensitization to cocaine in mice, …"

Moreover, the reviewer rightly remind that literature largely agree on a reduced striatal D2Rs in relation to addiction disorder. Although this is now accepted in humans and non-human primates imaging studies, imaging results in rodent are more conflicting with reports of increases, unchanged and decreases D2R availability. This is stated in the introduction (p. 3, l. 70-72) and was well reported in a reviewed published by Narendran and Martinez (Narendran & Martinez, Synapse. 2008;62(11):851-869). [37]
We would like to thank the reviewer for mentioning the study of Boileau et al. which perfectly highlight the sensitization process and its relation to some aspects of drug-induced neuroadaptive changes. Although some experimental differences warrant mention. The study of Boileau et al. investigated the impact of amphetamine sensitization on the baseline of [^{11}C]raclopride binding potential in a group of five subjects with history of amphetamine injection and no challenge at the time of testing. As discuss in the paragraph below, we didn't include such a group in our present study since that would have meant to divide each group by two, at the time of testing, to test the condition: history of cocaine and vehicle at testing. However, we prioritize the statistical power for this replication study with the aim to establish sounds results for the design of further studies. Furthermore, and beyond the interspecies variations, in our experimental design PET imaging sessions took place 24 hours after the cocaine/saline challenge of sensitization (to investigate basal level of D2/3R availability), whereas the study of Boileau et al. scanned the individuals at the time of amphetamine/saline injection. This difference in experimental design may account for the discrepancy in the D2/3 PET outcomes.
As recommended by the reviewer, we discuss this study in the discussion of our manuscript (p. 19, l. 458):

"The sensitization phenomenon has been achieved in healthy men in laboratory context by Boileau and coll.[79] The authors reported an increased dopamine release after repeated exposure to amphetamine. Of note, in this study, no changes in striatal D2R binding potential were reported following several doses of d-amphetamine. Beyond the interspecies variations, experimental design may account for this discrepancy (e.g. single sex design with men only in the study of Boileau et al.; not the same control groups)."

- **Unfortunately, the interpretation of the present findings is constrained by scanning at a single time point only. The true power of PET lies in longitudinal assessment - in this case, pre and post cocaine. It is a moot point whether the observed increase in D2R binding potential would persist for days or even weeks; it may well decompensate after an extended period. The authors should acknowledge this point and discuss any relevant evidence for the temporal dynamics of D2R binding potential in experimental approaches of sensitization in rodents, including D3Rs.**

Response.
The reviewer remark is highly justified. We agree that adding a late PET scan time point would have been ideal. Although our aim was to build a robust experimental platform, reproducing our previous results and adding neurofunctional correlates. This constitutes a reliable starting point for further investigations following the same experimental protocol with replications and addition of new insights. Further on, we will add molecular investigations, new PET scanning time points (e.g. pre and post exposure at the time of sensitization expression, at the end of cocaine sensitization and at a late time point).

Behavioural sensitization in rodents is known to be a long lasting phenomenon and has been described up to 30 days after cessation of drug exposure.[38,39] The specific timeline of D2/3R involvement in cocaine sensitization still need to be investigated, although high affinity D2 receptors were modified up to 30 days after discontinuation.[40] Besides, molecular changes induced by drugs exposure were described even after 90 of abstinence.[41]
That being said, we cannot rule out any decompensation of the phenomena we described in the present study as the reviewer #2 mention. Indeed, decompensation of cocaine seeking behaviour has been described.[41]
One might hypothesize that the increased D2/3R availability related to long term expression of cocaine sensitization we described might progressively decrease on very long term with a rapid reinstatement at the time of re-exposure. Although this specific question was out of the scope of the present study.
Similarly, the protective effect of free wheel running on cocaine locomotor sensitization in C57Bl6 mice has been shown to resist at least 45 days after the cessation of physical exercise.[29,30] Therefore, one might suggest that the related decrease in D2/3R availability would persist for a similar period of time, although this remains to be validated trough specific experiments.

We followed the Reviewer's recommendation and added the following sentences (p.18, l. 420):
"Further studies are warranted to investigate the persistence of the impact of physical exercise on D2/3R availability similarly to what has been done in behavioural assessment, as well as the drug-induced plasticity we described in the present study."

Comment #3.
Thirdly, can the authors comment on whether the increase in D2R binding potential was a presynaptic or post-synaptic phenomenon. It is puzzling why the dorsal striatum was selected given overwhelming evidence implicating the mesolimbic dopamine system in sensitization (as the authors indeed acknowledge). A midbrain ROI would provide insight into presynaptic D2 receptors. A ventral striatal ROI would inform the spatial and possible temporal development of sensitization.

Response.
The reviewer rises here an important point, which is related to the remark #10 of the first referee. Thus, we would like to highlighted two highly relevant reviews (the one by Vanderschuren and Kalivas is cited in our manuscript) on cocaine sensitization written by Pierce and Kalivas, and Vanderschuren and Kalivas (Vanderschuren & Kalivas, Psychopharmacology. 2000;151(2-3):99-120; Pierce & Kalivas Brain Res Rev. 1997;25(2):192-216)[42,43], also cited in the work of Belin et al. mentioned above (point #10 reviewer #1). Those works stated that "**While the induction of sensitization seems to occur in the VTA, its expression is supposed to arise from the Nacc**".[38] As such we acknowledge that analyses in the ventral striatum would have brought meaningful insights. Although, as we highlighted in the limitation paragraphs of the discussion (p.18, l. 419), the limited spatial resolution of the microPET (1.5 mm) precludes a separated analyze of ventral and dorsal striatum in mice. This is one of the reasons why the mouse brain atlas of Mirrione et al./Ma et al. (Mirrione et al; Neuroimage. 2007;38(1):34-42; Ma et al. Neuroscience, 2005;135(4):1203-15).[44,45] implemented in the software that we used to analyze the data doesn't discriminate the 2 sub-regions. Besides, manually drawn ROIs in the ventral striatum would have been subjected to biases related to the precise localization of the ROI for a small region like ventral striatum in mice (and sharing a common boundary with the caudate-putamen), as well as to partial volume effect. Similarly, a midbrain ROI would have been indeed helpful, but VTA and SN in mice are so small that the signal to noise ratio was too weak and hindered a reliable quantification.
Regarding the pre- or post-synaptic localization of the DA receptors, [^{18}F]Fallypride cannot discriminate between the two localization. However, evidences suggest an involvement of post-synaptic receptors. Notably, Vandershuren and Kalivas well reviewed that somatodendritic and axonal D2 dopamine autoreceptors are only involved in short-term withdrawal through subsensitivity mechanism. On the contrary, at the time of **long term expression of the sensibilization (after a prolonged withdrawal period), as it is the case in our experimental setting,** subsensitivity of pre-synaptic D2 autoreceptors is not involved in enhanced sensitivity of dopamine nerve terminals.[42] Therefore, hypersensive locomotor stimulant effect triggered by D2 agonistes (De Vries et al. Neuropsychopharmacology. 2002;26(1):18-26)[46] could most probably occur through D2 post-synaptic receptors. Therefore, we suggest that our results may be related to post-synaptic receptors.

Comment #4.
Fourthly, I don't understand the logic of only assessing female mice. The replication of apparently sexually-dimorphic effects is important. The reliance on sensitization coupled with the singular analysis of females severely restricts the generality of the present findings.

Response.
We agree that the single-sex design constitutes a limitation of our study. An ideal design would have included male as well. To be completely honest, we did not have the (financial) resources to carry out a microPET study on 192 subjects (n=96/sex). Of course, we could have conducted our study using both sexes with n=12 per group instead of n=24 (it is not uncommon to see experiments in the literature using lower sample size than n=12). However, this would have considerably decreased the power of our study. Therefore, we decided to use female mice only with a relatively elevated sample size (at least for a preclinical study using inbred mice — where behavioral variability is lower compared to outbred mice — in a 2x2 factorial design). As stated in our manuscript, that we slightly revised (see below), females were preferred over male as they tend receive more benefits from exercise. More specifically, we previously showed that the long-term protection of a 3-week regimen of wheel-running exercise during adolescence

against acute and chronic psychomotor reactivity to cocaine was more pronounced in females (Lespine & Tirelli 2018).[30] In Lespine et al. 2019, we reported that the reducing effect of prenatal exercise on acute cocaine reactivity was strong in females (Cohen's d = 0.75), but not significant and modest in males (Cohen's d = 0.34).[34] Importantly, this set of results are in agreement with a recent review of sex differences in the interaction between exercise and drugs vulnerability indicating that females receive more benefits from exercise than males, in both humans and animals (Zhou et al 2016 Frontiers in Neuroendocrinology, 40, 24–41. https://doi.org/10.1016/j.yfrne.2015.07.001).[47]

Nevertheless, we agree that this limitation should be mentioned in the discussion (p. 18, l. 425): "Some limitations warrant mention. One of those relies on the single-sex design. We cannot rule out the possibility that male C57BL/6J mice could display a different pattern of results."

Comment #5.
Fifthly, can the authors really be sure their findings implicate exercise per se or more likely environmental enrichment?

Response.
One possible control (found sometimes in the literature) for potential effect of environmental enrichment is the use of locked wheels for "sedentary" mice.

Here, and as in our previous papers, "sedentary" mice were deliberately not housed in cages with locked wheels because mice climb in locked wheels, and we intended to keep physical activity to a minimum (Koteja et al 1999, Anim Behav 58:1307–1318; Rhodes et al 2003, Behav. Neurosci 117:1243–1256).[48,49] Climbing necessitates high energy expenditure and constitutes a form of physical exercise exerting at least some of the behavioral effects reported for wheel-running (Harri et al 1999, Lab Anim Sci 49: 401-405; Pietropaolo et al 2007, Behav Neurosci 121: 345-355).[50,51]

Importantly, numerous studies investigating wheel-running exercise on addictive properties of various drugs of abuse (e.g. alcohol, cocaine, and methamphetamine), through different experimental models (e.g., psychomotor sensitization, conditioned place preference and self-administration) did not use locked wheels as controls (see for example Darlington et al 2016 Genes Brain Behav, 15(5): 474–490; Engelmann et al 2014 Brain Struct Funct 219: 657-672; Mustroph et al 2011, Eur J Neurosci, 34: 1161-1169; Smith & Pitts 2012, Pharmacol Rep 64(4):960-4).[51,31,52,53] This was also the case in our previous published studies (Lespine & Tirelli 2015, Neuroscience, 310:650-64; Lespine & Tirelli 2018, Behav Brain Res, 349:63-72).[29,30] Therefore, we did not use locked wheels as control in order to facilitate comparisons with numerous previous studies.

Additionally, some studies suggest that the behavioural effects of locked running wheels are qualitatively and quantitatively different from those of unlocked wheels (Bednarczyk et al 2011, Hippocampus 21: 1334-1347; Dubreucq et al 2011, Hippocampus 21: 239-242; Dubreucq et al 2015, Behav Brain Res 282: 1-5; Pietropaolo et al 2006, Behav Neurosci 120: 787-803; Pietropaolo et al 2008, Behav Brain Res 192: 42-60).[54–58] For example, when the absence of wheel served as control, wheel-running decreased anxiety, facilitated conditioned freezing and had no influence on behavioral despair; with a locked wheel as control, wheel-running exerted no detectable effect on these two endpoints and increased behavioral despair (Dubreucq et al 2011, Hippocampus 21: 239-242).[57]

In other words, the effects of a running wheel may not exclusively include the effects of the locked wheel plus those induced by running on it, contrarily to ideal control and experimental groups.
We think that this constitutes an important experimental and inferential problem for the field of “exercise effects”, which warrants specific research and analysis. This, however, was beyond the scope of this study.

Comment #6.
Finally, it is noted that 23 mice were excluded due to technical difficulties administering the radio-isotope. Were these animals excluded from the behavioural analysis (as they should) or were they retained? This query needs clarification and an explicit statement added to the Methods.

Response.
We agree with Reviewer 2 that behavioural analysis should be reiterated without animals whose neurofunctional data were not acquired or useable. For the sake of conciseness and simplicity, these complementary results (see below) were reported in Suppl. S3.

Moreover, we have added in the main manuscript a statement referring to that issue (p. 14, l. 327):
“Due to technical problems (fails in proper intravenous radiotracer delivery at injection time), data from 23 mice (over 96) were not acquired or useable (EX/COC: n=5; EX/SAL: n=8; SED/COC: n=5; and SED/SAL: n=5). Note that the exclusion of those animals from behavioural analysis led to a similar pattern of results (reported in Suppl. S3).”

Thereafter is reported the Supplement 3:

Supplement 3. Comparisons of behavioural data between full samples (n=24/group) and samples without animals with missing neuro-functional data.

There was no evidence for statistically significant differences between the samples (Cohen’s d ranging from 0.02 to 0.45 with p-values ranging from .961 to .189).
Following tables: “Acute responsiveness”: number of crossings during the 1st cocaine injection minus number of crossings during baseline, “AUC ground”: refs for formula are cited in the main text, “Expression of sensitization”: number of crossings during the corresponding session.

Table S3.1 Mean (SD) — Exercised mice receiving cocaine.

	ALL mice (n=24)	n=5 excluded (n=19)	t-value	p-value	Cohen's d
Acute responsiveness	18 (36)	25 (37)	0.626	.535	0.19
AUC ground	1491 (450)	1504 (470)	0.092	.926	0.03
Expression of sensitization	194 (73)	193 (58)	0.048	.961	0.02

Table S3.2 Mean (SD) — Exercised mice receiving saline.

	ALL mice (n=24)	n=8 excluded (n=16)	t-value	p-value	Cohen's d
Acute responsiveness	-28 (39)	-13 (27)	1.337	.189	0.45
AUC ground	937 (237)	922 (253)	0.191	.849	0.06
Expression of sensitization	110 (34)	113 (36)	0.267	.791	0.09

Table S3.3 Mean (SD) — Sedentary mice receiving cocaine.

	ALL mice (n=24)	n=5 excluded (n=19)	t-value	p-value	Cohen's d
Acute responsiveness	70 (74)	76 (77)	0.259	.796	0.08
AUC ground	2020 (358)	2101 (335)	0.757	.453	0.23
Expression of sensitization	298 (66)	303 (71)	0.238	.812	0.07

Table S4.3 Mean (SD) — Sedentary mice receiving saline.

	ALL mice (n=24)	n=5 excluded (n=19)	t-value	p-value	Cohen's d
Acute responsiveness	-19 (36)	-18 (39)	0.087	.931	0.03
AUC ground	899 (309)	942 (331)	0.439	.662	0.13
Expression of sensitization	99 (48)	100 (50)	0.066	.947	0.02

For a complete response to the reviewer #2's comment, we reported thereafter the statistical analyses based on the samples without animals that missed the neuro-functional PET data, for the acute responsiveness, the overall responsiveness and the expression of sensitization.

Acute responsiveness (p.13, l. 301).
"Cocaine locomotor effect (vs saline, i.e., hyperlocomotor effect) was strong in each group (ts(81) = 3.85 and 7.39 with a probability of superiority (PS) of 72 % and 88 % in exercised and sedentary groups, respectively). However, this effect was significantly lower in exercised mice (planned contrasts: $\eta^2p = 0.073$, $t(81) = 2.51$, $p = .007$)."

Acute responsiveness — Results without animals with missing neuro-functional data:

	Contrast	t-value	p-value
EX-COC vs. EX-SAL	Simple	2.242	.028
SED-COC vs SED-SAL	Simple	5.871	<.001
EX-COC/EX-SAL vs. SED-COC/SED-SAL	Crossed	2.41	.009

Overall responsiveness (AUC ground) (p.13, l. 305).
"The pattern of results was comparable to that found for acute responsiveness. While cocaine effect was strong in each group (ts(81) = 5.47 and 11.07 with PS of 80 % and 96 % in exercised and sedentary groups, respectively), it was much lower in exercised mice (planned contrasts: $\eta^2p = 0.162$, $t(81) = 3.96$, $p < .001$)."

Overall responsiveness — Results without animals with missing neuro-functional data:

	Contrast	t-value	p-value
EX-COC vs. EX-SAL	Simple	4.775	<.001
SED-COC vs SED-SAL	Simple	9.923	<.001
EX-COC/EX-SAL vs. SED-COC/SED-SAL	Crossed	3.417	<.001

Expression of sensitization (p.13, l. 311).
"Consistent with previous experimental stages, long-term expression of the sensitized locomotor responsiveness was largely reduced in exercised mice (planned contrasts: $\eta^2p = 0.262$, $t(81) = 5.37$, $p < .001$). Again, cocaine effect was unambiguous in each group (ts(81) = 5.48 and 13.08 with PS of 80 % and 98 % in exercised and sedentary groups, respectively)."

Expression of sensitization — Results without animals with missing neuro-functional data:

	Contrast	t-value	p-value
EX-COC vs. EX-SAL	Simple	4.258	<.001
SED-COC vs SED-SAL	Simple	11.208	<.001
EX-COC/EX-SAL vs. SED-COC/SED-SAL	Crossed	4.703	<.001

Minor comment: Please add the species (mouse) to 'Subjects' (page 4).

The species has been added in the revised manuscript (p.4, l. 97): "Ninety-six 21-day-old C57BL/6J female mice were obtained from JANVIER".

1. de Wit H, Phillips TJ. Do initial responses to drugs predict future use or abuse? *Neurosci Biobehav Rev*. 2012;36(6). doi:10.1016/j.neubiorev.2012.04.005
2. Mantsch JR, Ho A, Schlussman SD, Kreek MJ. Predictable individual differences in the initiation of cocaine self-administration by rats under extended-access conditions are dose-dependent. *Psychopharmacology (Berl)*. 2001;157(1). doi:10.1007/s002130100744
3. Deminiere JM, Piazza P V., Le Moal M, Simon H. Experimental approach to individual vulnerability to psychostimulant addiction. *Neurosci Biobehav Rev*. 1989;13(2-3). doi:10.1016/S0149-7634(89)80023-5
4. Piazza PV, Deminière JM, Le Moal M, Simon H. Factors that predict individual vulnerability to amphetamine self-administration. *Science (80-)*. 1989;245(4925). doi:10.1126/science.2781295
5. Roberts AJ, Casal L, Huitron-Resendiz S, Thompson T, Tarantino LM. Intravenous cocaine self-administration in a panel of inbred mouse strains differing in acute

locomotor sensitivity to cocaine. *Psychopharmacology (Berl)*. 2018;235(4). doi:10.1007/s00213-018-4834-7
6. Tirelli E, Terry P. Amphetamine-induced conditioned activity and sensitization: The role of habituation to the test context and the involvement of Pavlovian processes. *Behav Pharmacol*. 1998;9(5-6). doi:10.1097/00008877-199809000-00004
7. Carey RJ, Gui J. Cocaine conditioning and cocaine sensitization: What is the relationship? *Behav Brain Res*. 1998;92(1). doi:10.1016/S0166-4328(97)00126-5
8. Crombag HS, Badiani A, Maren S, Robinson TE. The role of contextual versus discrete drug-associated cues in promoting the induction of psychomotor sensitization to intravenous amphetamine. *Behav Brain Res*. 2000;116(1). doi:10.1016/S0166-4328(00)00243-6
9. Stewart J, Badiani A. Tolerance and sensitization to the behavioral effects of drugs. In: *Behavioural Pharmacology*. Vol 4. ; 1993. doi:10.1097/00008877-199308000-00003
10. Stewart J, Vezina P. Extinction procedures abolish conditioned stimulus control but spare sensitized responding to amphetamine. *Behav Pharmacol*. 1991;2(1). doi:10.1097/00008877-199102000-00009
11. Drew KL, Glick SD. Characterization of the associative nature of sensitization to amphetamine-induced circling behavior and of the environment dependent placebo-like response. *Psychopharmacology (Berl)*. 1988;95(4). doi:10.1007/BF00172959
12. Mattingly BA, Gotsick JE. Conditioning and Experiential Factors Affecting the Development of Sensitization to Apomorphine. *Behav Neurosci*. 1989;103(6). doi:10.1037/0735-7044.103.6.1311
13. Szechtman H, Sulis W, Eilam D. Quinpirole induces compulsive checking behavior in rats: A potential animal model of obsessive-compulsive disorder (OCD). *Behav Neurosci*. 1998;112(6). doi:10.1037/0735-7044.112.6.1475
14. Mazurski EJ, Beninger RJ. Environment-specific conditioning and sensitization with (+)-amphetamine. *Pharmacol Biochem Behav*. 1987;27(1). doi:10.1016/0091-3057(87)90477-1
15. Mattingly BA, Himmler C, Bonta T, Rice LTL. Effects of selective dopamine D1- and D2-type receptor antagonists on the development of behavioral sensitization to 7-OH-DPAT. *Psychopharmacology (Berl)*. 1998;140(4). doi:10.1007/s002130050780
16. Reimer AR, Martin-Iverson MT. Nimodipine and haloperidol attenuate behavioural sensitization to cocaine but only nimodipine blocks the establishment of conditioned locomotion induced by cocaine. *Psychopharmacology (Berl)*. 1994;113(3-4). doi:10.1007/BF02245216
17. Michel A, Tirelli E. Conditioned hyperkinesia induced by cocaine in mice is dose-dependent but not correlated with the unconditioned response or the contextually-sensitized response. *Behav Pharmacol*. 2002;13(1). doi:10.1097/00008877-200202000-00006
18. Michel A, Tirelli E. Post-sensitisation conditioned hyperlocomotion induced by cocaine is augmented as a function of dose in C57BL/6J mice. *Behav Brain Res*. 2002;132(2). doi:10.1016/S0166-4328(01)00400-4
19. Tirelli E, Tambour S, Michel A. Sensitised locomotion does not predict conditioned locomotion in cocaine-treated mice: Further evidence against the excitatory conditioning model of context-dependent sensitisation. *Eur Neuropsychopharmacol*. 2003;13(4). doi:10.1016/S0924-977X(03)00037-3
20. Buckholtz JW, Treadway MT, Cowan RL, et al. Dopaminergic Network Differences in Human Impulsivity. *Science (80-)*. 2010;329(5991):532-532. doi:10.1126/science.1185778
21. Dalley JW, Fryer TD, Brichard L, et al. Nucleus accumbens D2/3 receptors predict trait

impulsivity and cocaine reinforcement. *Science (80-)*. 2007;315(5816):1267-1270. doi:10.1126/science.1137073
22. Caprioli D, Hong YT, Sawiak SJ, et al. Baseline-dependent effects of cocaine pre-exposure on impulsivity and D2/3 receptor availability in the rat striatum: possible relevance to the attention-deficit hyperactivity syndrome. *Neuropsychopharmacology*. 2013;38(8):1460-1471. doi:10.1038/npp.2013.44
23. Isles AR, Humby T, Walters E, Wilkinson LS. Common genetic effects on variation in impulsivity and activity in mice. *J Neurosci*. 2004;24(30):6733-6740. doi:10.1523/JNEUROSCI.1650-04.2004
24. Gubner NR, Wilhelm CJ, Phillips TJ, Mitchell SH. Strain differences in behavioral inhibition in a go/No-go task demonstrated using 15 inbred mouse strains. *Alcohol Clin Exp Res*. 2010;34(8):1353-1362. doi:10.1111/j.1530-0277.2010.01219.x
25. Jupp B, Caprioli D, Dalley JW. Highly impulsive rats: Modelling an endophenotype to determine the neurobiological, genetic and environmental mechanisms of addiction. *DMM Dis Model Mech*. 2013;6(2):302-311. doi:10.1242/dmm.010934
26. Tsai YJ, Hsieh SS, Huang CJ, Hung TM. Dose-Response Effects of Acute Aerobic Exercise Intensity on Inhibitory Control in Children With Attention Deficit/Hyperactivity Disorder. *Front Hum Neurosci*. 2021;15(June):1-13. doi:10.3389/fnhum.2021.617596
27. Volkow ND, Wang GJ, Fowler JS, et al. Reinforcing effects of psychostimulants in humans are associated with increases in brain dopamine and occupancy of D2 receptors. *J Pharmacol Exp Ther*. Published online 1999.
28. Sams-Dodd F. Phencyclidine in the social interaction test: An animal model of schizophrenia with face and predictive validity. *Rev Neurosci*. 1999;10(1). doi:10.1515/REVNEURO.1999.10.1.59
29. Lespine LF, Tirelli E. The protective effects of free wheel-running against cocaine psychomotor sensitization persist after exercise cessation in C57BL/6J mice. *Neuroscience*. 2015;310:650-664. doi:10.1016/j.neuroscience.2015.10.009
30. Lespine LF, Tirelli E. Evidence for a long-term protection of wheel-running exercise against cocaine psychomotor sensitization in adolescent but not in adult mice. *Behav Brain Res*. 2018;349(May):63-72. doi:10.1016/j.bbr.2018.04.054
31. Engelmann AJ, Aparicio MB, Kim A, et al. Chronic wheel running reduces maladaptive patterns of methamphetamine intake: regulation by attenuation of methamphetamine-induced neuronal nitric oxide synthase. *Brain Struct Funct*. 2014;219(2):657-672. doi:10.1007/s00429-013-0525-7
32. Nishijima T, Llorens-Martín M, Tejeda GS, et al. Cessation of voluntary wheel running increases anxiety-like behavior and impairs adult hippocampal neurogenesis in mice. *Behav Brain Res*. 2013;245:34-41. doi:10.1016/j.bbr.2013.02.009
33. Greenwood BN, Loughridge AB, Sadaoui N, Christianson JP, Fleshner M. The protective effects of voluntary exercise against the behavioral consequences of uncontrollable stress persist despite an increase in anxiety following forced cessation of exercise. *Behav Brain Res*. 2012;233(2):314-321. doi:10.1016/j.bbr.2012.05.017
34. Lespine LF, Plenevaux A, Tirelli E. Wheel-running exercise before and during gestation against acute and sensitized cocaine psychomotor-activation in offspring. *Behav Brain Res*. 2019;363(August 2018):53-60. doi:10.1016/j.bbr.2019.01.049
35. Rosenthal R, Rosnow RL. *Contrast Analysis : Focused Comparisons in the Analysis of Variance*. Cambridge : Cambridge university press; 1985. http://lib.ugent.be/catalog/rug01:001897715
36. Belin D, Deroche-Gamonet V, Jaber M. Cocaine-induced sensitization is associated with altered dynamics of transcriptional responses of the dopamine transporter, tyrosine

hydroxylase, and dopamine D2 receptors in C57Bl/6J mice. *Psychopharmacology (Berl)*. 2007;193(4):567-578. doi:10.1007/s00213-007-0790-3
37. Narendran R, Martinez D. Cocaine abuse and sensitization of striatal dopamine transmission: a critical review of the preclinical and clinical imaging literature. *Synapse*. 2008;62(11):851-869. doi:10.1002/syn.20566
38. Robinson TE, Jurson PA, Bennett JA, Bentgen KM. Persistent sensitization of dopamine neurotransmission in ventral striatum (nucleus accumbens) produced by prior experience with (+)-amphetamine: a microdialysis study in freely moving rats. *Brain Res*. 1988;462(2). doi:10.1016/0006-8993(88)90549-5
39. Kolta MG, Shreve P, De Souza V, Uretsky NJ. Time course of the development of the enhanced behavioral and biochemical responses to amphetamine after pretreatment with amphetamine. *Neuropharmacology*. 1985;24(9). doi:10.1016/0028-3908(85)90032-2
40. Briand LA, Flagel SB, Seeman P, Robinson TE. Cocaine self-administration produces a persistent increase in dopamine D2 High receptors. *Eur Neuropsychopharmacol*. 2008;18(8):551-556. doi:10.1016/j.euroneuro.2008.01.002
41. Lu L, Grimm JW, Shaham Y, Hope BT. Molecular neuroadaptations in the accumbens and ventral tegmental area during the first 90 days of forced abstinence from cocaine self-administration in rats. *J Neurochem*. 2003;85(6). doi:10.1046/j.1471-4159.2003.01824.x
42. Vanderschuren LJMJ, Kalivas PW. Alterations in dopaminergic and glutamatergic transmission in the induction and expression of behavioral sensitization: a critical review of preclinical studies. *Psychopharmacology (Berl)*. 2000;151(2-3):99-120. doi:10.1007/s002130000493
43. Pierce RC, Kalivas PW. A circuitry model of the expression of behavioral sensitization to amphetamine-like psychostimulants. *Brain Res Rev*. 1997;25(2):192-216. doi:10.1016/S0165-0173(97)00021-0
44. Mirrione MM, Schiffer WK, Fowler JS, Alexoff DL, Dewey SL, Tsirka SE. A novel approach for imaging brain–behavior relationships in mice reveals unexpected metabolic patterns during seizures in the absence of tissue plasminogen activator. *Neuroimage*. 2007;38(1):34-42. doi:10.1016/j.neuroimage.2007.06.032
45. Ma Y, Hof PR, Grant SC, et al. A three-dimensional digital atlas database of the adult C57BL/6J mouse brain by magnetic resonance microscopy. *Neuroscience*. 2005;135:1203-1215. doi:10.1016/j.neuroscience.2005.07.014
46. De Vries T. Relapse to Cocaine- and Heroin-Seeking Behavior Mediated by Dopamine D2 Receptors Is Time-Dependent and Associated with Behavioral Sensitization. *Neuropsychopharmacology*. 2002;26(1):18-26. doi:10.1016/S0893-133X(01)00293-7
47. Zhou Y, Zhao M, Zhou C, Li R. Sex differences in drug addiction and response to exercise intervention: From human to animal studies. *Front Neuroendocrinol*. 2016;40:24-41. doi:10.1016/j.yfrne.2015.07.001
48. Rhodes JS, Jeffrey S, Girard I, et al. Exercise increases hippocampal neurogenesis to high levels but does not improve spatial learning in mice bred for increased voluntary wheel running. *Behav Neurosci*. 2003;117(5). doi:10.1037/0735-7044.117.5.1006
49. Koteja P, Garland T, Sax JK, Swallow JG, Carter PA. Behaviour of house mice artificially selected for high levels of voluntary wheel running. *Anim Behav*. 1999;58(6). doi:10.1006/anbe.1999.1270
50. Harri M, Lindblom J, Malinen H, et al. Effect of access to a running wheel on behavior of C57BL/6J mice. *Lab Anim Sci*. Published online 1999.
51. Pietropaolo S, Mintz M, Feldon J, Yee BK. The Behavioral Sequela Following the Prevention of Home-Cage Grid-Climbing Activity in C57BL/6 Mice. *Behav Neurosci*.

2007;121(2). doi:10.1037/0735-7044.121.2.345
52. Mustroph ML, Stobaugh DJ, Miller DS, DeYoung EK, Rhodes JS. Wheel running can accelerate or delay extinction of conditioned place preference for cocaine in male C57BL/6J mice, depending on timing of wheel access. *Eur J Neurosci*. 2011;34(7):1161-1169. doi:10.1111/j.1460-9568.2011.07828.x
53. Smith MA, Pitts EG. Wheel running decreases the positive reinforcing effects of heroin. *Pharmacol Reports*. 2012;64(4):960-964. doi:10.1016/S1734-1140(12)70891-5
54. Pietropaolo S, Sun Y, Li R, Brana C, Feldon J, Yee BK. The impact of voluntary exercise on mental health in rodents: A neuroplasticity perspective. *Behav Brain Res*. 2008;192(1). doi:10.1016/j.bbr.2008.03.014
55. Pietropaolo S, Feldon J, Alleva E, Cirulli F, Yee BK. The role of voluntary exercise in enriched rearing: A behavioral analysis. *Behav Neurosci*. 2006;120(4). doi:10.1037/0735-7044.120.4.787
56. Dubreucq S, Marsicano G, Chaouloff F. Duration- and environment-dependent effects of repeated voluntary exercise on anxiety and cued fear in mice. *Behav Brain Res*. 2015;282. doi:10.1016/j.bbr.2014.12.042
57. Dubreucq S, Marsicano G, Chaouloff F. Emotional consequences of wheel running in mice: Which is the appropriate control? *Hippocampus*. 2011;21(3). doi:10.1002/hipo.20778
58. Bednarczyk MR, Hacker LC, Fortin-Nunez S, Aumont A, Bergeron R, Fernandes KJL. Distinct stages of adult hippocampal neurogenesis are regulated by running and the running environment. *Hippocampus*. 2011;21(12). doi:10.1002/hipo.20831
