# Supplementary material for: Exercise against cocaine sensitization in mice: a [18F]fallypride micro-PET study
Source: Brain Commun. 2021 Dec 15;4(1):fcab294. doi: 10.1093/braincomms/fcab294 (PMC8833578; doi:10.1093/braincomms/fcab294)
Supplement: fcab294_Supplementary_Data [file Revision 1 GÇô Decision Letter.pdf › Revision 1 GÇô Decision Letter.pdf]

Revision 1 – Decision Letter

01-Nov-2021

Dear Dr. Becker,

It is a pleasure to accept your manuscript entitled "Exercise Against Cocaine Sensitization In Mice: A [18F]Fallypride Micro-PET Study" for publication in Brain Communications. Any comments made by the reviewer(s) are included below.

The next step is for your paper to go through "First Look". We will contact you again shortly if any final files or formatting changes are required for publication or if we have any queries.

If no changes are needed, your submission will be transferred to our Production Department and you will hear from the Production Editor in due course.

Video abstracts - Brain Communications encourages authors to provide a video abstract to accompany their article. These are hosted on the Brain/Brain Communications YouTube channel. If you are interested in producing a 5-min video abstract to promote your article, then please contact the Editorial Office (brcoms.editorialoffice@oup.com) for further details and guidelines.

Thank you very much for your contribution. On behalf of the Editors of Brain Communications, we look forward to your future contributions to the Journal.

Best wishes

Dr. David Belin
Associate Editor, Brain Communications

Professor Tara Spires-Jones
Founding Editor, Brain Communications

Reviewer(s)' Comments to Author:

Reviewer: 2

Comments to the Author
The authors have adequately addressed my comments.

Reviewer: 1

Comments to the Author
The authors engaged in a very constructive peer-review process in which they adequately addressed my concerns.

They should be commended for the quality of their rebuttal letter.
Well done!
