# Supplementary material for: Exercise against cocaine sensitization in mice: a [18F]fallypride micro-PET study
Source: Brain Commun. 2021 Dec 15;4(1):fcab294. doi: 10.1093/braincomms/fcab294 (PMC8833578; doi:10.1093/braincomms/fcab294)
Supplement: fcab294_Supplementary_Data [file Original Submission-Decision Letter.pdf › Original Submission-Decision Letter.pdf]

Original Submission – Decision Letter

02-Aug-2021

Dear Dr. Becker,

Thank you for your recent submission BRAINCOM-2021-197 entitled "Exercise Against Cocaine Sensitization In Mice: A [18F]Fallypride Micro-PET Study" to Brain Communications.

Your manuscript has been reviewed, and while the reviewers and editorial team appreciate many aspects of the paper, they raise some concerns that you will need to address in order for the paper to be considered for publication.

If you can address these concerns, I invite you to submit a revised manuscript and a response to each of the reviewers' comments. The reviewer(s)' comments are included at the bottom of this message.

In addition to the reviewers' comments, please note our editorial comments added below to comply with our requirements for presentation of scientific data at Brain Communications.

Please format your revised manuscript according to our Instructions to Authors https://academic.oup.com/braincomms/pages/General_Instructions – a summary of key points is attached to help with this.

To revise your manuscript, click the link below or log into https://mc.manuscriptcentral.com/braincom and enter your Author Centre, where you will find your manuscript title listed under "Manuscripts with Decisions." Under "Actions," click on "Create a Revision." Your manuscript number will be appended to denote a revision.

*** PLEASE NOTE: This is a two-step process. After clicking on the link, you will be directed to a webpage to confirm. ***

https://mc.manuscriptcentral.com/braincom?URL_MASK=df98d9132eea4b4e9992dc295ce7ead3

Please upload a revised manuscript with changes tracked with track changes or in coloured text.

When submitting your revised manuscript, you will be asked to respond to the reviewer(s) comments. Please be as specific as possible in your response to all of the comments.

The editorial team at Brain Communications aims to increase transparency in scientific publishing and to demonstrate the value that peer review brings to the scientific process. Please therefore consider whether you are willing to have reviewer(s)' comments on your paper and your response published as supplementary data with the paper if it is accepted. This is entirely optional and will only be done if both the authors and reviewers agree. While submitting your revision there will be an opportunity to review or update the answer you gave previously, if necessary.

IMPORTANT: Your original files are available to you when you upload your revised manuscript. Please delete any redundant files before completing the submission.

Because we are trying to facilitate timely publication of manuscripts submitted to Brain Communications, we would appreciate your revised manuscript as soon as possible. If it is not possible for you to resubmit within 3 months, the option to submit a revision will expire, and we would then consider your revised paper as a new submission. However, if you need a little more time, please get in touch and we can extend this deadline for you.

Once again, thank you for submitting your manuscript to Brain Communications and I look forward to receiving your revision.

Best wishes

Dr. David Belin
Associate Editor, Brain Communications

Professor Tara Spires-Jones
Founding Editor, Brain Communications

Reviewer(s)' Comments to Author:

Reviewer: 1

Comments to the Author
In this study Becker et al. investigated the correlates in the striatum of the influence of exercise on cocaine sensitisation measuring D2/3 dopamine receptor binding using PET in mice.
There are several issues with this study that preclude any consideration for publication:

1. The relevance of a sensitised locomotor response to non-contingent, experimenter-delivered administration of cocaine to the understanding of addiction remains elusive. It would be very helpful to have a better justification than a citation related to its potential relevance for relapse and craving: is relapse and/or craving (a subjective state) somewhat related to the locomotor activating effects of cocaine in humans?
Sensitisation is a behavioural manifestation of an anticipatory pavlovian response (conditioned locomotion added to the activating effects of the drug over time), but it is not presented as such in this study, thereby making it difficult to understand what is being discussed.
2. Is it the case that a differential sensitivity to the reinforcing properties of addictive drugs is a valid marker of vulnerability to addiction?
3. Is it not the case that high impulsivity trait, a marker of addition vulnerability in humans and rodents, is associated with decreased D2/3 striatal binding, as measured with PET?
4. In humans, the subjective report of euphoria or high associated with the administration of methylphenidate is negatively correlated with the level of D2/3 DA receptors in the striatum (see elegant work from Volkow). How do the authors reconcile the effect of exercise on D2/3 receptors levels and their suggestion that it represents a biological signature of the protective effect of exercise on addiction vulnerability?
5. Could it be the case that mice used to run on a wheel then exclusively run on a wheel (perhaps accounting for the seemingly lower level of baseline locomotor activity in EX mice as compared to SED mice – effect not reported in the manuscript), hence it would be very informative to provide a wheel in the test context at the time the expression of the sensitized response to cocaine is measured.
6. According to T. Robinson, the behavioural manifestation of sensitisation follows an inverted u-shaped curve in that animals will stop running to engage in an ever narrowing repertoire of stereotypic behaviours when sensitised. Have these behaviours been measured here?
7. Why were the wheels only removed in half the EX population prior to the PET session? Rather than controlling for a potential effect of wheel running on the outcome of the PET measurements, the removal of the wheel can have triggered a massive stress in animals that have had access to it daily. This is a major confounding factor.
8. The absence of a sal vs coc design at test (expression of sensitization) makes it difficult to appreciate the magnitude of sensitization here: what is the effect of an acute cocaine injection in mice with a history of repeated saline administration and associated exposure to the novel test box and, more importantly, what is the magnitude of the conditioned response to the injection in cocaine treated mice (which can only be revealed in a coc-treatment – sal-challenge group)?
9. The justification for an approach by contrasts, instead of a post-hoc interrogation of the main effects is not clear. Why would the pairwise comparisons not be carried with a Newman Keuls or a Sidak/Tuckey?
10. PET does not distinguish between states of DARs, as well pointed out in the manuscript. It does not distinguish either between pre- and post-synaptic receptors. When searching through the literature on D2 DA receptors in VTA vs SNc neurons, it appears that an up-regulation of D2R mRNA occurs in mice with a history of cocaine sensitization in dopaminergic neurons per se (10.1007/s00213-007-0790-3).

Reviewer: 2

Comments to the Author
This interesting study examines the interaction between exercise and PET-derived measures of striatal D2R binding in the context of cocaine-induced behavioural sensitization. Using a cross-sectional 2x2 factorial design, the authors provide evidence that exercise in the form of wheel running in mice, attenuates sensitized increases in locomotor activity and D2R binding potential in the dorsal striatum. The study as a whole represents a considerable amount of work, especially in relation to the technical accomplishment of in-vivo PET imaging in mice. However, there are a number of issues that dampen my enthusiasm for this work and what it can tell us about real-world addiction in humans.

Firstly, the authors need a clearer rationale for studying sensitization as an explanatory mechanism for addiction. The introduction is written in such a way to suggest that the experimental phenomenon of sensitization is central to the persistence of drug use. The authors may wish to be more circumspect about the necessary and sufficient involvement of sensitization in addiction and moreover take a less

dopamine-centric view of underlying neurochemical mechanisms.

Secondly, and more problematically, the findings run counter to the rich corpus of studies showing reduced D2Rs as a striatal biomarker of addiction. This discrepancy should be addressed, possibly at the level of the experimental protocol used in the present study. It would be helpful if the authors referred to the work of Isabelle Boileau (2006), one of the first PET demonstrations of sensitization in humans. Of note, in this study, no changes in striatal D2R binding potential were reported following several doses of d-amphetamine (discussed on page 1392 – Boileau et al., 2006).

Unfortunately, the interpretation of the present findings is constrained by scanning at a single time point only. The true power of PET lies in longitudinal assessment - in this case, pre and post cocaine. It is a moot point whether the observed increase in D2R binding potential would persist for days or even weeks; it may well decompensate after an extended period. The authors should acknowledge this point and discuss any relevant evidence for the temporal dynamics of D2R binding potential in experimental approaches of sensitization in rodents, including D3Rs.

Thirdly, can the authors comment on whether the increase in D2R binding potential was a presynaptic or post-synaptic phenomenon. It is puzzling why the dorsal striatum was selected given overwhelming evidence implicating the mesolimbic dopamine system in sensitization (as the authors indeed acknowledge). A midbrain ROI would provide insight into presynaptic D2 receptors. A ventral striatal ROI would inform the spatial and possible temporal development of sensitization.

Fourthly, I don’t understand the logic of only assessing female mice. The replication of apparently sexually-dimorphic effects is important. The reliance on sensitization coupled with the singular analysis of females severely restricts the generality of the present findings.

Fifthly, can the authors really be sure their findings implicate exercise per se or more likely environmental enrichment?

Finally, it is noted that 23 mice were excluded due to technical difficulties administering the radio-isotope. Were these animals excluded from the behavioural analysis (as they should) or were they retained? This query needs clarification and an explicit statement added to the Methods.

Minor comment: Please add the species (mouse) to ‘Subjects’ (page 4).

Editorial Office comments:

-For all the plots: please report in the figure legend the test used and relevant test statistic values alongside p values (for example F or t values for ANOVAs and t-tests).

-Please supply a graphical abstract. A graphical abstract is a visual representation of the central finding or methodology of the paper. It will be displayed in the table of contents. Authors should strive to make the graphical abstract informative, interesting, visually appealing, and straightforward (no caption needed, no abbreviations except gene names). Please upload as a separate high resolution image file (tiff preferred). The technical requirements for the graphical abstracts are:
 --size of the submitted image: 1200 pixels square at 300dpi
 --font of the text: Arial 12-16 point (smaller fonts will not be legible online)

-Please add an Abbreviated Summary (up to 50 words) of your paper that captures the main purpose and conclusions of the work. The summary should state the main results written in the third person (‘[1st author surname] et al. report that …’) and conclusion. This summary will be used in the contents list online and should not contain any abbreviations other than those on the accepted abbreviations list and accepted gene/protein names, making it accessible to the non-specialist. Please upload it as a Word document. Allowed abbreviations: AIDS; ANOVA; AMPA; ATP; A,T,C,G; CNS; CSF; CT; DNA; ECG; EEG; EMG; GABA; HIV; MRI; NMDA; PET; PCR; RNA.

- To comply with our ethical standards, the appropriate checklist must be included (see Reporting Guideline section within 'Instructions to authors').
